# Mapping dynamic QTL dissects the genetic architecture of grain size and grain filling rate at different grain-filling stages in barley

**DOI:** 10.1038/s41598-019-53620-5

**Published:** 2019-12-11

**Authors:** Binbin Du, Qifei Wang, Genlou Sun, Xifeng Ren, Yun Cheng, Yixiang Wang, Song Gao, Chengdao Li, Dongfa Sun

**Affiliations:** 1 College of Plant Science and Technology, Huazhong Agricultural University, Wuhan, 430070 China; 2 Biology Department, Saint Mary’s University, 923 Robie Street, Halifax, NS B3H 3C3 Canada; 3 Department of Agriculture & Food/Agricultural Research Western Australia, 3 Baron-Hay Court, South Perth, WA 6155 Australia; 4 Hubei Collaborative Innovation Center for Grain Industry, Yangtze University, Jingzhou, 434025 Hubei China

**Keywords:** Agricultural genetics, Plant breeding

## Abstract

Grain filling is an important growth process in formation of yield and quality for barley final yield determination. To explore the grain development behavior during grain filling period in barley, a high-density genetic map with 1962 markers deriving from a doubled haploid (DH) population of 122 lines was used to identify dynamic quantitative trait locus (QTL) for grain filling rate (GFR) and five grain size traits: grain area (GA), grain perimeter (GP), grain length (GL), grain width (GW) and grain diameter (GD). Unconditional QTL mapping is to detect the cumulative effect of genetic factors on a phenotype from development to a certain stage. Conditional QTL mapping is to detect a net effect of genetic factors on the phenotype at adjacent time intervals. Using unconditional, conditional and covariate QTL mapping methods, we successfully detected 34 major consensus QTLs. Moreover, certain candidate genes related to grain size, plant height, yield, and starch synthesis were identified in six QTL clusters, and individual gene was specifically expressed in different grain filling stages. These findings provide useful information for understanding the genetic basis of the grain filling dynamic process and will be useful for molecular marker-assisted selection in barley breeding.

## Introduction

Barley grain filling is an important growth process for yield and quality, which determines the thousand grain weight (TGW) and final yield^1^. Grain filling is mainly determined by filling rate and duration^2^. Genotypic variation between these two parameters have been reported in barley^3–8^. Metzger *et al*.^9^ found differences in the grain-filling duration between barley varieties, but no significant correlation between grain-filling duration and yield. Moreover, grain weight was correlated positively with grain filling rate, but not with grain filling duration in wheat and maize^10,11^.

Previous reports suggested that the grain filling rate was better than grain filling duration to reveal the difference in yield^7,12,13^. Grain filling duration is vulnerable to temperature, especially under stress conditions^14–17^. In addition, long-term grain filling affected the normal sowing of stubble crops and the regular maturation of barley, whereas the grain filling rate (GFR) seems to be dominated by the genetic factors^6,7,12^. Therefore, increasing grain filling rate was more important than increasing grain filling duration for crop yield.

The grain filling process can be divided into three periods: grain formation, linear growth and maturity period^18^. The grain formation period was an active phase of cell division with little accumulation of dry matter^19^. After entering the linear growth period, the accumulation of dry matter increases rapidly, and the GFR continues to rise and reaches its maximum value^20^. At the end of the linear growth period, the grain weight increases slowly, and the GFR decreases significantly, reaching the maturity period^21^. Although the environment such as temperature and humidity affects GFR during the filling process, genotype is still the main factor affecting GFR^7,22^.

Physiological mechanisms of grain filling have been extensively studied in barley^23–25^, but few reported the genetic basis for grain filling characteristics. To date, QTL for yield-related traits such as, grain per spike, grain weight per spike, grain size and thousand grain weight have been reported in barley^26–30^. The above mentioned traits were conventional QTL based on the final phenotype after maturation. However, many morphological traits are dynamic or progressive development^31^. According to the theory of developmental genetics, genes are selectively expressed at different growth stages, QTL mapping of these morphological traits should be analyzed to find out the authentic pattern of function of genes at different developmental stages^32^. Zhu^33^ proposed a method to map conditional QTL using the net genetic effect between two time points to reveal the gene interactions and regulatory mechanisms of quantitative traits in crop development. Since then, conditional QTL mapping methods have been used to study the agronomic traits, such as root growth and seed vigour in rice^34,35^, plant height and protein content in wheat^36–38^, and dry matter accumulation in soybean^39^.

The final yield and yield components are the result of a combination of physiological and biochemical processes, a genetic analysis of grain weight or size traits might not provide a reasonable explanation for yield determinants^40^, however, grain filling rate is crucial in determining the grain weight and yield during the physiological and biochemical processes of yield formation. In this study, a DH population containing 122 lines was used to study dynamic QTLs for grain-filling traits in barley. The objectives were to: (1) identify QTL for grain filling rate and grain size traits using conventional and conditional mapping methods, (2) explore the genetic basis of grain filling dynamic process in barley.

## Materials and Methods

### Plant materials and field experiment

The mapping population comprised 122 doubled haploid (DH) lines was obtained from anther culture by Huaai11 × Huadamai6^41^. Huaai11 is a six-rowed dwarf barley variety selected from the barley landrace Daofu Baiqingke, Huadamai6 is an elite two-rowed feed barley developed by Huazhong Agricultural University. Besides, there were differences in GFR and grain size between two parents. In 2017 (Y1) and 2018 (Y2), the DH population and two parents were planted at the farm of Huazhong Agricultural University (Wuhan, 114°30′E, 30°60′N). The climatic conditions of the barley growing season during the two years are described in Supplementary Fig. S1. The field trial followed a completely randomized block design, with 3 replicates each year. All DH and parents were grown in two rows with a length of 1.5 m and 0.2 m spacing plots and 30 seeds in each row. The management of field experiments was in line with local standard practices.

### Traits measurement

The traits evaluated in this study include: thousand grain weight (TGW), grain filling rate (GFR), mean grain filling rate (GFR_mean_), maximum grain filling rate (GFR_max_), grain area (GA), grain perimeter (GP), grain length (GL), grain width (GW) and grain diameter (GD), the measurements were as follows:

At the flowering time, 40 spikes that were basically the same in the growth, size and flowering time were tagged for each line. Five tagged spikes were randomly sampled from each line at 7, 14, 21, 28, 35, 42 and 49 days after anthesis in 2017 and 2018, respectively. For convenience, the seven sampling stages were named as I, II, III, IV, V, VI and VII, respectively. The chaff of grain was peeled off and the GA, GP, GL, GW and GD traits were evaluated using WSeen SC-G automatic seed selection and thousand grain weight analysis system, then the grains were put at 105 °C for 15 minutes and dried at 65 °C until constant weight. The TGW was recorded from the first stage to the seventh stage, and the grain size traits were recorded from the second stage because the first sample was too small to be measured. The GFR (between two sampling stages) was calculated as: GFR = TGW of the difference between two sampling times/7. The grain filling process was adjusted by logistic equation (Y = K/(1 + ae^−bt^) using the days after flowering (t) as independent variable and grain weight (Y) as dependent variable, K was the maximum theoretical weight, a and b were modulus calculated by the regression equation. These parameters were determined according to the SAS NLIN procedure. The GFR_mean_ and GFR_max_ parameters were calculated by the first and second order derivatives of the logistic equation, GFR_max_ = -Kb/4, and GFR_mean_ = GW_max_/GFD, GFD is the number of days from flowering to maturity of the plant, GW_max_ is the maximum grain weight in GFD^42^.

### Phenotypic data statistics

The phenotypic data was analyzed using SAS v.9.2 (SAS Institute Inc, Cary, NC). Correlation analysis between the traits was performed using the “PROC CORR” program. ANOVA of each component was performed using PROC GLM procedure. The broad-sense heritability was calculated using the formula: *h*_*B*_^2^ = *σ*_*g*_^2^/(*σ*_*g*_^2^ + *σ*_*ge*_^2^/*n* + *σ*_*e*_^2^*/rn)*, where *σ*_*g*_^2^ was the genetic variance, *σ*_*ge*_^2^ was the genetic-by environment interaction variance, *σ*_*e*_^2^ was the error variance, and r and n were the number of repetitions for each genotype and environment, respectively.

### Dynamic QTL analysis

The genetic map consisting of the 1962 markers cover a total of 1375.8 cM genomic regions was used to screen QTLs^43^. Unconditional phenotypic values were measured at different stages of I, II, III, IV, V, VI and VII after flowering time. Conditional phenotypic values were the incremental grain filling-related traits values in adjacent stages ΔT2 (II-I), ΔT3 (III-II), ΔT4 (IV-III), ΔT5 (V-IV), ΔT6 (VI-V) and ΔT7 (VII-VI), since the grain size traits were recorded from stage II, so the conditional phenotypic values were calculated from ΔT3, and the data collection method was used according to Zhu^33^. The conditional phenotypic value ΔT1 of GFR from the flowering time to the stage I was the phenotypic value of stage I, so the effects were considered to be the unconditional genetic effects of stage I. QTL IciMapping 4.1 with inclusive composite interval mapping (ICIM) model was used to analyze the locus and effects of unconditional QTLs^44,45^. Conditional variable analysis method^33^ combined with ICIM was used to perform conditional QTL analysis of GA, GP, GL, GW, GD and GFR at each stage. Furthermore, in order to eliminate the interference of row type (Rt) and caryopsis type (Ct), we used QTL.gCIMapping.GUI software^46^ for covariate QTL analysis using Rt and Ct as covariates. The scan step and probability in stepwise regression (PIN) were set to 1 cM and 0.001, respectively. The logarithm of the odds (LOD) threshold was set to 3.0 after 1000 permutations on a 0.05 Type 1 error, so the QTL was declared based on the LOD threshold of 3.0, and these QTLs were termed ‘identified QTLs’.

### QTL integration

Goffinet and Gerber^47^ first used the QTL meta-analysis method to integrate QTLs from independent experiments and determine the corresponding confidence intervals for consensus QTLs, this method is currently the best way to solve QTL integration. In this study, BioMercator 4.2 was used to integrate QTLs identified in different years or stages and to determine the optimal number of consensus QTLs based on meta-analysis method^48^. If an identified QTL did not overlap CI with other QTLs, it was also considered a consensus QTL. According to Wang *et al*.^49^, the “two-round” QTL integration strategy was adopted here with minor modifications. In the first round, the unconditional and conditional identified QTLs for each trait in two environments were integrated into unconditional and conditional consensus QTLs using BioMercator 4.2. In the second round, these unconditional and conditional consensus QTLs for each trait of the first round of integration were integrated into a unique QTLs. The name of the consensus QTL follows the nomenclature rules reported by McCouch^50^. The designation was prefixed with ucq, cq, uq and qc followed by abbreviation of a trait, and a linkage group to represent the unconditional, conditional, unique QTL and covariate QTL, respectively. If two or more consensus QTLs were detected in the linkage group, a hyphen ‘-’ with a serial number was added to the linkage group. For example, QTL *cqGFR1-*2 indicates the second conditional consensus QTL for GFR on chromosome 1H. The phenotypic variation explained (PVE) by consensus QTL more than 20% or at least twice with PVE ≥ 10% was considered to be a major QTL, otherwise regarded as a minor QTL^51^. The unconditional and conditional consensus QTLs location on genetic map were drawn using MapChart ver. 2.2 software^52^.

## Results

### Phenotypic variation

**Figure 1:**
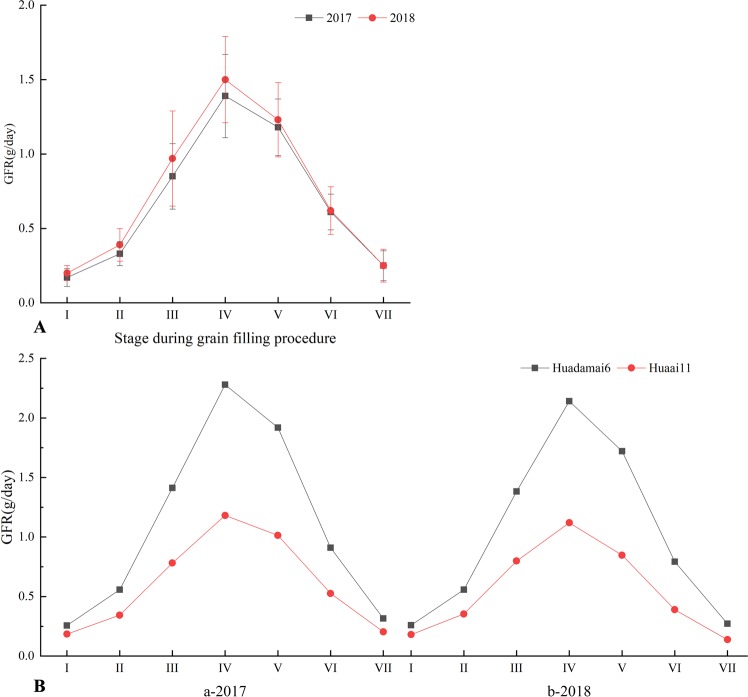
Grain filling rate for parents (**B**) and DH population (**A**) at different grain filling stages in 2017 and 2018 at Wuhan, China.

The phenotypic data of the parents and DH line were listed in Table 1. The value of each trait in Huadamai6 was significantly higher than Huai11 (P < 0.01) except for GW at stage II. For the two parents, GFR increased slowly during the initial two stages, after which GFR increased rapidly and reached its maximum at stage IV, and then decreased continuously in two years (Fig. 1B). In the DH population, the tendency of GFR in the seven sampling stages was basically the same as that of the parents (Fig. 1A). The frequency distribution of the five grain size traits was shown in Supplementary Fig. S2. Each trait was continuous variation in all sampling stages, and the absolute values of skewness and kurtosis of most traits were less than 1 except for GFR at stage VII, indicating that these traits followed a normal distribution and were suitable for QTL analysis (Table 1). ANOVA showed statistically significant effects for genotype, environment and G × E interaction with all traits (P < 0.01) (Supplementary Table S1). The broad-sense heritability (*h*_*B*_^2^) was estimated between 58.9% and 98.5% (Supplementary Table S1).

**Table 1:** Phenotypic values of grain filling rate and grain size traits in parents and DH population at different grain filling stages over two years.

Year	Trait^a^	Stage^b^	Huaai11	Huadamai6	ST^c^	DH lines			
			Mean	Mean		Range	Mean	Skew	Kurt
2017	GFR	I	0.18 ± 0.02	0.26 ± 0.01	0.000^**^	0.03–0.39	0.17 ± 0.01	0.44	−0.20
		II	0.34 ± 0.04	0.56 ± 0.01	0.000^**^	0.08–0.76	0.33 ± 0.01	0.86	0.93
		III	0.78 ± 0.03	1.41 ± 0.03	0.000^**^	0.32–2.21	0.85 ± 0.03	0.97	0.73
		IV	1.18 ± 0.04	2.28 ± 0.07	0.000^**^	0.47–2.80	1.39 ± 0.05	0.72	−0.64
		V	1.01 ± 0.04	1.92 ± 0.5	0.000^**^	0.55–2.55	1.18 ± 0.04	1.08	0.78
		VI	0.53 ± 0.03	0.91 ± 0.04	0.000^**^	0.13–1.33	0.61 ± 0.02	0.39	0.12
		VII	0.20 ± 0.01	0.32 ± 0.04	0.000^**^	0.02–0.73	0.25 ± 0.01	0.91	0.40
	GFR_mean_		0.60 ± 0.04	1.09 ± 0.04	0.000^**^	0.36–1.16	0.69 ± 0.02	0.68	−0.43
	GFR_max_		1.21 ± 0.02	2.45 ± 0.05	0.000^**^	0.57–3.14	1.54 ± 0.06	0.76	−0.43
	GA	II	6.39 ± 0.08	7.53 ± 0.15	0.002^**^	3.69–10.00	6.43 ± 0.13	0.28	−0.48
		III	8.72 ± 0.11	12.06 ± 0.18	0.000^**^	5.33–14.86	9.45 ± 0.17	0.50	−0.01
		IV	11.39 ± 0.08	16.32 ± 0.25	0.000^**^	8.48–18.12	12.80 ± 0.23	0.52	−0.90
		V	12.09 ± 0.12	20.12 ± 0.21	0.000^**^	10.11–22.65	15.28 ± 0.26	0.72	−0.24
		VI	14.53 ± 0.15	22.26 ± 0.26	0.000^**^	11.89–25.61	17.08 ± 0.30	0.98	0.29
		VII	14.64 ± 0.14	22.55 ± 0.18	0.000^**^	11.26–25.68	17.04 ± 0.30	0.90	0.17
	GP	II	12.13 ± 0.08	14.51 ± 0.11	0.000^**^	7.78–16.62	12.08 ± 0.15	−0.11	−0.42
		III	14.22 ± 0.13	16.26 ± 0.11	0.000^**^	11.53–18.09	15.05 ± 0.13	0.05	−0.29
		IV	14.82 ± 0.12	17.80 ± 0.18	0.000^**^	13.89–19.05	16.09 ± 0.12	0.42	−0.69
		V	15.58 ± 0.16	19.78 ± 0.22	0.000^**^	14.22–20.25	16.93 ± 0.12	0.50	−0.45
		VI	15.98 ± 0.20	20.93 ± 0.14	0.000^**^	15.41–21.64	17.72 ± 0.12	0.72	0.00
		VII	16.14 ± 0.14	21.07 ± 0.15	0.000^**^	15.65–22.84	17.75 ± 0.14	0.93	0.85
	GL	II	4.92 ± 0.04	6.25 ± 0.06	0.000^**^	2.88–7.13	4.94 ± 0.07	−0.06	−0.18
		III	6.00 ± 0.08	6.95 ± 0.05	0.000^**^	4.74–7.77	6.40 ± 0.06	−0.11	−0.12
		IV	6.15 ± 0.08	7.41 ± 0.11	0.000^**^	5.72–8.18	6.82 ± 0.05	0.20	−0.62
		V	6.32 ± 0.06	7.87 ± 0.08	0.000^**^	5.97–8.66	7.15 ± 0.05	0.39	−0.23
		VI	6.41 ± 0.10	8.60 ± 0.08	0.000^**^	5.90–9.26	7.41 ± 0.06	0.30	−0.28
		VII	6.27 ± 0.08	8.84 ± 0.11	0.000^**^	5.56–9.10	7.46 ± 0.07	0.00	−0.42
	GW	II	1.67 ± 0.02	1.49 ± 0.03	0.000^**^	1.34–2.15	1.65 ± 0.01	0.47	−0.29
		III	1.88 ± 0.03	2.32 ± 0.03	0.000^**^	1.46–2.77	1.93 ± 0.02	1.07	1.09
		IV	2.51 ± 0.03	2.94 ± 0.05	0.000^**^	1.87–3.46	2.54 ± 0.04	0.48	−0.90
		V	2.73 ± 0.05	3.49 ± 0.05	0.000^**^	2.19–3.70	2.92 ± 0.04	0.34	−1.03
		VI	3.13 ± 0.04	3.80 ± 0.04	0.000^**^	2.32–3.87	3.17 ± 0.03	0.33	−0.84
		VII	3.13 ± 0.03	3.71 ± 0.03	0.000^**^	2.34–3.94	3.22 ± 0.03	0.29	−0.89
	GD	II	2.81 ± 0.02	3.07 ± 0.03	0.000^**^	2.13–3.55	2.80 ± 0.03	0.13	−0.56
		III	3.30 ± 0.03	3.91 ± 0.05	0.000^**^	2.51–4.34	3.42 ± 0.03	0.20	−0.19
		IV	3.79 ± 0.04	4.54 ± 0.04	0.000^**^	3.26–4.80	3.99 ± 0.04	0.43	−0.97
		V	3.91 ± 0.04	4.92 ± 0.04	0.000^**^	3.58–5.34	4.32 ± 0.04	0.60	−0.59
		VI	4.29 ± 0.03	5.24 ± 0.05	0.000^**^	3.63–5.64	4.52 ± 0.04	0.78	−0.10
		VII	4.30 ± 0.04	5.37 ± 0.04	0.000^**^	3.69–5.68	4.56 ± 0.04	0.66	−0.33
2018	GFR	I	0.20 ± 0.01	0.34 ± 0.02	0.000^**^	0.09–0.43	0.20 ± 0.01	0.67	0.12
		II	0.39 ± 0.02	0.61 ± 0.02	0.000^**^	0.18–0.73	0.39 ± 0.01	0.79	0.47
		III	0.88 ± 0.04	1.52 ± 0.05	0.000^**^	0.41–2.32	0.97 ± 0.03	1.06	1.03
		IV	1.23 ± 0.04	2.36 ± 0.06	0.000^**^	0.70–2.68	1.50 ± 0.05	0.70	−0.62
		V	0.93 ± 0.02	1.90 ± 0.03	0.000^**^	0.63–2.27	1.23 ± 0.03	0.90	0.22
		VI	0.43 ± 0.02	0.87 ± 0.04	0.000^**^	0.14–1.17	0.62 ± 0.02	0.09	−0.05
		VII	0.15 ± 0.01	0.30 ± 0.02	0.000^**^	0.02–0.62	0.25 ± 0.01	0.42	−0.02
	GFR_mean_		0.60 ± 0.03	1.12 ± 0.03	0.000^**^	0.39–1.18	0.74 ± 0.02	0.57	−0.51
	GFR_max_		1.27 ± 0.03	2.51 ± 0.04	0.000^**^	0.72–3.22	1.61 ± 0.05	0.78	−0.39
	GA	II	7.23 ± 0.11	8.33 ± 0.11	0.000^**^	4.01–10.99	7.08 ± 0.12	0.20	−0.20
		III	9.55 ± 0.16	13.25 ± 0.15	0.000^**^	5.93–15.99	10.25 ± 0.18	0.40	−0.20
		IV	13.64 ± 0.21	18.64 ± 0.20	0.000^**^	8.94–20.35	13.70 ± 0.25	0.41	−0.83
		V	15.67 ± 0.18	22.69 ± 0.26	0.000^**^	11.24–23.46	16.42 ± 0.29	0.53	−0.39
		VI	17.59 ± 0.26	25.61 ± 0.30	0.000^**^	12.14–26.61	18.39 ± 0.32	0.61	−0.11
		VII	17.16 ± 0.21	25.07 ± 0.22	0.000^**^	11.42–27.16	18.56 ± 0.35	0.54	−0.29
	GP	II	12.37 ± 0.15	14.94 ± 0.12	0.000^**^	8.03–17.32	12.50 ± 0.16	−0.14	−0.32
		III	14.67 ± 0.14	16.21 ± 0.21	0.000^**^	11.61–18.95	15.63 ± 0.14	−0.09	−0.50
		IV	15.84 ± 0.16	18.66 ± 0.16	0.000^**^	13.51–20.15	16.90 ± 0.15	−0.19	−0.98
		V	16.33 ± 0.14	19.85 ± 0.22	0.000^**^	14.51–22.02	17.76 ± 0.16	−0.06	−0.86
		VI	16.54 ± 0.18	21.69 ± 0.31	0.000^**^	15.07–23.54	18.66 ± 0.18	0.19	−0.69
		VII	16.92 ± 0.16	22.68 ± 0.25	0.000^**^	15.06–23.45	18.68 ± 0.20	0.13	−1.04
	GL	II	5.26 ± 0.06	6.14 ± 0.05	0.000^**^	2.71–7.11	4.97 ± 0.08	−0.12	0.25
		III	6.31 ± 0.06	7.11 ± 0.06	0.000^**^	4.53–8.14	6.42 ± 0.07	0.00	−0.49
		IV	6.51 ± 0.08	7.60 ± 0.06	0.000^**^	5.81–8.61	6.93 ± 0.06	0.37	−0.56
		V	6.64 ± 0.07	8.26 ± 0.11	0.000^**^	6.19–8.89	7.36 ± 0.07	0.11	−0.99
		VI	6.79 ± 0.11	8.61 ± 0.08	0.000^**^	6.31–9.37	7.66 ± 0.08	0.03	−1.21
		VII	6.76 ± 0.10	8.81 ± 0.11	0.000^**^	6.02–9.50	7.55 ± 0.09	0.05	−1.21
	GW	II	1.71 ± 0.02	1.53 ± 0.02	0.000^**^	1.34–2.13	1.77 ± 0.01	−0.23	0.73
		III	1.94 ± 0.03	2.39 ± 0.03	0.000^**^	1.68–2.65	2.15 ± 0.02	0.41	0.06
		IV	2.65 ± 0.03	3.15 ± 0.04	0.000^**^	2.07–3.41	2.74 ± 0.03	0.42	−0.69
		V	3.15 ± 0.04	3.75 ± 0.04	0.000^**^	2.28–3.86	3.13 ± 0.03	0.37	−0.89
		VI	3.21 ± 0.03	3.85 ± 0.04	0.000^**^	2.32–3.96	3.30 ± 0.03	0.11	−0.50
		VII	3.31 ± 0.04	3.86 ± 0.04	0.000^**^	2.51–4.19	3.33 ± 0.03	0.25	−0.56
	GD	II	2.87 ± 0.02	3.21 ± 0.04	0.002^**^	2.20–3.91	2.97 ± 0.03	0.36	−0.08
		III	3.42 ± 0.03	4.26 ± 0.04	0.000^**^	2.81–4.52	3.66 ± 0.03	−0.08	−0.16
		IV	3.97 ± 0.03	4.87 ± 0.03	0.000^**^	3.36–5.05	4.23 ± 0.04	0.02	−0.97
		V	4.21 ± 0.04	5.29 ± 0.05	0.000^**^	3.67–5.43	4.52 ± 0.04	0.34	−0.59
		VI	4.40 ± 0.04	5.61 ± 0.06	0.000^**^	3.62–5.81	4.72 ± 0.04	0.31	−0.36
		VII	4.51 ± 0.05	5.60 ± 0.04	0.000^**^	3.72–5.86	4.81 ± 0.05	0.27	−0.57

^a^GFR, grain filling rate; GFR_mean_, mean grain filling rate; GFR_max_, maximum grain filling rate; GA, grain area; GP, grain perimeter; GL, grain length; GW, grain width; GD, grain diameter. ^b^I, II, III, IV, V, VI and VII represent 7, 14, 21, 28, 35, 42 and 49 days after flowering, respectively. ^c^ST: Significant; ^**^Significant at 0.01 level.

**Figure 2:**
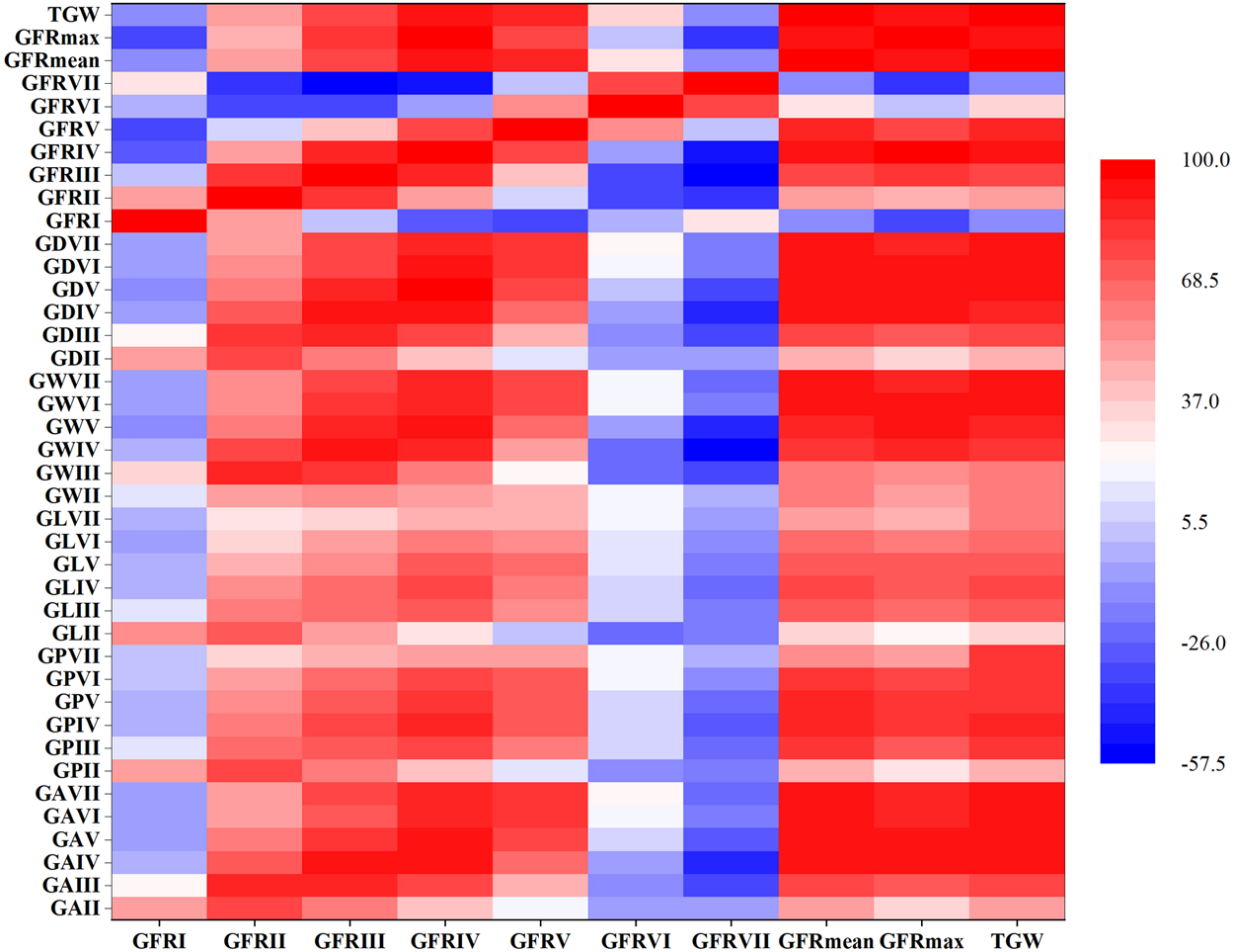
Correlation coefficients among grain filling rate, grain size and thousand grain weight averaged across two years.

The Pearson correlation coefficients (r) between GA, GP, GL, GW, GD, TGW, GFR_mean_, GFR_max_ and GFR traits at seven sampling stages over two years were given in Fig. 2. Grain size traits were significantly correlated with each other at each stage (not listed), and grain size traits at all stages were significantly positively correlated with GFR at stage II, III, IV and V, GFR_mean_, GFR_max_ and TGW, whereas GFR at stages I, VI and VII were mostly unrelated or negatively correlated with grain size traits at each stage. TGW showed high positive correlation coefficients with GFR_mean_ and GFR_max_ (r > 0.9), while GFR at stage III, IV and V were also positive correlation coefficients with TGW (r > 0.7). The initial and final stages of GFR was not correlated with GFR_mean_ and TGW, but negatively with GFR_max_ (Fig. 2).

### Unconditional QTL analysis

**Figure 3:**
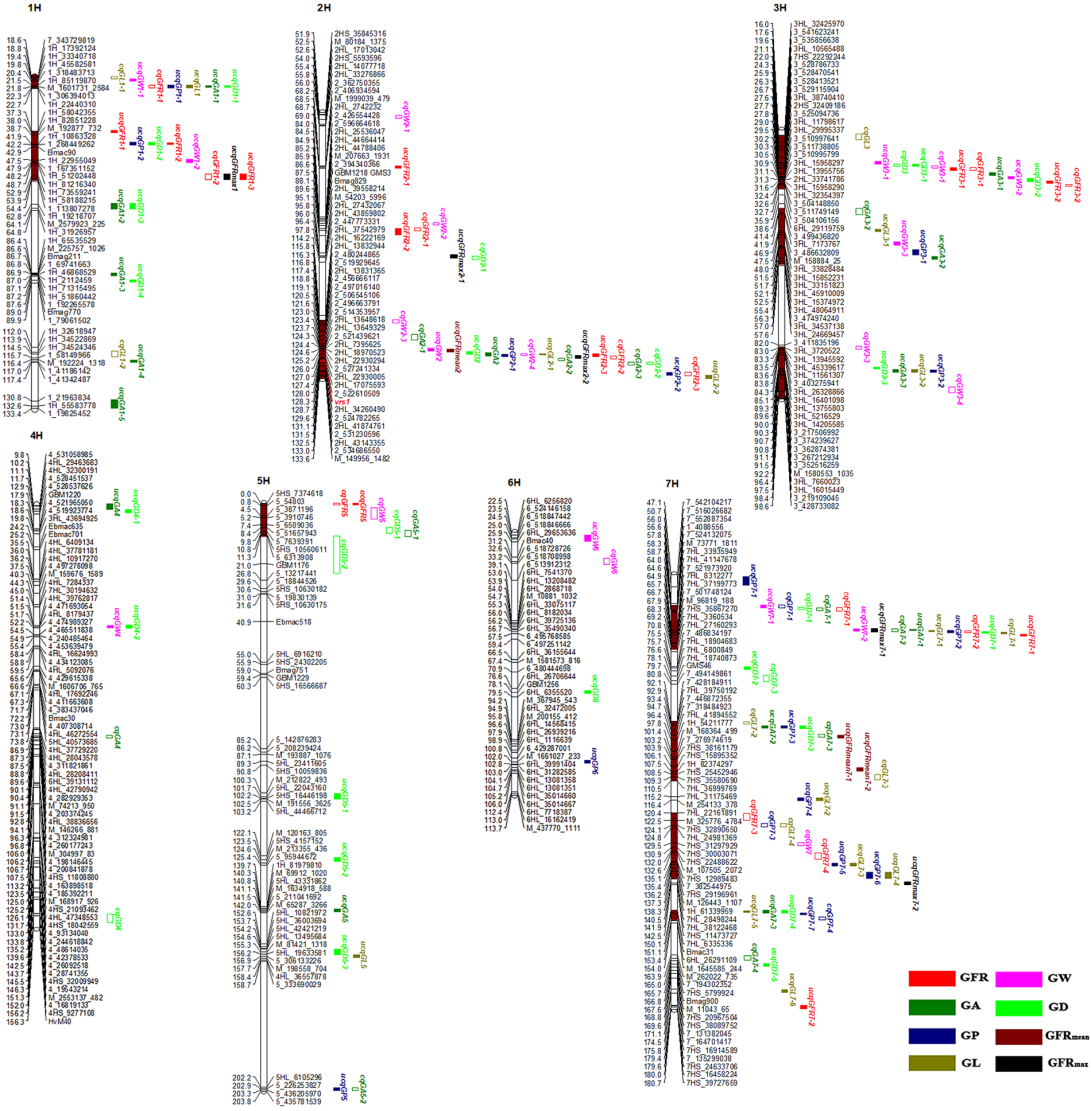
Chromosomes location of the consensus QTLs associated with GFR, GFR_mean_, GFR_max_ and five grain size traits. The marker names are listed on the right of the linkage groups, and the positions on the left, given in centimorgan (cM). Solid and hollow bars represent the QTLs that were mapped using unconditional and conditional methods, respectively, the brown area on the linkage groups represents the QTL cluster. Abbreviations are shown in the footnote of Table 1.

A total of 204 unconditionally identified QTLs for GFR_mean_, GFR_max_, GA, GP, GL, GW, GD and GFR were detected on 7 chromosomes, individually accounted for 1.12–78.03% phenotypic variation (Supplementary Table S2). Up to 152 identified QTLs with overlapping CIs were detected and integrated into 38 consensus QTLs. The other 52 non-overlapping identified QTLs were also considered to be consensus QTLs, of which 8 QTLs for GFR_mean_ and GFR_max_ were regarded as the unconditional consensus QTLs (Table 2, Supplementary Table S2). In total, 90 unconditional consensus QTLs were detected (Fig. 3, Table 2).

**Table 2:** Unconditional consensus QTLs underlying the grain filling rate and grain size traits at different grain filling stages.

Trait	Consensus QTLs	Chr.^a^	Peak	The closest marker	CI^b^	LOD	R2c	Add^d^	Stage^e^
GFR	ucqGFR1–1	1H	37.79	1H_82851228	37.35–38.24	33.24–34.12	16.33–19.47	0.10–0.11	Y1.II/Y2.II
	ucqGFR1–2	1H	42	1H_10863328	41.71–42.28	7.03–47.17	7.12–36.22	−0.18 to −0.12	Y2.II,III
	ucqGFR1–3	1H	54	1H_58188215	52.4–54.5	6.81	4.40	−0.11	Y2.IV
	ucqGFR2–1	2H	68.5	M_1999039_479	68.28–68.87	3.10–4.12	2.69–2.83	−0.11 to 0.09	Y1.IV/Y2.III
	ucqGFR2–2	2H	88	Bmag829	87.5–89.5	4.85	12.20	−0.05	Y2.VII
	ucqGFR2–3	2H	126.25	2HL_22930005	125.75–127.5	15.57–46.56	33.71–71.54	0.08–0.51	Y1.II,III,IV,V/Y2.III,IV,V
	ucqGFR3–1	3H	28	3_525094736	27.64–28.35	6.04–8.81	9.66–14.26	0.13–0.14	Y1.V/Y2.V
	ucqGFR3–2	3H	32	3HL_15958290	31.71–32.28	4.39–7.32	4.76–24.12	−0.10 to 0.11	Y1.II,III,VI/Y2.III,VI,VII
	ucqGFR5	5H	0	5HS_7374618	0–0.5	4.12–4.39	5.76–6.24	−0.09 to −0.08	Y1.V/Y2.V
	ucqGFR7–1	7H	66.5	7_501748124	66.25–66.75	5.35–12.69	7.66–9.96	0.10–0.17	Y1.IV,V/Y2.V
	ucqGFR7–2	7H	180	7HS_16458224	179.5–180.5	4.74–8.18	7.63–8.07	0.03–0.10	Y1.II,III
GA	ucqGA1–1	1H	22	1_306394013	21.64–22.35	3.23–3.57	7.65–8.64	−0.41 to −0.38	Y1.II/Y2.II
	ucqGA1–2	1H	64	M_2579923_225	62.5–64.5	16.69	7.44	0.81	Y1.IV
	ucqGA1–3	1H	87	1H_2112459	86.5–87.5	26.95	15.06	−1.15	Y1.IV
	ucqGA1–4	1H	117	1_41186142	116.5–117.5	5.49	3.32	−0.48	Y2.IV
	ucqGA1–5	1H	133	1_19825452	130.5–133.5	3.92–4.60	1.29–4.31	−0.50 to −0.39	Y2.VI,VII
	ucqGA2	2H	126.13	2_527241334	125.55–126.64	9.51–55.19	25.91–75.68	0.76–2.85	Y1.II,III,IV,V,VI,VII
**Y2.II,III,IV,V,VII**									
	ucqGA3–1	3H	30	3_510997641	29.5–30.5	4.39	1.54	0.46	Y2.VII
	ucqGA3–2	3H	57	3HL_34537138	56.5–57.5	5.13	1.70	0.45	Y2.VII
	ucqGA3–3	3H	91	3_267212934	90.5–91.5	5.03	4.85	−0.53	Y2.VI
	ucqGA4	4H	10	4HL_29463683	9.5–11.5	3.931	1.74	−0.37	Y1.V
	ucqGA5	5H	141	M_1634918_588	140.5–141.5	3.19	1.43	−0.35	Y1.V
	ucqGA7–1	7H	65	7HL_8312277	64.71–65.28	12.66–35.07	9.43–30.11	0.8–1.6	Y2.IV,VI,VII
	ucqGA7–2	7H	94	Bmag746	93.64–94.35	13.42–13.47	10.99–11.34	1.07–1.10	Y1.VI,VII
	ucqGA7–3	7H	151	Bmac31	150.5–151.5	15.61	6.44	0.87	Y2.VII
GP	ucqGP1–1	1H	22	1_306394013	21.5–22.5	4.04	10.26	−0.54	Y1.II
	ucqGP1–2	1H	42	1H_10863328	41.5–42.5	3.62	7.82	−0.51	Y2.II
	ucqGP2–1	2H	126.25	2_527241334	125.93–126.54	8.21–37.66	15.96–62.17	0.52–1.21	Y1.II,III,IV,V,VI,VII
**Y2.II,III,IV,VI,VII**									
	ucqGP2–2	2H	132	2HL_43143355	131.5–132.5	25.32	24.71	0.90	Y2.V
	ucqGP3–1	3H	55	3HL_48064911	54.13–55.86	3.39–9.21	3.25–10.44	0.24–0.39	Y1.V,VI/Y2.IV
	ucqGP3–2	3H	91	3_267212934	90.64–91.35	6.36–11.91	5.14–13.83	−0.45 to −0.30	Y1.V,VI
	ucqGP5	5H	203	5_226253827	202.5–203.5	4.76	4.78	−0.26	Y1.VI
	ucqGP6	6H	102	M_1661027_233	101.5–102.5	30.3724	31.46	0.94	Y2.V
	ucqGP7–1	7H	49	GBM1102	47.5–50.5	5.26	5.45	0.31	Y2.III
	ucqGP7–2	7H	65.5	7HL_37199773	65.25–65.75	11.84–31.98	13.73–44.71	0.49–1.23	Y2.IV,V,VI,VII
	ucqGP7–3	7H	94	Bmag746	93.64–94.35	9.36–14.66	9.99–13.89	0.42–0.69	Y2.III,VII
	ucqGP7–4	7H	116	5_496886371	115.5–116.5	17.12	21.04	0.61	Y2.III
	ucqGP7–5	7H	136	7HS_29196961	135.5–136.5	27.36	26.39	−1.04	Y2.VI
	ucqGP7–6	7H	140	7_319506952	138.5–140.5	21.31	30.24	0.77	Y1.III
	ucqGP7–7	7H	151.77	2_287569753	151.44–152.11	7.18–22.28	16.00–30.90	0.52–0.75	Y1.VI/Y2.II,IV
GL	ucqGL1	1H	22	1_306394013	21.5–22.5	5.33	12.70	−0.29	Y1.II
	ucqGL2–1	2H	125.94	2_527241334	125.76–126.11	12.44–29.66	15.52–51.03	0.35–0.45	Y1.III,IV,V,VI,VII
**Y2. V,VI,VII**									
	ucqGL2–2	2H	133.1	2_534686550	132.62–133.57	5.39–5.79	12.84–14.43	0.31–0.34	Y1.II/Y2.II
	ucqGL3–1	3H	48	3HL_33828484	47.18–47.87	4.35–5.53	3.96–7.23	0.12–0.13	Y1.V/Y2.IV
	ucqGL3–2	3H	91	3_267212934	90.5–91.5	6.59	6.36	−0.15	Y1.V
	ucqGL5	5H	157	5_306133226	156.5–157.5	3.25	2.94	−0.11	Y1.V
	ucqGL7–1	7H	65.25	7HL_13143105	65.0–65.5	15.66–50.97	25.01–45.19	0.25–0.61	Y2.IV,V,VI,VII
	ucqGL7–2	7H	116	5_496886371	115.5–116.5	10.26	21.79	0.27	Y2.III
	ucqGL7–3	7H	136	7HS_29196961	135.64–136.35	28.14–38.74	23.52–27.20	−0.48 to −0.37	Y2.VI,VII
	ucqGL7–4	7H	140	7_319506952	138.5–140.5	22.26	37.83	0.40	Y1.III
	ucqGL7–5	7H	150.92	Bmac31	150.64–151.2	6.67–24.79	17.10–43.21	0.35–0.50	Y1.V,VI,VII/Y2.II
	ucqGL7–6	7H	175	7_144173681	174.5–175.5	3.20	7.47	0.22	Y1.II
GW	ucqGW1–1	1H	19.76	1H_45582581	19.42–20.09	5.08–7.31	2.71–5.45	−0.09 to −0.06	Y1.IV,V,/Y2.V,VII
	ucqGW1–2	1H	48	1_167351152	47.5–48.5	6.47	3.25	−0.06	Y2.VI
	ucqGW2	2H	124.69	2HL_18970523	124.44–125.25	8.21–53.91	32.27–75.51	0.09–0.39	Y1.II,III,IV,V,VI,VII
**Y2.II,III,IV,V,VI,VII**									
	ucqGW3–1	3H	26	3_528470541	25.5–26.5	7.01	3.06	0.07	Y1.VI
	ucqGW3–2	3H	31.33	2HL_33741786	31.04–31.62	3.62–4.75	2.16–6.75	−0.07 to 0.06	Y1.III/Y2.VI,VII
	ucqGW3–3	3H	52	3HL_45910009	51.5–52.5	3.53	1.43	−0.05	Y1.VI
	ucqGW4	4H	52	4_474989327	51.64–52.35	4.09–4.48	1.18–2.32	−0.05	Y2.IV,VII
	ucqGW6	6H	25	6_518846666	23.5–25.5	3.79	1.63	−0.05	Y1.VII
	ucqGW7–1	7H	57	1_4088556	56.5–57.5	5.82	4.28	0.08	Y1.IV
	ucqGW7–2	7H	65.13	7HL_13143105	64.78–65.66	7.63–46.68	5.61–34.11	−0.18 to 0.26	Y1.IV,V,VII/Y2.IV,V,VI
GD	ucqGD1–1	1H	22	1_306394013	21.64–22.35	3.94–4.05	8.34–9.90	−0.11 to −0.10	Y1.II/Y2,II
	ucqGD1–2	1H	42	1H_10863328	41.5–42.5	4.14	5.92	−0.09	Y2.III
	ucqGD1–3	1H	64	M_2579923_225	62.5–64.5	16.54	7.09	0.12	Y1,IV
	ucqGD1–4	1H	89.17	Bmag770	88.89–89.46	14.26–27.03	6.30–14.59	−0.18 to 0.12	Y1.IV,V
	ucqGD2	2H	125.93	2_527241334	125.69–126.44	8.35–56.41	18.90–78.30	0.18–0.42	Y1.II,III,IV,V,VI,VII
**Y2. II,III,IV,V,VI,VII**									
	ucqGD3–1	3H	27	3_529115904	26.5–27.5	4.19	1.96	0.06	Y1.VI
	ucqGD3–2	3H	32	3HL_15958290	31.5–32.5	9.02	2.93	0.09	Y2.VII
	ucqGD3–3	3H	90	3HL_14205585	89.64–90.35	4.49–4.78	2.29–2.46	−0.07 to −0.06	Y1.VI,VII
	ucqGD4–1	4H	12	4_528451537	11.5–12.5	4.22	1.22	−0.05	Y2.V
	ucqGD4–2	4H	52	4_474989327	51.5–52.5	4.59	1.39	−0.05	Y2.VII
	ucqGD5–1	5H	102	5HS_16446198	100.5–102.5	11.71	3.87	0.10	Y2.V
	ucqGD5–2	5H	123.5	5HS_4157152	122.79–124.2	4.05–21.31	1.12–8.72	−0.14 to −0.05	Y2.V,VII
	ucqGD5–3	5H	155	M_81421_1318	154.5–156.5	5.41	2.71	−0.07	Y1.VI
	ucqGD6	6H	78	GBM1256	77.5–78.5	5.54	1.66	0.06	Y2.V
	ucqGD7–1	7H	65.88	7HL_37199773	65.63–66.12	13.71–44.92	10.08–31.65	0.13–0.26	Y1.VI/Y2.IV,V,VI,VII
	ucqGD7–2	7H	76	7_500661278	75.5–76.5	17.85	31.06	0.21	Y2.III
	ucqGD7–3	7H	94	Bmag746	93.5–94.5	10.79	3.43	0.09	Y2.VII
	ucqGD7–4	7H	151	Bmac31	150.5–151.5	9.59	6.33	0.11	Y2.VI
	ucqGD7–5	7H	167	Bmag900	166.5–167.5	5.46	3.04	0.08	Y1.VII
GFRmax	ucqGFRmax1	1H	54	1H_58188215	52.5–54.5	5.85	4.11	−0.11	Y2
	ucqGFRmax2–1	2H	96	2HL_43859802	95.5–96.5	3.58	2.44	0.09	Y1
	ucqGFRmax2–2	2H	127	2HL_22930005	126.5–127.5	43.38–43.39	68.26–70.11	0.5–0.54	Y1/Y2
	ucqGFRmax7–1	7H	65	7HL_8312277	64.5–65.5	13.51	10.99	0.19	Y1
	ucqGFRmax7–2	7H	142	7HL_38122468	141.5–142.5	13.53	11.38	0.19	Y2
GFRmean	ucqGFRmean2–1	2H	125	2HL_22930294	124.5–125.5	49.39–56.94	69.98–74.83	0.17–0.18	Y1/Y2
	ucqGFRmean7–1	7H	97	7_440111505	96.5–97.5	13.31	9.23	0.06	Y1
	ucqGFRmean7–2	7H	107	7_266425095	106.5–107.5	21.68	11.69	0.06	Y2

^a^Chromosome. ^b^The 1.5-LOD confidence interval of QTLs. ^c^The phenotypic variance explained by each QTL. ^d^Additive effect. ^e^Y1 and Y2 represent 2017 and 2018, respectively. Abbreviations are shown in the footnote of Table 1.

For the GFR_max_ and GFR_mean_, eight QTLs were detected on chromosome 1H, 2H, and 7H over two years, individually explaining 2.44–74.83% of PVE (Table 2). Among these QTLs, two main-effect QTLs were detected on 2H, *ucqGFR*_*mean*_2 was located at 125 cM close to the marker 2HL_22930294, and *ucqGFR*_*max*_*2–2* was located at 127 cM near the marker 2HL_22930005. These alleles from Huadamai6 contributed PVE 68.26–74.83%.

In total, 30 identified QTLs located on 7 chromosomes except 4H and 6H were detected for GFR. Of those, 28 overlapping QTLs were integrated into 9 unconditional consensus QTLs and 2 non-overlapping QTLs also regarded as consensus QTLs (Table 2, Supplementary Table S2). Among these consensus QTLs, 4 QTLs were detected repeatedly at the same sampling stage over two years. *ucqGFR1-2* located on 1H, linked to the SNP marker 1H_10863328 of 42 cM, was expressed at stage II and III in Y2, explaining 7.12–36.22% of PVE. *ucqGFR3-2* linked to the marker 3HL_15958290 of 32 cM was detected at stage II, III and VI in Y1 and at stage III, VI and VII in Y2 with PVE ranging from 4.76 to 24.12%. The QTL *ucqGFR3-3* showed negative additive effects and reduced the GFR from the Huadamai6 alleles. *ucqGFR2-3* closely linked to the marker 2HL_22930005 of 127 cM was expressed simultaneously at stage II, III, IV and V in Y1 and at stage III, VI and V in Y2, accounting for 33.71–71.54% PVE, with the allele increasing GFR contributed by Huadamai6.

A total of 29 identified QTLs on 7 chromosomes were detected for GA. Among these QTLs, 20 overlapping QTLs were integrated into 5 consensus QTLs (Table 2, Supplementary Table S2). Only one QTL (*ucqGA2*) tightly linked to 2_527241334 of 126.13 cM was consistently expressed at all stages in Y1 and at stage II, III, IV, V and VII in Y2, explaining 25.91–75.68% of PVE, with additive effects varying from 0.76 to 2.68; another QTL *ucqGA7-1* was detected simultaneously at three different stages in Y2, explaining 12.66–35.07% of PVE. Additionally, two QTLs were detected at two different stages in particular year, and *ucqGA1-1* was detected at stage II in both years. The remaining 9 consensus QTLs were only identified at one stage in a particular year.

For the GP, 35 identified QTLs located across 7 chromosomes except 4H were detected over two years. Of these, 26 overlapping QTLs and 9 non-overlapping QTLs were integrated into 15 consensus QTLs (Table 2, Supplementary Table S2). Among these consensus QTLs, *ucqGP2-1* was 126.25 cM from 2_527241334 and consistently detected at all stages in Y1 and at stage II, III, IV, VI and VII in Y2, which individually explained 15.96–62.17% of PVE. *ucqGP7-2* tightly linked to the 7HL_37199773 of 65.5 cM, was expressed at four different stages in Y2, and contributed 13.73–44.71% PVE. In addition, *ucqGP7-7* closely linked to the 2_287569753 at a genetic distance of 151.77 cM was detected at stage VI in Y1 and at stage II and IV in Y2, individually explaining up to 16–30.9% of PVE. These alleles increasing GP were from Huadamai6. *ucqGP3-1* was detected consistently at stage V and VI in Y1 and at stage IV in Y2. Another two QTLs (*ucqGP3-2* and *ucqGP7-3*) were detected at two different stages in particular year.

A total of 30 identified QTLs on 5 chromosomes (excluding 4H and 6H) were detected for GL. Of these QTLs, integrating 24 QTLs with overlapping CIs into 6 consensus QTLs and 6 non-overlapping QTLs were also considered to be consensus QTLs (Table 2, Supplementary Table S2). At stages III-VII, the major QTL *ucqGL2-1* was 125.94 cM from 2_527241334 and explained up to 15.52–51.03% of PVE. At stages IV-VII in Y2, the main-effect *ucqGL7-1* was closely linked to the marker 7HL_13143105 of 65.25 cM, accounting for 25.01–45.19% PVE. *ucqGL7-3* tightly linked to the 7HS_29196961 was detected at stage VI and VII in Y2, explaining 23.52–27.20% of PVE. *ucqGL7-5* closely linked to the marker Bmac31 of 150.92 cM was expressed at stages V-VII in Y1 and at stage II in Y2, explaining 17.10–43.21% of PVE. In addition, two QTLs *ucqGL2-2* and *ucqGL3-1* were detected at one or two stages in both years.

For the GW, 33 identified QTLs distributed on 7 chromosomes except 5H were detected over two years. Of which, 28 QTLs with overlapping CIs and 5 non-overlapping QTLs were integrated into 10 consensus QTLs (Table 2, Supplementary Table S2). *ucqGW1-1*, and *ucqGW3-2* were expressed at one or two stages across two years. *ucqGW4* was detected at stage IV and VII in Y2 and exhibited negative additive effects. In addition, *ucqGW7-2* was 65.13 cM from 7HL_13143105 and expressed at stage IV, V and VII in Y1 and at stages IV-VI in Y2, explaining 7.63-46.68 of PVE. *ucqGW3-2* and *ucqGW7-2* expressed additive effects in opposite directions at different stages. Only *ucqGW2* closely linked to the 2HL_22930294 of 124.69 cM was identified steadily at all stages in both years, explaining up to 32.27–75.51% of PVE.

For the GD, 39 identified QTLs located on 7 chromosomes were detected across two years. Of which, 26 overlapping were integrated into 6 consensus QTLs, the other 13 non-overlapping QTLs were also considered as consensus QTLs (Table 2, Supplementary Table S2). *ucqGD1-1*, *ucqGD3-3* and *ucqGD5-2* were detected at one or two stages over two years with −0.14 to −0.05 additive effects. The major QTL *ucqGD2* closely linked to the 2_527241334 of 125.93 cM was stably expressed at all stages in both years, accounting for 18.90-78.03% PVE. In addition, *ucqGD7-1* tightly linked to the 7HL_37199773 of 65.88 cM was expressed at stage VI in Y1 and at stages IV-VII in Y2, accounting for 10.08–31.65% PVE.

### Conditional QTL analysis

In total, 95 conditionally identified QTLs were detected for GA, GP, GL, GW, GD and GFR across two years, and individually explained 1.26–65.72% of PVE (Supplementary Table S3). Among these QTLs, 55 conditional consensus QTLs consisted of 22 consensus QTLs integrated from 62 overlapping identified QTLs and 33 non-overlapping identified QTLs (Fig. 3, Table 3).

**Table 3:** Conditional consensus QTLs underlying the grain filling rate and grain size traits at different grain filling periods.

Trait	Consensus QTLs	Chr^a^	Peak	The closest marker	CI^b^	LOD	R2c	Add^d^	Stage^e^
GFR	cqGFR1–1	1H	22	M_1601731_2584	21.5–22.5	3.22	3.99	−0.02	Y1.ΔT2
	cqGFR1–2	1H	54	1H_58188215	52.5–54.5	3.27–4.97	1.54–5.54	−0.02 to 0.08	Y2.ΔT2,ΔT5
	cqGFR2–1	2H	88	Bmag829	87.35–88.24	3.74–4.69	1.26–12.78	−0.12 to 0.05	Y1.ΔT3,ΔT5, Y2.ΔT3,ΔT5
	cqGFR2–2	2H	126.83	2HL_22930005	126.45–127.25	6.51–39.08	14.92–65.72	−0.28 to 0.23	Y1.ΔT2,ΔT3,ΔT4,ΔT6,ΔT7
**Y2.ΔT2,ΔT3, ΔT4,ΔT5,ΔT6,ΔT7**									
	cqGFR2–3	2H	132	2HL_43143355	131.5–132.5	8.69	26.63	−0.19	Y1.ΔT5
	cqGFR3–1	3H	28	3_525094736	27.64–28.35	4.88–6.94	12.25–14.46	−0.06 to 0.10	Y1.ΔT7/Y2.ΔT4
	cqGFR3–2	3H	33	3_511749149	32.66–33.33	4.06–9.63	1.40–22.86	−0.07 to 0.16	Y1.ΔT3,ΔT5/Y2. ΔT2,ΔT3,ΔT5,ΔT7
	cqGFR5	5H	0.03	5HS_7374618	0.21–0.28	3.19–3.68	5.17–5.35	−0.07 to −0.06	Y1.ΔT4/Y2.ΔT4
	cqGFR7–1	7H	58	1_4089724	57.5–58.5	5.06–5.52	7.49–9.44	0.08–0.09	Y1.ΔT4/Y2.ΔT4
	cqGFR7–2	7H	65.5	7HL_37199773	65.25–65.75	3.77–5.75	8.46–11.91	−0.09 to −0.04	Y1.ΔT6,ΔT7/Y2.ΔT7
	cqGFR7–3	7H	122	7HS_21726812	120.5–122.5	6.25	8.52	−0.08	Y1.ΔT6
	cqGFR7–4	7H	133	7HS_17906516	132.5–134.5	8.66	16.27	−0.11	Y1.ΔT7
GA	cqGA2–1	2H	121	2_506545106	120.5–122.5	4.00	8.35	0.30	Y1.ΔT5
	cqGA2–2	2H	127.54	2HL_17075593	127.13–127.84	9.25–15.63	27.71–40.97	0.77–0.88	Y1.ΔT4/Y2.ΔT3,ΔT4
	cqGA3–1	3H	33.3	3_511749149	32.47–34.13	3.12–4.22	6.79–9.36	0.23–0.35	Y1.ΔT6/Y2.ΔT6
	cqGA3–2	3H	41	3_499436820	39.93–42.06	5.06–5.14	10.48–12.13	0.32–0.38	Y1.ΔT5/Y2.ΔT5
	cqGA4	4H	90	4HL_42790942	89.5–90.5	3.48	7.42	−0.23	Y2.ΔT6
	cqGA5–1	5H	11	5HS_10560611	9.5–11.5	4.79	9.98	−0.30	Y1.ΔT5
	cqGA5–2	5H	203	5_226253827	202.5–203.5	3.74	8.59	−0.31	Y2.ΔT5
	cqGA7–1	7H	58	1_4089724	57.5–58.5	4.78	11.32	0.46	Y2.ΔT4
	cqGA7–2	7H	65	7HL_8312277	64.5–65.5	7.85	27.58	0.22	Y2.ΔT7
	cqGA7–3	7H	97	7_440111505	96.5–97.5	7.54	18.97	0.46	Y2.ΔT5
	cqGA7–4	7H	165	7_194302352	164.29–165.7	3.22–4.48	11.45–12.31	0.42–0.52	Y1.ΔT3/Y2.ΔT3
GP	cqGP7–1	7H	57	1_4088556	56.5–57.5	3.50	14.30	0.52	Y2.ΔT3
	cqGP7–2	7H	68.99	7HL_3360534	68.64–69.35	5.01–13.78	17.05–41.39	0.20–0.52	Y2.ΔT6,ΔT7
	cqGP7–3	7H	124	7HS_32890650	123.5–124.5	25.21	16.88	1.57	Y1.ΔT3
	cqGP7–4	7H	153	M_90598_706	152.5–153.5	18.28	10.41	−1.22	Y1.ΔT3
GL	cqGL1–1	1H	19	1H_17392124	18.64–19.35	3.02–8.28	11.34–14.00	0.21–0.32	Y1.ΔT3/Y2.ΔT3
	cqGL1–2	1H	114	1H_34522869	113.5–115.5	5.68	9.06	−0.26	Y2.ΔT3
	cqGL3	3H	19	3HL_42780152	17.5–19.5	4.61	7.29	0.24	Y2.ΔT3
	cqGL7–1	7H	66	7HL_4313756	65.71–66.28	4.22–8.07	9.08–27.07	0.09–0.26	Y1.ΔT6/Y2.ΔT3,ΔT5
	cqGL7–2	7H	92.5	7HL_27996637	92.14–92.85	4.37–12.36	12.70–38.03	0.06–0.11	Y1.ΔT7/Y2.ΔT7
	cqGL7–3	7H	109	7HS_35580690	108.5–110.5	3.22	12.19	−0.11	Y2.ΔT4
	cqGL7–4	7H	124	7HS_32890650	123.5–124.5	3.20	12.31	0.22	Y1.ΔT3
GW	cqGW2–1	2H	53	2HL_17013042	52.5–53.5	4.08	15.00	−0.06	Y1.ΔT6
	cqGW2–2	2H	86	M_207663_1931	85.5–86.5	3.82	12.43	−0.05	Y2.ΔT6
	cqGW2–3	2H	116	2HL_13832944	115.5–116.5	5.00	18.33	0.06	Y2.ΔT5
	cqGW2–4	2H	126	2_527241334	125.75–126.25	4.29–23.87	13.33–55.68	0.05–0.16	Y1.ΔT3,ΔT4/Y2.ΔT3,ΔT4
	cqGW3–1	3H	27.5	3HL_38740410	27.14–27.85	3.09–4.08	9.85–13.72	−0.04 to 0.06	Y1.ΔT5/Y2.ΔT3
	cqGW3–2	3H	32.19	3HL_32354397	31.75–32.64	4.74–6.31	16.11–20.59	−0.09 to 0.06	Y1.ΔT3/Y2.ΔT6
	cqGW3–3	3H	83	3HL_3720522	82.5–83.5	3.07	10.93	−0.05	Y1.ΔT6
	cqGW3–4	3H	98	3_219109045	96.5–98.5	4.17	15.39	−0.05	Y2.ΔT5
	cqGW5	5H	4	5_3871196	1.5–5.5	3.71	11.83	−0.05	Y1.ΔT5
	cqGW6	6H	32	6_518728726	31.5–33.5	3.22	11.98	−0.04	Y2.ΔT7
	cqGW7	7H	130	7HS_25905506	129.5–130.5	3.93	7.76	0.07	Y1.ΔT4
GD	cqGD2–1	2H	96	2HL_43859802	95.5–96.5	4.24	13.74	0.11	Y1.ΔT3
	cqGD2–2	2H	129	2HL_34260490	128.64–129.35	6.05–6.11	18.55–20.41	0.10–0.11	Y1.ΔT4/Y2.ΔT4
	cqGD3	3H	27	3_529115904	26.5–27.5	3.71	10.91	0.05	Y1.ΔT5
	cqGD4	4H	152	4_16819133	151.5–154.5	3.31	9.05	−0.04	Y1.ΔT7
	cqGD5–1	5H	9	5_51657943	8.5–10.5	3.04	9.30	0.04	Y1.ΔT7
	cqGD5–2	5H	17	GBM1176	11.5–24.5	3.08	9.62	−0.04	Y1.ΔT5
	cqGD7–1	7H	57.6	7_523855164	57.15–58.04	3.14–3.36	10.74–12.89	0.09	Y1.ΔT3/Y2.ΔT3
	cqGD7–2	7H	70	M_363857_407	69.5–70.5	10.99	34.69	0.09	Y2.ΔT7
	cqGD7–3	7H	80	GMS46	78.5–80.5	6.18	17.99	0.06	Y1.ΔT7

^a^Chromosome. ^b^The 1.5-LOD confidence interval of QTLs. ^c^The phenotypic variance explained by each QTL. ^d^Additive effect. ^e^Y1 and Y2 represent 2017 and 2018, respectively, ΔT2, ΔT3, ΔT4, ΔT5, ΔT6 and ΔT7 represent the time intervals I-II, II-III, III-IV, IV-V, V-VI and VI-VII, respectively. Abbreviations are shown in the footnote of Table 1.

For the GFR, 36 identified QTLs distributed on 7 chromosomes except 6H and 4H were detected in both years. The 36 identified QTLs were integrated into 12 consensus QTLs, including 4 non-overlapping identified QTLs (Table 3, Supplementary Table S3). The main-effect QTL *cqGFR2-2* was stably expressed at ΔT2, ΔT3, ΔT4, ΔT6 and ΔT7 in Y1 and at all intervals in Y2, explaining 14.92–65.72% of PVE and the additive effects had opposite directions in different intervals. Another major QTL *cqGFR3-2* was detected at ΔT3 and ΔT5 in Y1, and ΔT2, ΔT3, ΔT5 and ΔT7 in Y2, accounting for 1.40–22.86% PVE with additive effects ranging from −0.07 to 0.16. Similarly, *cqGFR2-1* and *cqGFR3-1* had opposite additive effects at different time intervals.

Sixteen identified QTLs, including three QTLs each at ΔT3, ΔT4 and ΔT6, six at ΔT5 and one at ΔT7 were detected for the GA. Nine overlapping and 7 non-overlapping QTLs were integrated into 11 conditional consensus QTLs (Table 3, Supplementary Table S3). *cqGA3-1*, *cqGA3-2* and *cqGA7-4* were repeatedly detected at a specific time interval in both years, and the alleles increasing GA were contributed by Huadamai6. Another QTL *cqGA2-2* closely linked to the 2HL_17075593 of 127.54 cM was consistently expressed at ΔT4 in Y1 and at ΔT3 and ΔT4 in Y2, explaining 27.71–40.97% of PVE. The remaining 7 conditional consensus QTLs were only detected at a single interval in a particular year.

For the GP, five identified QTLs consisting of three QTLs at ΔT3 and one each at ΔT6 and ΔT7 were detected on 7H. The five identified QTLs were integrated into 4 conditional consensus QTLs (Table 3, Supplementary Table S3). Only *cqGP7-2* tightly linked to the 7HL_3360534 of 68.99 cM was expressed simultaneously at two intervals (ΔT6 and ΔT7) in Y2, with explaining 17.05–41.39% of PVE. Among these consensus QTLs, three of them increased GP contributing from the Huadamai6 allele, and one from Huaai11.

In total, 11 identified QTLs, including six QTLs at ΔT3, one each at ΔT4, ΔT5 and ΔT6 and two at ΔT7 detected for the GL were integrated into 7 conditional consensus QTLs, including 4 non-overlapping identified QTLs (Table 3, Supplementary Table S3). *cqGL1-1* and *cqGL7-2* were repeatedly detected at specific intervals over two years, with PVE of 11.34-14.00% and 12.70–38.03%, respectively. In addition, *cqGL7-1* was consistently expressed at ΔT6 inY1 and at ΔT3 and ΔT5 in Y2, explaining 9.08–27.07% of PVE.

For the GW, 16 identified QTLs were detected, including four each at ΔT3, ΔT5 and ΔT6, three at ΔT4 and one at ΔT7. Eight identified QTLs with overlapping CIs and 8 non-overlapping identified QTLs were integrated into 11 conditional consensus QTLs (Table 3, Supplementary Table S3). *cqGW2-4* was expressed at ΔT3 and ΔT4 in both years, explaining 13.33–55.68% of PVE. Another major QTL *cqGW3-2* closely linked to the 3HL_32354397 of 32.19 cM was repeatedly detected at ΔT3 in Y1 and at ΔT6 in Y2, and explained 16.11–20.59% of PVE. However, the additive effects of *cqGW3-2* at different intervals were opposite. In addition, *cqGW3-1* was detected at ΔT5 in Y1 and at ΔT3 in Y2, with opposite additive effects. The remaining 8 consensus QTLs were detected at a single interval in particular year, 6 of them increased the GW were from Huaai11 alleles.

For the GD, 3, 2, 2 and 4 identified QTLs were detected at ΔT3, ΔT4, ΔT5 and ΔT7, respectively. The 11 identified QTLs were integrated into 9 conditional consensus QTLs, the 7 non-overlapping identified QTLs were also regarded as consensus QTLs (Table 3, Supplementary Table S3). *cqGD2-2* and *cqGD7-1* were stably detected at the same interval over two years. The major QTL *cqGD2-2* was 129 cM from the 2HL_34260490, accounting for 18.55–20.41% PVE. The other 7 consensus QTLs were only detected at one interval in a particular year. None of the consensus QTLs were expressed at all intervals, and some QTLs showed opposite effects in different intervals.

**Figure 4:**
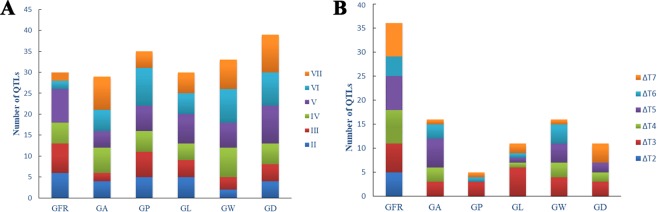
The number of QTLs for grain filling rate and five grain size traits at different stages. A Number of QTLs in each stage identified by unconditional mapping method. B Number of QTLs in each period identified by conditional mapping method.

In conclusion, most of the QTLs detected by the unconditional QTL mapping method were detected in the V, VI and VII stages, while the QTLs identified by the conditional QTL mapping method were expressed in different periods (Fig. 4).

In addition, to reduce the interference of Rt and Ct on the grain shape and grain filling rate, we used the genome-wide composite interval mapping (GCIM) to perform covariate QTL analysis with Rt, Ct, Rt + Ct as covariates, and detected 118, 109 and 84 QTLs on all seven chromosomes, respectively (Supplementary Table S4). Among these 311 covariate QTLs, 140 covariate QTLs were consistent with unconditional or conditional consensus QTLs, and the remaining 171 were new QTLs. Comparing the consensus QTLs for grain filling rate and grain size traits detected by the five QTL mapping methods, we identified 34 major consensus QTLs (repeatedly detected in at least two QTL mapping methods), including 2, 4, 7, 9, 5, 5, 1 and 1 consensus QTLs for GFR, GA, GP, GL, GW, GD, GFR_max_ and GFR_mean_, respectively (Table 4). Of these major consensus QTLs, most of them were identified in two QTL mapping methods, and only six were simultaneously identified in at least four QTL mapping methods.

**Table 4:** The major consensus QTLs identified for grain filling rate and five grain size traits using multiple mapping method.

Trait	Consensus QTLs	Chr.^a^	Position (cM)	LOD	R2b	Add^c^	Mapping method^d^
GFR	qcGFR2–3	2H	125.73	4.19–46.56	16.30–71.54	−0.28 to 0.51	Ct,UC,C
	qcGFR3–1	3H	32.63–32.72	3.06–10.61	1.40–28.61	−0.10 to 0.18	Rt,Ct,Rt + Ct,UC,C
GA	qcGA2–1	2H	124.44–126.63	3.17–49.18	12.39–75.68	0.30–2.89	Rt,Ct,Rt + Ct,UC,C
	qcGA3–2	3H	41.41	3.61–5.50	6.86–12.13	0.29–0.38	Rt,Ct,Rt + Ct,C
	qcGA7–2	7H	66.14	75.85–35.07	9.43–35.07	0.22–1.60	Rt,UC,C
	qcGA7–4	7H	92.90	12.61–13.47	10.99–36.31	0.51–1.10	Rt,UC
GP	qcGP2–1	2H	125.53	3.46–37.66	15.96–68.01	0.52–1.21	Ct,UC
	qcGP2–2	2H	133.61	5.93–25.32	20.26–24.71	0.79–0.90	Ct,UC
	qcGP7–2	7H	66.67	4.85–31.98	9.30–44.71	0.20–1.27	Rt,UC,C
	qcGP7–5	7H	115.22-118.25	4.78-17.12	11.32-21.04	0.56-0.62	Rt,Rt + Ct,UC
	qcGP7-1	7H	123.44	5.74-25.21	6.51-28.38	0.54-0.57	Ct,Rt + Ct,C
	qcGP7-3	7H	139.84-142.42	10.79-21.31	21.72-30.24	0.74-0.77	Ct,Rt + Ct,UC
	qcGP7-8	7H	151.09	5.48-22.28	8.48-30.90	−1.22 to 0.79	Rt,UC,C
GL	qcGL1	1H	18.81	3.02-8.28	10.36-14.00	0.21-0.32	Rt,Ct,Rt + Ct,C
	qcGL2-1	2 H	125.94-126.13	6.59-29.66	15.47-51.03	0.35-0.45	Ct,UC
	qcGL2-3	2H	133.1-133.61	4.52-5.79	12.84-17.26	0.31-0.36	Ct,UC
	qcGL7-2	7H	66.67	4.22-50.97	9.08-45.19	0.09-0.61	Rt,UC,C
	qcGL7-3	7H	91.38-93.97	4.19-13.23	3.74-38.03	0.06-0.33	Rt,Rt + Ct,C
	qcGL7-5	7H	116.44	10.26-22.32	21.79-37.53	0.27-0.40	Rt,UC
	qcGL7-6	7H	123.33-123.44	3.20-10.51	6.51-31.13	0.37-0.57	Rt,Ct,C
	qcGL7-7	7H	135.24-136.75	9.15-38.74	23.52-47.29	−0.48 to 0.55	Rt,Rt + Ct,UC
	qcGL7-8	7H	149.42-151.09	6.67-24.79	17.10-43.21	0.35-0.52	Rt,Rt + Ct,UC
GW	qcGW2-1	2H	52.61	4.08-4.26	15-15.51	−0.06	Ct,C
	qcGW2-4	2H	125.19	4.10-53.91	11.06-75.51	0.05-0.39	Ct,UC,C
	qcGW3-4	3H	30.33-31.47	3.26-6.31	2.16-23.36	−0.09 to 0.08	Rt,Ct,Rt + Ct,UC,C
	qcGW5	5H	3.58-4.49	3.43-11.83	10.66-11.83	−0.05	Rt,Ct,Rt + Ct,C
	qcGW7-2	7H	66.53	5.62-46.68	5.20-34.11	−0.18 to 0.26	Rt,UC
GD	qcGD2-2	2H	125.73	5.48-56.41	18.90-78.03	0.18-0.42	Ct,UC
	qcGD7-1	7H	65.75	4.12-44.92	9.80-31.65	0.12-0.26	Rt,UC
	qcGD7-2	7H	71.08	10.99-19.79	14.11-34.69	0.09-0.18	Rt,C
	qcGD7-3	7H	76.14	3.18-17.85	11.11-31.06	0.09-0.21	Rt,UC
	qcGD7-7	7H	151.09	9.59-31.02	6.33-34.85	0.11-0.30	Rt,UC
GFR_max_	qcGFR_max_2-3	2H	126.97	33.18-43.39	68.04-70.11	0.49-0.54	Ct,UC
GFR_mean_	qcGFR_mean_2	2H	125.19	35.14-56.94	66.66-74.83	0.16-0.18	Ct,UC

^a^Chromosome. ^b^The phenotypic variance explained by each QTL. ^c^Additive effect. ^d^Rt, covariate QTL analysis using row type as a covariate; Ct, covariate QTL analysis using caryopsis type as a covariate; Rt + Ct, covariate QTL analysis using row type and caryopsis type as a covariate; UC, unconditional QTL analysis; C, conditional QTL analysis. Abbreviations are shown in the footnote of Table 1.

### Integration of unconditional and conditional QTLs

Unconditional and conditional consensus QTLs were further integrated into unique QTLs (Details of unique QTLs were shown in Supplementary Table S5), and listed in Table 5. For the GFR, unique QTL (*uqGFR2-3*) was repeatedly detected at three different stages and multiple time intervals over two years. For the GA, unique QTL *uqGA2-2* was stably detected at five different periods and multiple intervals across two years. For the GL, *uqGL7-1* was steadily expressed in several different periods and time intervals. For the GW, unique QTL *uqGW2-4* was repeatedly expressed at all sampling stages. For unconditional and conditional QTL integration, some unique QTLs were detected at multiple stages, but not detected at the final stage, these unique QTLs might play an important role in the grain filling stage.

**Table 5:** Number of unique QTLs that integrated from the unconditional and conditional consensus QTLs for GFR and five grain size traits.

Trait	No. of uniques QTL	No. of QTL detected by unconditional	No. of QTL detected by conditional	No. of QTL detected by both conditional and unconditional
GFR	16	4	5	7
GA	23	12	9	2
GP	18	14	3	1
GL	18	11	6	1
GW	18	7	8	3
GD	25	16	6	3

### QTL clusters in genome

QTL clusters were defined as a region containing multiple QTLs of various traits within approximately 20 cM^53^. Total of 11 QTL clusters were detected on 1H (two clusters), 2H (one cluster), 3H (three clusters), 5H (one cluster), and 7H (four clusters) (Table 6). Among these QTL clusters, three QTL clusters (C1, C6 and C8) affected all grain size traits, and the cluster C3 affected all traits. The remaining seven QTL clusters affected at least four traits and three of them were associated with three different grain size traits. The consensus QTLs associated with all traits in C1 and C6 showed negative additive effects, and the alleles that increased GFR and grain size were from Huaai11. Conversely, the alleles of consensus QTLs that increased all grain size traits in C3 were from Huadamai6. In addition, QTLs in these three QTL clusters (C2, C7 and C10) increased GFR and grain size traits alleles were from different parents.

**Table 6:** The QTL clusters simultaneously affecting several traits in this study.

Clusters	Chr^a^	Marker interval	position (cM)	No. of QTLs	Physical position(Mb)	Traits
C1	1H	1H_37679977 – 1_306380656	18.42–22.5	7	415.6–429.8	GFR, GA, GP, GL, GW, GD
C2	1H	1H_58042355 – 1H_22104337	37.35–54.5	8	301.5–373.1	GFR, GP, GW, GD, GFR_max_
C3	2H	2HL_16222169 – M_149956_1482	115.5–133.57	18	622.6–668.0	GFR, GA, GP, GL, GW, GD, GFR_max_, GFR_mean_
C4	3H	3HL_32425970 – 3_504106156	17.5–34.13	12	626.2–672.6	GFR, GA, GL, GW, GD
C5	3H	6HL_29119759 – 3HL_24669457	39.93–57.5	5	585.4–622.4	GA, GP, GL, GW
C6	3H	3_411835196 – 3_428733082	82.5–98.5	6	438.1–512.9	GA, GP, GL, GW, GD
C7	5H	5HS_7374618 – 5_6313908	0–11.5	5	0.4–8.1	GFR, GA, GW, GD
C8	7H	7_552887354 – M_363857_407	56.5–70	15	543.7–585.3	GFR, GA, GP, GL, GW, GD, GFR_max_
C9	7H	7_428184911 – 7_270366894	92.14–110.5	8	262.1–482.4	GA, GP, GL, GD, GFR_mean_
C10	7H	7HL_22161891 – 7HL_28498244	120.5–140.5	9	248.8–423.1	GFR, GP, GL, GW
C11	7H	7HL_6335336 – M_1645585_244	150.5–153.5	5	227.8–345.7	GA, GP, GL, GD

^a^Chromosome. Abbreviations are shown in the footnote of Table 1.

## Discussion

Many studies have reported that genetic difference in grain yield was related to difference in GFR in barley^7,9,10^. In addition, GFR_mean_ and GFR_max_ have been reported in maize and wheat as important factors regulating grain weight^42,54^. The GFR_mean_ and GFR_max_ were significantly associated with TGW (r > 0.9) in our experiments (Fig. 2), indicating that GFR_mean_ and GFR_max_ also promoted grain weight in barley.

Grain development is a dynamic process that is regulated by three physiological stages: (1) grain formation period, mainly the division of endosperm cells and the formation of basic structure of seeds, during which there is almost no accumulation of dry matter; (2) the linear growth period of dry matter, the accumulation of dry matter in the most vigorous period of the grain, the grain weight during the period almost increased linearly; (3) maturity, the grain weight increased slowly during this period^18–21,55^. The substance accumulation mainly occurs in the linear growth period of dry matter. In this study, seven sampling from grain formation period to maturity were used to evaluate GFR. The GFR at stage III, IV and V were significantly positively correlated with TGW (r > 0.7), while, there was no correlation between the initial and final stages of GFR and TGW (Fig. 2). This results were basically consistent with the previous studies on the grain weight of linear dry matter accumulation period^20^.

Grain size can be divided into components such as GA, GP, GL, GW and GD^56,57^. In barley, previous studies on grain shape were mainly at maturity, and only major QTLs controlling GL traits were found^57–60^. However, QTLs detected at maturity may not observe their genetic effects during specific periods of crop development, and dynamic QTL analysis can better understand the developmental behavior of quantitative traits. We found that some of the QTLs for GFR and grain size traits identified on the 2H and 7H chromosomes co-localized with QTLs for yield-related traits, seedling traits and dwarf gene *btwd1* detected in previous studies. For example, certain major consensus QTLs tightly linked to 2_527241334 at 126 cM for grain filling rate and grain size detected here, are likely the same to seedling traits QTL *qSH2-191* and *qSFW2-191* identified by Wang *et al*.^61^ and also likely same to the *qSms2-7* and *qTgw2-1* for yield-related traits reported by Wang *et al*.^29^. In addition, a major QTL *ucqGL7-5* for grain length closely linked to the Bmac31 of 151 cM identified on 7H chromosome, which may be in the same locus as the dwarfing gene *btwd1* reported by Ren *et al*.^43^. The findings indicated that GFR and grain size traits are closely related to yield and yield-related traits.

In previous studies, QTLs for grain area were detected on chromosomes 1H, 2H, 3H, 4H, 5H and 6H^57,62,63^, and QTLs associated with grain perimeter were identified on chromosomes 1H and 3H^57^. QTLs associated with grain length and width were previously detected on all seven chromosomes^57–60,62–67^, and QTLs for grain diameter were detected on chromosomes 2H, 3H, 4H, 6H, and 7H^67^. Ayoub *et al*.^57^ and Sharma *et al*.^62^ reported a major QTL on chromosome 2H, nearby the locus *vrs1* and affected all grain size traits. Most of QTLs for grain sizes identified on 2H in this study were also distributed near the morphological marker *vrs1*, which are likely same to the QTL reported by Ayoub *et al*.^57^. Walker *et al*.^66^ detected two QTLs for grain length on 3H, located near markers 2_0662 and 2_1272, respectively. The physical location of the two genetic markers was queried using the Barleymap website (http://floresta.eead.csic.es/barleymap/), we found that the marker 3HL_42780152 in this experiment was close to the physical position of 2_1272, indicating that the QTL *cqGL3* adjacent to 3HL_42780152 is likely the same locus reported by Walker *et al*.^66^. Many new QTLs controlling grain size were detected in our experiment, in which some major QTLs (*ucqGA7-1*, *ucqGP7-2*, *cqGP7-2*, *ucqGL7-1* and *ucqGD7-1*) located near 65 cM of the 7H chromosome were repeatedly detected at different stages or at the same stage of different environments, indicating that these regions might be an important novel locus affecting grain size traits.

Dynamic QTL mapping identified 196 unconditional QTLs and 95 conditional QTLs (Supplementary Table S2, Table S3). Most QTLs were detected at V, VI and VII stages (Fig. 4). By integrating the unconditional QTL for GFR and grain size traits, some unconditional consensus QTLs were detected simultaneously at several stages, and the majority of the QTLs were identified at stages IV-VII. These results indicated that QTLs associated with grain size traits are selectively expressed during grain filling period. Additionally, we found that some unconditional and conditional consensus QTLs, such as *ucqGW3-2*, *cqGFR2-2*, and *cqGW3-1*, had a combination of identified QTLs with opposite additive effects, indicating that certain QTLs had different expression patterns at different developmental stages or environments. This expression pattern has also been reported for plant height in rapeseed^68^ and for grain filling rate in corn^69^.

In this study, 11 clustered QTL regions controlling grain filling rate and grain size traits were found on chromosomes 1H, 2H, 3H, 5H and 7H (Fig. 3, Table 6), and these co-localized QTLs were mainly concentrated on the chromosome 2H, 3H and 7H. Anchoring the SNP markers located in important QTL regions to the Morex genome via the barleymap website (http://floresta.eead.csic.es/barleymap/) identified eight related candidate genes^70^, of which, five associated with plant height and grain size, and three genes were involved in the biosynthesis and metabolism of starch and (1,3;1,4)-β-glucan. Notably, the most important QTL cluster region at the 2HL_16222169 - M_149956_1482 was located on chromosome 2H, containing 18 QTLs controlling all grain size and grain filling rate traits (Fig. 3, Table 6). The *vrs1* gene was detected in this region, which was reported in previous studies to control row-number phenotype, affecting grain size and thousand grain weight traits^29,57,61,62^. The *Nud* gene controlling the hulled/naked caryopsis was detected within QTL cluster affecting GFR, GFR_max_, GA, GP, GL, GW and GD in interval 56.5–70 cM on chromosome 7H, and QTLs affected TGW were located at this locus^71^. Since these two genes have large effects on the population used here, some of the minor-effect QTLs may be affected and difficult to detect. Therefore, to eliminate the interference of these two genes, we performed a covariate QTL analysis to find more new QTLs. Through covariate QTL analysis, we found that 171 (55%) of the new QTLs were undetected by either unconditional or conditional QTL mapping methods (Supplementary Table S4). The effects of these QTLs on grain filling rate and grain size traits are not as obvious as the *vrs1* and *Nud* genes, but they had an important role underlying these traits. Gibberellin 20-oxidase gene (*Hv20ox*_*2*_) was found in a QTL cluster for GFR, GA, GL, GW and GD between 3HL_32425970 and 3_504106156, located in the interval of 17.5–34.13 cM on chromosome 3H, which was a functional gene regulating barley *sdw1/denso*, and certain QTLs for yield, grain size and plumpness were co-localized with this gene^72,73^. Within another QTL cluster for GA, GP, GL and GW between 6HL_29119759 and 3 HL_24669457 on chromosome 3H, the flowering time gene *HvFT1* was detected, which was the dominant plant transition from vegetative state to reproductive state, affecting the flowering and maturity of barley^74,75^. Furthermore, within the QTL cluster associated with GA, GP, GL and GD in the interval of 150.5–153.5 cM on chromosome 7 H, the novel dwarf gene *btwd1* was identified, which not only affect plant height, but affect grain yield at the *btwd1* locus^29,43^.

The content of starch in barley is 62% to 77% of the grain dry weight, and the grain filling process is mainly the accumulation process of starch. The biosynthesis of starch mainly involves ADP glucose pyrophosphorylase, starch synthase, starch branching enzyme and starch debranching enzyme^76–78^. Interestingly, two genes related to starch biosynthesis and metabolism, as well as a gene involved in (1,3;1,4)-β-glucan synthesis, were found in three QTL clusters in this study. The *GBSS1b* gene, the key gene involved in amylose synthesis, was detected in the C3 region of chromosome 2H, which was close to the 2HL_22930294 marker. The *ucqGFR2-3* closely linked to the marker 2HL_22930294 was detected simultaneously at stage II over two years. The *GBSS1b* transcripts were abundant in the pericarp of flowering and initial grouting^79^, so we considered this to be a candidate gene for the QTL locus. The *amy2* gene, which was the most important gene for starch degradation during malting and saccharification^80^, was located in the C8 region of chromosome 7H, close to the 7HL_37199773 marker at 65 cM. Previous studies have reported QTLs underlying malt activity and amylopectin content at the *amy2* locus^81^. The *HvCslF6*, a key gene regulating (1,3;1,4)-β-D-glucans biosynthesis, was found in the C9 region of chromosome 7H^82^. This gene was expressed low in the early stage of grain development and then rapidly up-regulated as the activity of synthetase increased^83^.

In summary, we identified 90, 55 and 311 consensus QTLs using unconditional, conditional and covariate QTL mapping methods, respectively, and detected 34 main-effect QTLs that were simultaneously expressed at multiple stages. The results indicated that these major QTLs were not only expressed at maturity but were also in the early stages of grain development. In addition, eight predicted candidate genes involved in grain yield and starch synthesis pathways were identified in the six clustered QTL regions, which might play an important role in controlling GFR and grain size traits. These findings enhance our understanding of the genetic mechanism of barley grain filling process.

## Supplementary information

Mapping dynamic QTL dissects the genetic architecture of grain size and grain filling rate at different grain-filling stages in barley
