# Supplementary material for: Mapping dynamic QTL dissects the genetic architecture of grain size and grain filling rate at different grain-filling stages in barley
Source: Sci Rep. 2019 Dec 11;9:18823. doi: 10.1038/s41598-019-53620-5 (PMC6906516; doi:10.1038/s41598-019-53620-5)
Supplement: Supplementary file 1 — Mapping dynamic QTL dissects the genetic architecture of grain size and grain filling rate at different grain-filling stages in barley [file 41598_2019_53620_MOESM1_ESM.doc]

***Supplementary Information***

**Mapping dynamic QTL dissects the genetic architecture of grain size and grain filling rate at different grain-filling stages in barley**

Binbin Du, Qifei Wang, Genlou Sun, Xifeng Ren, Yun Cheng, Yixiang Wang, Song Gao, Chengdao Li, Dongfa Sun^*^

*Corresponding author

Dongfa Sun: sundongfa1@mail.hzau.edu.cn

**Supplementary Figure S1. The main climatic factors of the barley growth season in two years.**


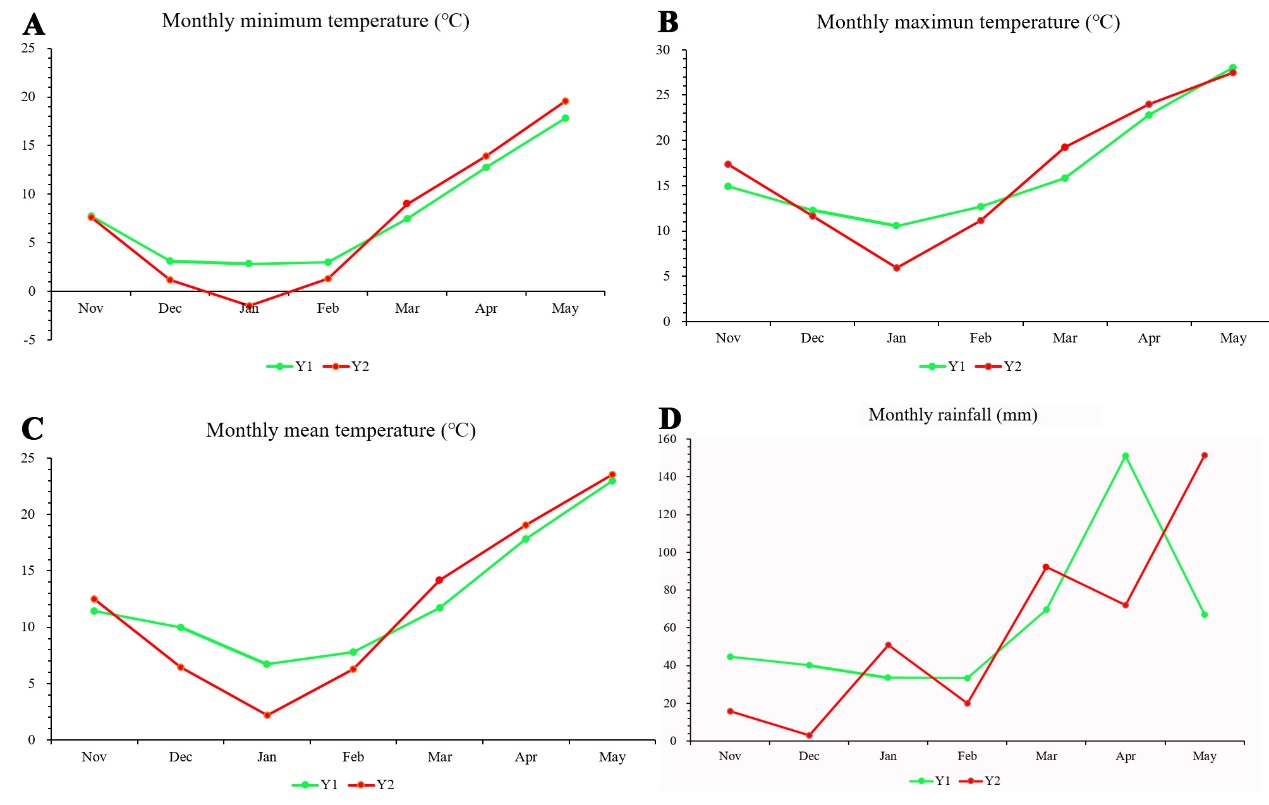


**Supplementary Figure S2. Frequency distributions of the five grain size traits in 122 doubled haploid (DH) lines. Y1 and Y2 represent 2017 and 2018, respectively, Abbreviations are shown in the footnote of Table 1.**


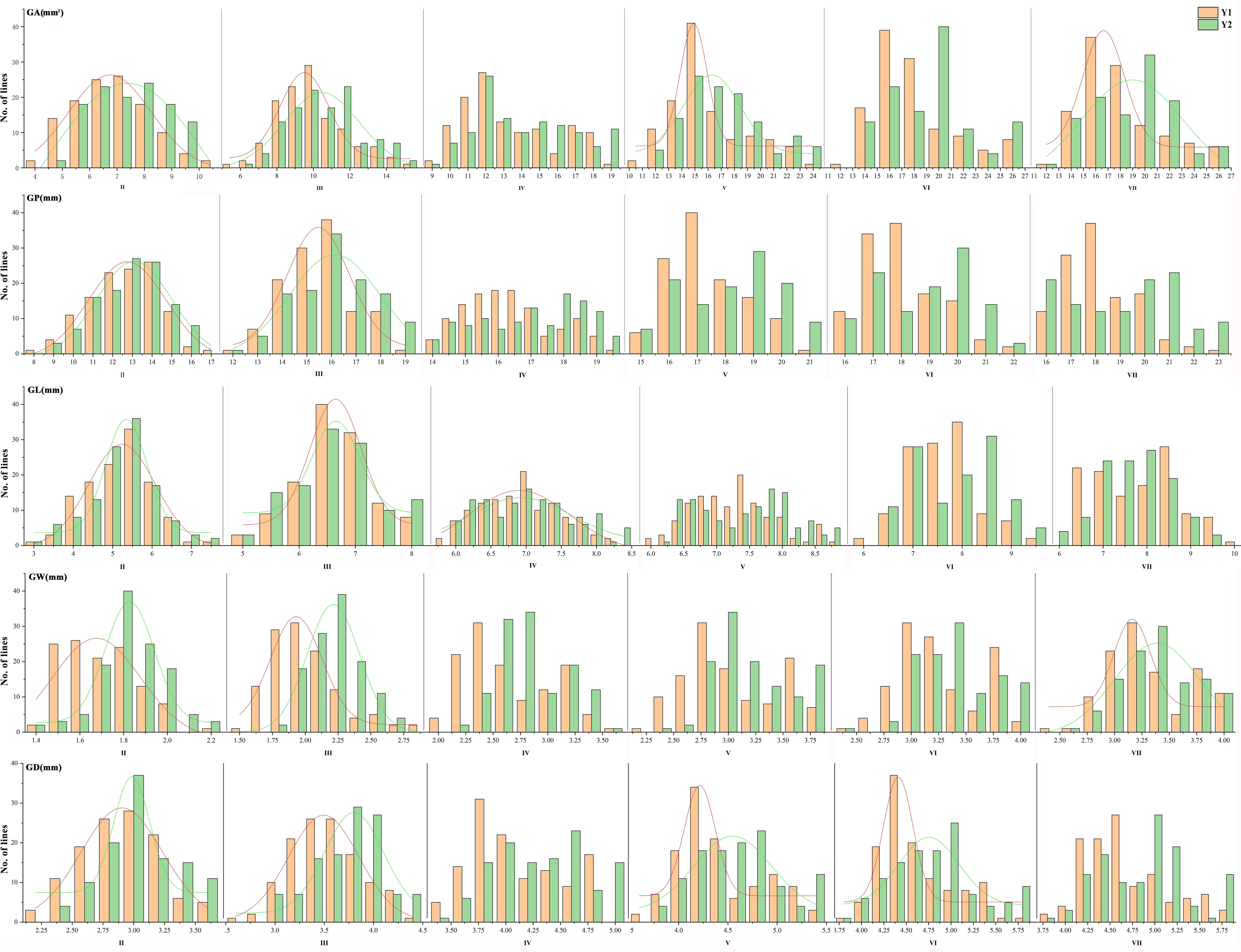


**Supplementary** **Table S1. Analysis of variance and broad-sense heritabilities for grain filling rate and five grain size traits in DH population over two years.** ^**^Significant at P < 0.01. Abbreviations are shown in the footnote of Table 1.

| Trait | Stage | ANOVA | | | *h_B_****^2^/%*** |
| --- | --- | --- | --- | --- | --- |
|  |  | G | G×E | E |  |
| GFR | I | 1.38^**^ | 0.27^**^ | 0.07^**^ | 83.4 |
|  | II | 6.4^**^ | 0.23^**^ | 0.52^**^ | 86.7 |
|  | III | 70.21^**^ | 1.68^**^ | 3.11^**^ | 96.9 |
|  | IV | 161.24^**^ | 6.34^**^ | 3.14^**^ | 96.6 |
|  | V | 70.33^**^ | 0.16^**^ | 3.17^**^ | 95.3 |
|  | VI | 29.17^**^ | 3.88^**^ | 0.06^**^ | 85.8 |
|  | VII | 8.21^**^ | 2.22^**^ | 0.04^**^ | 81.4 |
| GFR_mean_ |  | 18.47^**^ | 0.66^**^ | 0.28^**^ | 96.7 |
| GFR_max_ |  | 168.45^**^ | 6.64^**^ | 2.34^**^ | 95.4 |
| GA | II | 862.12^**^ | 18.54^**^ | 50^**^ | 96.2 |
|  | III | 1803.7^**^ | 30.5^**^ | 78.57^**^ | 98.2 |
|  | IV | 3401.36^**^ | 69.29^**^ | 97.26^**^ | 97.9 |
|  | V | 4244.03^**^ | 142.39^**^ | 158.35^**^ | 96.6 |
|  | VI | 5098.48^**^ | 370.81^**^ | 208.98^**^ | 92.7 |
|  | VII | 4650.85^**^ | 1610.03^**^ | 29.65^**^ | 65.1 |
| GP | II | 1406.68^**^ | 21.51^**^ | 20.38^**^ | 98.5 |
|  | III | 1071.66^**^ | 41.87^**^ | 41.23^**^ | 96.2 |
|  | IV | 982.5^**^ | 109.3^**^ | 78.74^**^ | 90 |
|  | V | 1008.94^**^ | 134.09^**^ | 82.31^**^ | 86.7 |
|  | VI | 880.51^**^ | 551.25^**^ | 56.28^**^ | 79.4 |
|  | VII | 1108.41^**^ | 666.08^**^ | 17.96^**^ | 72.8 |
| GL | II | 322.18^**^ | 8.97^**^ | 0.09^**^ | 97 |
|  | III | 244.85^**^ | 12.71^**^ | 0.04^**^ | 94.6 |
|  | IV | 169.6^**^ | 15.34^**^ | 1.46^**^ | 90.7 |
|  | V | 160.33^**^ | 66.14^**^ | 0.01^**^ | 81.7 |
|  | VI | 169.04^**^ | 128.44^**^ | 4.8^**^ | 76.5 |
|  | VII | 221.47^**^ | 132.71^**^ | 0.45^**^ | 65.7 |
| GW | II | 9.58^**^ | 1.81^**^ | 2.01^**^ | 75 |
|  | III | 22.25^**^ | 3.7^**^ | 5.98^**^ | 83.3 |
|  | IV | 56.2^**^ | 3.8^**^ | 4.77^**^ | 93.5 |
|  | V | 65.01^**^ | 14.38^**^ | 0.74^**^ | 77.8 |
|  | VI | 46.95^**^ | 14.69^**^ | 1.17^**^ | 69.2 |
|  | VII | 52.47^**^ | 13.18^**^ | 0.08^**^ | 75 |
| GD | II | 49.55^**^ | 3.01^**^ | 3.8^**^ | 92.9 |
|  | III | 58.3^**^ | 5.48^**^ | 7.02^**^ | 89.6 |
|  | IV | 81.4^**^ | 4.15^**^ | 6.81^**^ | 95.5 |
|  | V | 78.66^**^ | 18.03^**^ | 0.21^**^ | 76.9 |
|  | VI | 73.19^**^ | 25.75^**^ | 1.86^**^ | 63.9 |
|  | VII | 86.12^**^ | 35.13^**^ | 0.62^**^ | 58.9 |

**Supplementary Table S2. Detailed information of unconditional QTLs for grain filling rate and five grain size traits in DH population.**

| Trait | Consensus QTLs | | | |  | Identified QTLs | | | | | | |
| --- | --- | --- | --- | --- | --- | --- | --- | --- | --- | --- | --- | --- |
|  | Consensus QTLs | Peak | The closest marker | CI |  | Chr. | Position | LOD | R^2^ | Add | CI | Stages |
| GFR | ucqGFR1-1 | 37.79 | 1H_82851228 | 37.35-38.24 |  | 1H | 37 | 33.24 | 19.47 | 0.11 | 36.5-38.5 | Y1.II |
|  |  |  |  |  |  | 1H | 38 | 34.15 | 16.33 | 0.10 | 37.5-38.5 | Y2.II |
|  | ucqGFR1-2 | 42 | 1H_10863328 | 41.71-42.28 |  | 1H | 42 | 47.17 | 36.22 | -0.18 | 41.5-42.5 | Y2.II |
|  |  |  |  |  |  | 1H | 42 | 7.03 | 7.12 | -0.12 | 41.5-42.5 | Y2.III |
|  | ucqGFR1-3 | 54 | 1H_58188215 | 52.4-54.5 |  | 1H | 54 | 6.81 | 4.40 | -0.11 | 52.4-54.5 | Y2.IV |
|  | ucqGFR2-1 | 68.5 | M_1999039_479 | 68.28-68.87 |  | 2H | 68 | 4.12 | 2.69 | 0.09 | 67.5-68.5 | Y1.IV |
|  |  |  |  |  |  | 2H | 69 | 3.10 | 2.83 | 0.06 | 68.5-69.5 | Y2.III |
|  | ucqGFR2-2 | 88 | Bmag829 | 87.5-89.5 |  | 2H | 88 | 4.85 | 12.20 | -0.05 | 87.5-89.5 | Y2.VII |
|  | ucqGFR2-3 | 126.25 | 2HL_22930005 | 125.75-127.5 |  | 2H | 123 | 21.36 | 46.57 | 0.28 | 122.5-123.5 | Y1.V |
|  |  |  |  |  |  | 2H | 123 | 19.64 | 41.63 | 0.25 | 122.5-123.5 | Y2.V |
|  |  |  |  |  |  | 2H | 127 | 15.57 | 33.71 | 0.08 | 126.5-127.5 | Y1.II |
|  |  |  |  |  |  | 2H | 127 | 36.22 | 61.85 | 0.32 | 126.5-127.5 | Y1.III |
|  |  |  |  |  |  | 2H | 127 | 44.95 | 71.54 | 0.51 | 126.5-127.5 | Y1.IV |
|  |  |  |  |  |  | 2H | 127 | 35.35 | 60.59 | 0.29 | 126.5-127.5 | Y2.III |
|  |  |  |  |  |  | 2H | 127 | 46.56 | 71.32 | 0.47 | 126.5-127.5 | Y2.IV |
|  | ucqGFR3-1 | 28 | 3_525094736 | 27.64-28.35 |  | 3H | 27 | 6.04 | 9.66 | 0.13 | 26.5-27.5 | Y1.V |
|  |  |  |  |  |  | 3H | 29 | 8.81 | 14.26 | 0.14 | 28.5-29.5 | Y2.V |
|  | ucqGFR3-2 | 32 | 3HL_15958290 | 31.71-32.28 |  | 3H | 31 | 5.14 | 4.76 | -0.08 | 30.5-31.5 | Y2.III |
|  |  |  |  |  |  | 3H | 32 | 5.71 | 10.12 | -0.04 | 31.5-32.5 | Y1.II |
|  |  |  |  |  |  | 3H | 33 | 6.93 | 6.48 | -0.10 | 32.5-34.5 | Y1.III |
|  |  |  |  |  |  | 3H | 33 | 4.39 | 16.97 | 0.09 | 32.5-34.5 | Y1.VI |
|  |  |  |  |  |  | 3H | 33 | 7.32 | 24.12 | 0.11 | 32.5-34.5 | Y2.VI |
|  |  |  |  |  |  | 3H | 33 | 5.78 | 14.92 | 0.05 | 32.5-34.5 | Y2.VII |
|  | ucqGFR5 | 0 | 5HS_7374618 | 0-0.5 |  | 5H | 0 | 4.12 | 5.76 | -0.09 | 0-0.5 | Y1.V |
|  |  |  |  |  |  | 5H | 0 | 4.39 | 6.24 | -0.08 | 0-0.5 | Y2.V |
|  | ucqGFR7-1 | 66.5 | 7_501748124 | 66.25-66.75 |  | 7H | 65 | 12.69 | 9.96 | 0.17 | 64.5-65.5 | Y1.IV |
|  |  |  |  |  |  | 7H | 67 | 5.35 | 7.66 | 0.10 | 66.5-67.5 | Y1.V |
|  |  |  |  |  |  | 7H | 67 | 6.02 | 9.16 | 0.10 | 66.5-67.5 | Y2.V |
|  | ucqGFR7-2 | 180 | 7HS_16458224 | 179.5-180.5 |  | 7H | 180 | 4.74 | 8.07 | 0.03 | 179.5-180.5 | Y1.II |
|  |  |  |  |  |  | 7H | 180 | 8.18 | 7.63 | 0.10 | 179.5-180.5 | Y1.III |
| GA | ucqGA1-1 | 22 | 1_306394013 | 21.64-22.35 |  | 1H | 22 | 3.57 | 8.64 | -0.41 | 21.5-22.5 | Y1.II |
|  |  |  |  |  |  | 1H | 22 | 3.23 | 7.65 | -0.38 | 21.5-22.5 | Y2.II |
|  | ucqGA1-2 | 64 | M_2579923_225 | 62.5-64.5 |  | 1H | 64 | 16.69 | 7.44 | 0.81 | 62.5-64.5 | Y1.IV |
|  | ucqGA1-3 | 87 | 1H_2112459 | 86.5-87.5 |  | 1H | 87 | 26.95 | 15.06 | -1.15 | 86.5-87.5 | Y1.IV |
|  | ucqGA1-4 | 117 | 1_41186142 | 116.5-117.5 |  | 1H | 117 | 5.49 | 3.32 | -0.48 | 116.5-117.5 | Y2.IV |
|  | ucqGA1-5 | 133 | 1_19825452 | 130.5-133.5 |  | 1H | 133 | 4.60 | 4.31 | -0.50 | 131.5-133.5 | Y2.VI |
|  |  |  |  |  |  | 1H | 133 | 3.92 | 1.29 | -0.39 | 130.5-133.5 | Y2.VII |
|  | ucqGA2 | 126.13 | 2_527241334 | 125.55-126.64 |  | 2H | 125 | 11.81 | 33.82 | 0.89 | 124.5-125.5 | Y1.II |
|  |  |  |  |  |  | 2H | 125 | 53.68 | 56.51 | 2.45 | 124.5-125.5 | Y1.IV |
|  |  |  |  |  |  | 2H | 125 | 9.51 | 25.91 | 0.76 | 124.5-125.5 | Y2.II |
|  |  |  |  |  |  | 2H | 125 | 55.06 | 57.63 | 2.85 | 124.5-125.5 | Y2.VII |
|  |  |  |  |  |  | 2H | 127 | 29.68 | 58.77 | 1.55 | 126.5-127.5 | Y1.III |
|  |  |  |  |  |  | 2H | 127 | 55.19 | 75.68 | 2.68 | 126.5-127.5 | Y1.V |
|  |  |  |  |  |  | 2H | 127 | 44.23 | 71.36 | 3.03 | 126.5-127.5 | Y1.VI |
|  |  |  |  |  |  | 2H | 127 | 42.95 | 71.64 | 3.04 | 126.5-127.5 | Y1.VII |
|  |  |  |  |  |  | 2H | 127 | 28.28 | 54.29 | 1.59 | 126.5-127.5 | Y2.III |
|  |  |  |  |  |  | 2H | 127 | 47.26 | 71.58 | 2.47 | 126.5-127.5 | Y2.IV |
|  |  |  |  |  |  | 2H | 127 | 47.18 | 58.70 | 2.63 | 126.5-127.5 | Y2.V |
|  | ucqGA3-1 | 30 | 3_510997641 | 29.5-30.5 |  | 3H | 30 | 4.39 | 1.54 | 0.46 | 29.5-30.5 | Y2.VII |
|  | ucqGA3-2 | 57 | 3HL_34537138 | 56.5-57.5 |  | 3H | 57 | 5.13 | 1.70 | 0.45 | 56.5-57.5 | Y2.VII |
|  | ucqGA3-3 | 91 | 3_267212934 | 90.5-91.5 |  | 3H | 91 | 5.03 | 4.85 | -0.53 | 90.5-91.5 | Y2.VI |
|  | ucqGA4 | 10 | 4HL_29463683 | 9.5-11.5 |  | 4H | 10 | 3.931 | 1.74 | -0.37 | 9.5-11.5 | Y1.V |
|  | ucqGA5 | 141 | M_1634918_588 | 140.5-141.5 |  | 5H | 141 | 3.19 | 1.43 | -0.35 | 140.5-141.5 | Y1.V |
|  | ucqGA7-1 | 65 | 7HL_8312277 | 64.71-65.28 |  | 7H | 65 | 12.66 | 9.43 | 0.80 | 64.5-65.5 | Y2.IV |
|  |  |  |  |  |  | 7H | 65 | 22.30 | 30.11 | 1.32 | 64.5-65.5 | Y2.VI |
|  |  |  |  |  |  | 7H | 65 | 35.07 | 22.32 | 1.60 | 64.5-65.5 | Y2.VII |
|  | ucqGA7-2 | 94 | Bmag746 | 93.64-94.35 |  | 7H | 94 | 13.42 | 10.99 | 1.07 | 93.5-94.5 | Y1.VI |
|  |  |  |  |  |  | 7H | 94 | 13.47 | 11.34 | 1.10 | 93.5-94.5 | Y1.VII |
|  | ucqGA7-3 | 151 | Bmac31 | 150.5-151.5 |  | 7H | 151 | 15.61 | 6.44 | 0.87 | 150.5-151.5 | Y2.VII |
| GP | ucqGP1-1 | 22 | 1_306394013 | 21.5-22.5 |  | 1H | 22 | 4.04 | 10.26 | -0.54 | 21.5-22.5 | Y1.II |
|  | ucqGP1-2 | 42 | 1H_10863328 | 41.5-42.5 |  | 1H | 42 | 3.62 | 7.82 | -0.51 | 41.5-42.5 | Y2.II |
|  | ucqGP2-1 | 126.25 | 2_527241334 | 125.93-126.54 |  | 2H | 125 | 8.21 | 22.64 | 0.89 | 124.5-125.5 | Y1.II |
|  |  |  |  |  |  | 2H | 125 | 37.66 | 59.45 | 1.12 | 124.5-125.5 | Y1.V |
|  |  |  |  |  |  | 2H | 126 | 13.56 | 15.96 | 0.52 | 125.5-126.5 | Y1.VI |
|  |  |  |  |  |  | 2H | 125 | 8.23 | 18.98 | 0.87 | 124.5-125.5 | Y2.II |
|  |  |  |  |  |  | 2H | 125 | 28.03 | 44.87 | 0.98 | 124.5-125.5 | Y2.III |
|  |  |  |  |  |  | 2H | 125 | 23.49 | 33.66 | 0.86 | 124.5-125.5 | Y2.IV |
|  |  |  |  |  |  | 2H | 125 | 27.30 | 24.38 | 1.10 | 124.5-125.5 | Y2.VI |
|  |  |  |  |  |  | 2H | 125 | 24.51 | 29.18 | 1.11 | 124.5-125.5 | Y2.VII |
|  |  |  |  |  |  | 2H | 127 | 28.38 | 46.82 | 1.06 | 126.5-127.5 | Y1.III |
|  |  |  |  |  |  | 2H | 127 | 36.24 | 62.17 | 1.14 | 126.5-127.5 | Y1.IV |
|  |  |  |  |  |  | 2H | 128 | 15.01 | 18.23 | 0.55 | 127.5-127.5 | Y1.VI |
|  |  |  |  |  |  | 2H | 127 | 27.69 | 57.21 | 1.21 | 126.5-127.5 | Y1.VII |
|  | ucqGP2-2 | 132 | 2HL_43143355 | 131.5-132.5 |  | 2H | 132 | 25.32 | 24.71 | 0.90 | 131.5-132.5 | Y2.V |
|  | ucqGP3-1 | 55 | 3HL_48064911 | 54.13-55.86 |  | 3H | 55 | 4.61 | 3.64 | 0.25 | 53.5-56.5 | Y1.V |
|  |  |  |  |  |  | 3H | 55 | 9.21 | 10.44 | 0.39 | 53.5-56.5 | Y1.VI |
|  |  |  |  |  |  | 3H | 55 | 3.39 | 3.25 | 0.24 | 53.5-56.5 | Y2.IV |
|  | ucqGP3-2 | 91 | 3_267212934 | 90.64-91.35 |  | 3H | 91 | 6.36 | 5.14 | -0.30 | 90.5-91.5 | Y1.V |
|  |  |  |  |  |  | 3H | 91 | 11.91 | 13.83 | -0.45 | 90.5-91.5 | Y1.VI |
|  | ucqGP5 | 203 | 5_226253827 | 202.5-203.5 |  | 5H | 203 | 4.76 | 4.78 | -0.26 | 202.5-203.5 | Y1.VI |
|  | ucqGP6 | 102 | M_1661027_233 | 101.5-102.5 |  | 6H | 102 | 30.3724 | 31.46 | 0.94 | 101.5-102.5 | Y2.V |
|  | ucqGP7-1 | 49 | GBM1102 | 47.5-50.5 |  | 7H | 49 | 5.26 | 5.45 | 0.31 | 47.5-50.5 | Y2.III |
|  | ucqGP7-2 | 65.5 | 7HL_37199773 | 65.25-65.75 |  | 7H | 67 | 11.84 | 13.73 | 0.49 | 66.5-67.5 | Y2.IV |
|  |  |  |  |  |  | 7H | 65 | 26.25 | 25.00 | 0.83 | 64.5-65.5 | Y2.V |
|  |  |  |  |  |  | 7H | 65 | 30.38 | 28.86 | 1.08 | 64.5-65.5 | Y2.VI |
|  |  |  |  |  |  | 7H | 65 | 31.98 | 44.71 | 1.23 | 64.5-65.5 | Y2.VII |
|  | ucqGP7-3 | 94 | Bmag746 | 93.64-94.35 |  | 7H | 94 | 9.36 | 9.99 | 0.42 | 93.5-94.5 | Y2.III |
|  |  |  |  |  |  | 7H | 94 | 14.66 | 13.89 | 0.69 | 93.5-94.5 | Y2.VII |
|  | ucqGP7-4 | 116 | 5_496886371 | 115.5-116.5 |  | 7H | 116 | 17.12 | 21.04 | 0.61 | 115.5-116.5 | Y2.III |
|  | ucqGP7-5 | 136 | 7HS_29196961 | 135.5-136.5 |  | 7H | 136 | 27.36 | 26.39 | -1.04 | 135.5-136.5 | Y2.VI |
|  | ucqGP7-6 | 140 | 7_319506952 | 138.5-140.5 |  | 7H | 140 | 21.31 | 30.24 | 0.77 | 138.5-140.5 | Y1.III |
|  | ucqGP7-7 | 151.8 | 2_287569753 | 151.44-152.11 |  | 7H | 153 | 15.16 | 18.76 | 0.52 | 152.5-153.5 | Y1.VI |
|  |  |  |  |  |  | 7H | 150 | 7.18 | 16.00 | 0.73 | 149.5-151.5 | Y2.II |
|  |  |  |  |  |  | 7H | 151 | 22.28 | 30.90 | 0.75 | 150.5-151.5 | Y2.IV |
| GL | ucqGL1 | 22 | 1_306394013 | 21.5-22.5 |  | 1H | 22 | 5.33 | 12.70 | -0.29 | 21.5-22.5 | Y1.II |
|  | ucqGL2-1 | 125.9 | 2_527241334 | 125.76-126.11 |  | 2H | 125 | 21.21 | 35.23 | 0.42 | 124.5-125.5 | Y1.III |
|  |  |  |  |  |  | 2H | 124 | 23.78 | 39.90 | 0.39 | 123.5-124.5 | Y1.IV |
|  |  |  |  |  |  | 2H | 125 | 23.72 | 32.56 | 0.37 | 124.5-125.5 | Y1.V |
|  |  |  |  |  |  | 2H | 129 | 12.44 | 22.57 | 0.36 | 127.5-129.5 | Y1.VI |
|  |  |  |  |  |  | 2H | 129 | 12.58 | 21.98 | 0.38 | 127.5-129.5 | Y1.VII |
|  |  |  |  |  |  | 2H | 124 | 19.67 | 51.03 | 0.45 | 123.5-124.5 | Y2.III |
|  |  |  |  |  |  | 2H | 127 | 24.31 | 45.80 | 0.37 | 126.5-127.5 | Y2.IV |
|  |  |  |  |  |  | 2H | 127 | 29.43 | 37.10 | 0.39 | 126.5-127.5 | Y2.V |
|  |  |  |  |  |  | 2H | 127 | 23.91 | 16.88 | 0.35 | 126.5-127.5 | Y2.VI |
|  |  |  |  |  |  | 2H | 127 | 29.66 | 15.52 | 0.40 | 126.5-127.5 | Y2.VII |
|  | ucqGL2-2 | 133.1 | 2_534686550 | 132.62-133.57 |  | 2H | 134 | 5.32 | 12.84 | 0.31 | 132.5-135.5 | Y1.II |
|  |  |  |  |  |  | 2H | 133 | 5.79 | 14.43 | 0.34 | 132.5-133.5 | Y2.II |
|  | ucqGL3-1 | 48 | 3HL_33828484 | 47.18-47.87 |  | 3H | 49 | 4.35 | 3.96 | 0.12 | 48.5-49.5 | Y1.V |
|  |  |  |  |  |  | 3H | 47 | 5.53 | 7.23 | 0.13 | 46.5-47.5 | Y2.IV |
|  | ucqGL3-2 | 91 | 3_267212934 | 90.5-91.5 |  | 3H | 91 | 6.59 | 6.36 | -0.15 | 90.5-91.5 | Y1.V |
|  | ucqGL5 | 157 | 5_306133226 | 156.5-157.5 |  | 5H | 157 | 3.25 | 2.94 | -0.11 | 156.5-157.5 | Y1.V |
|  | ucqGL7-1 | 65.25 | 7HL_13143105 | 65.0-65.5 |  | 7H | 66 | 15.66 | 25.01 | 0.25 | 65.5-66.5 | Y2.IV |
|  |  |  |  |  |  | 7H | 65 | 32.96 | 45.19 | 0.39 | 64.5-65.5 | Y2.V |
|  |  |  |  |  |  | 7H | 65 | 41.70 | 44.64 | 0.50 | 64.5-65.5 | Y2.VI |
|  |  |  |  |  |  | 7H | 65 | 50.97 | 44.63 | 0.61 | 64.5-65.5 | Y2.VII |
|  | ucqGL7-2 | 116 | 5_496886371 | 115.5-116.5 |  | 7H | 116 | 10.26 | 21.79 | 0.27 | 115.5-116.5 | Y2.III |
|  | ucqGL7-3 | 136 | 7HS_29196961 | 135.64-136.35 |  | 7H | 136 | 28.14 | 23.52 | -0.37 | 135.5-136.5 | Y2.VI |
|  |  |  |  |  |  | 7H | 136 | 38.74 | 27.20 | -0.48 | 135.5-136.5 | Y2.VII |
|  | ucqGL7-4 | 140 | 7_319506952 | 138.5-140.5 |  | 7H | 140 | 22.26 | 37.83 | 0.40 | 138.5-140.5 | Y1.III |
|  | ucqGL7-5 | 150.9 | Bmac31 | 150.64-151.2 |  | 7H | 151 | 24.79 | 34.52 | 0.35 | 150.5-151.5 | Y1.V |
|  |  |  |  |  |  | 7H | 151 | 19.74 | 42.09 | 0.45 | 150.5-151.5 | Y1.VI |
|  |  |  |  |  |  | 7H | 151 | 20.64 | 43.21 | 0.50 | 150.5-151.5 | Y1.VII |
|  |  |  |  |  |  | 7H | 150 | 6.67 | 17.10 | 0.35 | 149.5-151.5 | Y2.II |
|  | ucqGL7-6 | 175 | 7_144173681 | 174.5-175.5 |  | 7H | 175 | 3.20 | 7.47 | 0.22 | 174.5-175.5 | Y1.II |
| GW | ucqGW1-1 | 19.76 | 1H_45582581 | 19.42-20.09 |  | 1H | 22 | 7.31 | 5.45 | -0.09 | 21.5-22.5 | Y1.IV |
|  |  |  |  |  |  | 1H | 18 | 6.15 | 4.47 | -0.09 | 17.5-18.5 | Y1.V |
|  |  |  |  |  |  | 1H | 17 | 5.08 | 2.71 | -0.06 | 15.5-18.5 | Y2.V |
|  |  |  |  |  |  | 1H | 17 | 6.65 | 3.39 | -0.06 | 14.5-18.5 | Y2.VII |
|  | ucqGW1-2 | 48 | 1_167351152 | 47.5-48.5 |  | 1H | 48 | 6.47 | 3.25 | -0.06 | 47.5-48.5 | Y2.VI |
|  | ucqGW2 | 124.69 | 2HL_18970523 | 124.44-125.25 |  | 2H | 123 | 8.21 | 29.25 | 0.09 | 122.5-123.5 | Y2.II |
|  |  |  |  |  |  | 2H | 125 | 21.87 | 50.15 | 0.13 | 124.5-125.5 | Y1.II |
|  |  |  |  |  |  | 2H | 125 | 19.35 | 49.92 | 0.20 | 124.5-125.5 | Y1.III |
|  |  |  |  |  |  | 2H | 125 | 44.12 | 73.04 | 0.36 | 124.5-125.5 | Y1.IV |
|  |  |  |  |  |  | 2H | 125 | 44.23 | 74.18 | 0.39 | 124.5-125.5 | Y1.V |
|  |  |  |  |  |  | 2H | 125 | 55.61 | 73.61 | 0.36 | 124.5-125.5 | Y1.VI |
|  |  |  |  |  |  | 2H | 125 | 53.91 | 75.49 | 0.35 | 124.5-125.5 | Y1.VII |
|  |  |  |  |  |  | 2H | 125 | 17.15 | 43.08 | 0.14 | 124.5-125.5 | Y2.III |
|  |  |  |  |  |  | 2H | 125 | 45.78 | 32.27 | 0.28 | 124.5-125.5 | Y2.IV |
|  |  |  |  |  |  | 2H | 125 | 51.32 | 75.51 | 0.35 | 124.5-125.5 | Y2.V |
|  |  |  |  |  |  | 2H | 125 | 50.07 | 68.21 | 0.32 | 124.5-125.5 | Y2.VI |
|  |  |  |  |  |  | 2H | 125 | 51.01 | 70.81 | 0.33 | 124.5-125.5 | Y2.VII |
|  | ucqGW3-1 | 26 | 3_528470541 | 25.5-26.5 |  | 3H | 26 | 7.01 | 3.06 | 0.07 | 25.5-26.5 | Y1.VI |
|  | ucqGW3-2 | 31.33 | 2HL_33741786 | 31.04-31.62 |  | 3H | 32 | 3.62 | 6.75 | -0.07 | 31.5-32.5 | Y1.III |
|  |  |  |  |  |  | 3H | 30 | 4.75 | 2.38 | 0.06 | 29.5-30.5 | Y2.VI |
|  |  |  |  |  |  | 3H | 32 | 4.17 | 2.16 | 0.06 | 31.5-32.5 | Y2.VII |
|  | ucqGW3-3 | 52 | 3HL_45910009 | 51.5-52.5 |  | 3H | 52 | 3.53 | 1.43 | -0.05 | 51.5-52.5 | Y1.VI |
|  | ucqGW4 | 52 | 4_474989327 | 51.64-52.35 |  | 4H | 52 | 4.09 | 1.18 | -0.05 | 51.5-52.5 | Y2.IV |
|  |  |  |  |  |  | 4H | 52 | 4.48 | 2.32 | -0.05 | 51.5-52.5 | Y2.VII |
|  | ucqGW6 | 25 | 6_518846666 | 23.5-25.5 |  | 6H | 25 | 3.79 | 1.63 | -0.05 | 23.5-25.5 | Y1.VII |
|  | ucqGW7-1 | 57 | 1_4088556 | 56.5-57.5 |  | 7H | 57 | 5.82 | 4.28 | 0.08 | 56.5-57.5 | Y1.IV |
|  | ucqGW7-2 | 65.13 | 7HL_13143105 | 64.78-65.66 |  | 7H | 64 | 14.77 | 7.51 | 0.10 | 62.5-64.5 | Y1.VI |
|  |  |  |  |  |  | 7H | 64 | 11.86 | 6.02 | 0.09 | 62.5-64.5 | Y1.VII |
|  |  |  |  |  |  | 7H | 64 | 30.55 | 17.13 | -0.18 | 63.5-64.5 | Y2.IV |
|  |  |  |  |  |  | 7H | 67 | 7.63 | 5.61 | 0.10 | 66.5-67.5 | Y1.V |
|  |  |  |  |  |  | 7H | 66 | 46.68 | 34.11 | 0.26 | 65.5-66.5 | Y2.IV |
|  |  |  |  |  |  | 7H | 66 | 10.41 | 6.21 | 0.09 | 65.5-66.5 | Y2.V |
|  |  |  |  |  |  | 7H | 67 | 16.43 | 10.29 | 0.11 | 65.5-66.5 | Y2.VI |
| GD | ucqGD1-1 | 22 | 1_306394013 | 21.64-22.35 |  | 1H | 22 | 3.94 | 9.90 | -0.10 | 21.5-22.5 | Y1.II |
|  |  |  |  |  |  | 1H | 22 | 4.05 | 8.34 | -0.11 | 21.5-22.5 | Y2.II |
|  | ucqGD1-2 | 42 | 1H_10863328 | 41.5-42.5 |  | 1H | 42 | 4.14 | 5.92 | -0.09 | 41.5-42.5 | Y2.III |
|  | ucqGD1-3 | 64 | M_2579923_225 | 62.5-64.5 |  | 1H | 64 | 16.54 | 7.09 | 0.12 | 62.5-64.5 | Y1,IV |
|  | ucqGD1-4 | 89.17 | Bmag770 | 88.89-89.46 |  | 1H | 87 | 27.03 | 14.59 | -0.18 | 86.5-87.5 | Y1.IV |
|  |  |  |  |  |  | 1H | 87 | 24.81 | 13.84 | -0.18 | 86.5-87.5 | Y1.V |
|  |  |  |  |  |  | 1H | 92 | 14.26 | 6.30 | 0.12 | 91.5-92.5 | Y1.V |
|  | ucqGD2 | 125.93 | 2_527241334 | 125.69-126.44 |  | 2H | 125 | 10.38 | 29.87 | 0.18 | 124.5-125.5 | Y1.II |
|  |  |  |  |  |  | 2H | 125 | 55.02 | 57.72 | 0.38 | 124.5-125.5 | Y1.VI |
|  |  |  |  |  |  | 2H | 125 | 52.44 | 72.30 | 0.39 | 124.5-125.5 | Y1,IV |
|  |  |  |  |  |  | 2H | 125 | 8.35 | 18.90 | 0.18 | 124.5-125.5 | Y2.II |
|  |  |  |  |  |  | 2H | 124 | 35.67 | 61.95 | 0.33 | 123.5-124.5 | Y2.IV |
|  |  |  |  |  |  | 2H | 125 | 56.41 | 52.15 | 0.36 | 124.5-125.5 | Y2.V |
|  |  |  |  |  |  | 2H | 125 | 44.30 | 64.06 | 0.38 | 124.5-125.5 | Y2.VI |
|  |  |  |  |  |  | 2H | 125 | 56.16 | 51.54 | 0.37 | 124.5-125.5 | Y2.VII |
|  |  |  |  |  |  | 2H | 127 | 26.66 | 54.87 | 0.28 | 126.5-127.5 | Y1.III |
|  |  |  |  |  |  | 2H | 127 | 54.62 | 61.27 | 0.42 | 126.5-127.5 | Y1.V |
|  |  |  |  |  |  | 2H | 127 | 50.38 | 78.03 | 0.41 | 126.5-127.5 | Y1.VII |
|  |  |  |  |  |  | 2H | 127 | 17.91 | 30.74 | 0.24 | 126.5-127.5 | Y2.III |
|  | ucqGD3-1 | 27 | 3_529115904 | 26.5-27.5 |  | 3H | 27 | 4.19 | 1.96 | 0.06 | 26.5-27.5 | Y1.VI |
|  | ucqGD3-2 | 32 | 3HL_15958290 | 31.5-32.5 |  | 3H | 32 | 9.02 | 2.93 | 0.09 | 31.5-32.5 | Y2.VII |
|  | ucqGD3-3 | 90 | 3HL_14205585 | 89.64-90.35 |  | 3H | 91 | 4.78 | 2.29 | -0.06 | 90.5-91.5 | Y1.VI |
|  |  |  |  |  |  | 3H | 89 | 4.49 | 2.46 | -0.07 | 88.5-89.5 | Y1.VII |
|  | ucqGD4-1 | 12 | 4_528451537 | 11.5-12.5 |  | 4H | 12 | 4.22 | 1.22 | -0.05 | 11.5-12.5 | Y2.V |
|  | ucqGD4-2 | 52 | 4_474989327 | 51.5-52.5 |  | 4H | 52 | 4.59 | 1.39 | -0.05 | 51.5-52.5 | Y2.VII |
|  | ucqGD5-1 | 102 | 5HS_16446198 | 100.5-102.5 |  | 5H | 102 | 11.71 | 3.87 | 0.10 | 100.5-102.5 | Y2.V |
|  | ucqGD5-2 | 123.5 | 5HS_4157152 | 122.79-124.2 |  | 5H | 125 | 21.31 | 8.72 | -0.14 | 123.5-125.5 | Y2.V |
|  |  |  |  |  |  | 5H | 122 | 4.05 | 1.12 | -0.05 | 121.5-123.5 | Y2.VII |
|  | ucqGD5-3 | 155 | M_81421_1318 | 154.5-156.5 |  | 5H | 155 | 5.41 | 2.71 | -0.07 | 154.5-156.5 | Y1.VI |
|  | ucqGD6 | 78 | GBM1256 | 77.5-78.5 |  | 6H | 78 | 5.54 | 1.66 | 0.06 | 77.5-78.5 | Y2.V |
|  | ucqGD7-1 | 65.88 | 7HL_37199773 | 65.63-66.12 |  | 7H | 64 | 16.83 | 10.08 | 0.13 | 62.5-64.5 | Y1.VI |
|  |  |  |  |  |  | 7H | 66 | 13.71 | 14.97 | 0.14 | 65.5-66.5 | Y2.IV |
|  |  |  |  |  |  | 7H | 66 | 35.20 | 19.94 | 0.20 | 65.5-66.5 | Y2.V |
|  |  |  |  |  |  | 7H | 66 | 17.68 | 13.75 | 0.16 | 65.5-66.5 | Y2.VI |
|  |  |  |  |  |  | 7H | 66 | 44.92 | 31.65 | 0.26 | 65.5-66.5 | Y2.VII |
|  | ucqGD7-2 | 76 | 7_500661278 | 75.5-76.5 |  | 7H | 76 | 17.85 | 31.06 | 0.21 | 75.5-76.5 | Y2.III |
|  | ucqGD7-3 | 94 | Bmag746 | 93.5-94.5 |  | 7H | 94 | 10.79 | 3.43 | 0.09 | 93.5-94.5 | Y2.VII |
|  | ucqGD7-4 | 151 | Bmac31 | 150.5-151.5 |  | 7H | 151 | 9.59 | 6.33 | 0.11 | 150.5-151.5 | Y2.VI |
|  | ucqGD7-5 | 167 | Bmag900 | 166.5-167.5 |  | 7H | 167 | 5.46 | 3.04 | 0.08 | 166.5-167.5 | Y1.VII |

**Supplementary Table S3. Detailed information of conditional QTLs for grain filling rate and five grain size traits in DH population.**

| Trait | Consensus QTLs | | | |  | Identified QTLs | | | | | | |
| --- | --- | --- | --- | --- | --- | --- | --- | --- | --- | --- | --- | --- |
|  | Consensus QTLs | Peak | The closest marker | CI |  | Chr. | Position | LOD | R^2^ | Add | CI | Stages |
| GFR | cqGFR1-1 | 22 | M_1601731_2584 | 21.5-22.5 |  | 1H | 17 | 3.22 | 3.99 | -0.02 | 14.5-18.5 | Y1.ΔT2 |
|  | cqGFR1-2 | 54 | 1H_58188215 | 52.5-54.5 |  | 1H | 54 | 4.97 | 1.54 | -0.02 | 52.5-54.5 | Y2.ΔT2 |
|  |  |  |  |  |  | 1H | 54 | 3.27 | 5.44 | 0.08 | 52.5-54.5 | Y2.ΔT5 |
|  | cqGFR2-1 | 88 | Bmag829 | 87.35-88.24 |  | 2H | 88 | 3.95 | 3.18 | 0.05 | 87.5-88.5 | Y1.ΔT3 |
|  |  |  |  |  |  | 2H | 88 | 4.65 | 12.78 | -0.12 | 87.5-89.5 | Y1.ΔT5 |
|  |  |  |  |  |  | 2H | 88 | 3.74 | 1.26 | 0.05 | 87.5-89.5 | Y2.ΔT3 |
|  |  |  |  |  |  | 2H | 88 | 4.69 | 8.09 | -0.10 | 87.5-89.5 | Y2.ΔT5 |
|  | cqGFR2-2 | 126.83 | 2HL_22930005 | 126.45-127.25 |  | 2H | 123 | 6.90 | 19.91 | -0.07 | 122.5-123.5 | Y1.ΔT7 |
|  |  |  |  |  |  | 2H | 123 | 6.51 | 14.92 | -0.05 | 122.5-123.5 | Y2.ΔT7 |
|  |  |  |  |  |  | 2H | 127 | 26.30 | 52.18 | 0.08 | 126.5-127.5 | Y1.ΔT2 |
|  |  |  |  |  |  | 2H | 127 | 39.08 | 65.72 | 0.23 | 126.5-127.5 | Y1.ΔT3 |
|  |  |  |  |  |  | 2H | 127 | 27.59 | 61.52 | -0.28 | 126.5-127.5 | Y1.ΔT6 |
|  |  |  |  |  |  | 2H | 127 | 30.64 | 16.30 | 0.08 | 126.5-127.5 | Y2.ΔT2 |
|  |  |  |  |  |  | 2H | 127 | 36.73 | 24.67 | 0.22 | 126.5-127.5 | Y2.ΔT3 |
|  |  |  |  |  |  | 2H | 127 | 17.44 | 37.78 | 0.17 | 126.5-127.5 | Y2.ΔT4 |
|  |  |  |  |  |  | 2H | 127 | 14.58 | 30.36 | -0.21 | 126.5-127.5 | Y2.ΔT5 |
|  |  |  |  |  |  | 2H | 127 | 28.19 | 60.77 | -0.24 | 126.5-127.5 | Y2.ΔT6 |
|  |  |  |  |  |  | 2H | 129 | 20.18 | 40.39 | 0.22 | 128.5-129.5 | Y1.ΔT4 |
|  | cqGFR2-3 | 132 | 2HL_43143355 | 131.5-132.5 |  | 2H | 132 | 8.69 | 26.63 | -0.19 | 131.5-132.5 | Y1.ΔT5 |
|  | cqGFR3-1 | 28 | 3_525094736 | 27.64-28.35 |  | 3H | 27 | 4.88 | 14.46 | -0.06 | 26.5-27.5 | Y1.ΔT7 |
|  |  |  |  |  |  | 3H | 29 | 6.94 | 12.25 | 0.10 | 28.5-29.5 | Y2.ΔT4 |
|  | cqGFR3-2 | 33 | 3_511749149 | 32.66-33.33 |  | 3H | 33 | 6.25 | 5.29 | -0.07 | 32.5-34.5 | Y1.ΔT3 |
|  |  |  |  |  |  | 3H | 33 | 4.20 | 11.66 | 0.13 | 32.5-34.5 | Y1.ΔT5 |
|  |  |  |  |  |  | 3H | 33 | 5.35 | 1.73 | -0.03 | 32.5-34.5 | Y2.ΔT2 |
|  |  |  |  |  |  | 3H | 33 | 4.06 | 1.40 | -0.05 | 32.5-34.5 | Y2.ΔT3 |
|  |  |  |  |  |  | 3H | 33 | 9.28 | 17.75 | 0.16 | 32.5-34.5 | Y2.ΔT5 |
|  |  |  |  |  |  | 3H | 33 | 9.63 | 22.86 | -0.07 | 32.5-34.5 | Y2.ΔT7 |
|  | cqGFR5 | 0.03 | 5HS_7374618 | 0.21-0.28 |  | 5H | 4 | 3.68 | 5.35 | -0.07 | 0-5.5 | Y1.ΔT4 |
|  |  |  |  |  |  | 5H | 0 | 3.19 | 5.17 | -0.06 | 0-0.5 | Y2.ΔT4 |
|  | cqGFR7-1 | 58 | 1_4089724 | 57.5-58.5 |  | 7H | 58 | 5.06 | 7.49 | 0.09 | 57.8-58.5 | Y1.ΔT4 |
|  |  |  |  |  |  | 7H | 58 | 5.52 | 9.44 | 0.08 | 57.5-58.5 | Y2.ΔT4 |
|  | cqGFR7-2 | 65.5 | 7HL_37199773 | 65.25-65.75 |  | 7H | 65 | 5.75 | 8.46 | -0.09 | 64.5-65.5 | Y1.ΔT6 |
|  |  |  |  |  |  | 7H | 67 | 3.77 | 9.90 | -0.04 | 66.5-67.5 | Y1.ΔT7 |
|  |  |  |  |  |  | 7H | 67 | 5.55 | 11.91 | -0.04 | 66.5-67.5 | Y2.ΔT7 |
|  | cqGFR7-3 | 122 | 7HS_21726812 | 120.5-122.5 |  | 7H | 122 | 6.25 | 8.52 | -0.08 | 120.5-122.5 | Y2.ΔT6 |
|  | cqGFR7-4 | 133 | 7HS_17906516 | 132.5-134.5 |  | 7H | 133 | 8.66 | 16.27 | -0.11 | 132.5-134.5 | Y1.ΔT7 |
| GA | cqGA2-1 | 121 | 2_506545106 | 120.5-122.5 |  | 2H | 121 | 4.00 | 8.35 | 0.30 | 120.5-122.5 | Y1.ΔT5 |
|  | cqGA2-2 | 127.54 | 2HL_17075593 | 127.13-127.84 |  | 2H | 125 | 15.63 | 40.97 | 0.88 | 124.5-126.5 | Y1.ΔT4 |
|  |  |  |  |  |  | 2H | 127 | 9.25 | 27.71 | 0.85 | 126.5-127.5 | Y2.ΔT3 |
|  |  |  |  |  |  | 2H | 129 | 10.55 | 27.72 | 0.77 | 128.5-129.5 | Y2.ΔT4 |
|  | cqGA3-1 | 33.3 | 3_511749149 | 32.47-34.13 |  | 3H | 34 | 4.22 | 9.36 | 0.35 | 32.5-35.5 | Y1.ΔT6 |
|  |  |  |  |  |  | 3H | 33 | 3.12 | 6.79 | 0.23 | 32.5-34.5 | Y2.ΔT6 |
|  | cqGA3-2 | 41 | 3_499436820 | 39.93-42.06 |  | 3H | 41 | 5.06 | 10.48 | 0.32 | 38.5-41.5 | Y1.ΔT5 |
|  |  |  |  |  |  | 3H | 41 | 5.14 | 12.13 | 0.38 | 38.5-41.5 | Y2.ΔT5 |
|  | cqGA4 | 90 | 4HL_42790942 | 89.5-90.5 |  | 4H | 90 | 3.48 | 7.42 | -0.23 | 89.5-90.5 | Y2.ΔT6 |
|  | cqGA5-1 | 11 | 5HS_10560611 | 9.5-11.5 |  | 5H | 11 | 4.79 | 9.98 | -0.30 | 9.5-11.5 | Y1.ΔT5 |
|  | cqGA5-2 | 203 | 5_226253827 | 202.5-203.5 |  | 5H | 203 | 3.74 | 8.59 | -0.31 | 202.5-203.5 | Y2.ΔT5 |
|  | cqGA7-1 | 58 | 1_4089724 | 57.5-58.5 |  | 7H | 58 | 4.78 | 11.32 | 0.46 | 57.5-58.5 | Y2.ΔT4 |
|  | cqGA7-2 | 65 | 7HL_8312277 | 64.5-65.5 |  | 7H | 65 | 7.85 | 27.58 | 0.22 | 64.5-65.5 | Y2.ΔT7 |
|  | cqGA7-3 | 97 | 7_440111505 | 96.5-97.5 |  | 7H | 97 | 7.54 | 18.97 | 0.46 | 96.5-97.5 | Y2.ΔT5 |
|  | cqGA7-4 | 165 | 7_194302352 | 164.29-165.7 |  | 7H | 165 | 3.22 | 11.45 | 0.42 | 163.5-165.5 | Y1.ΔT3 |
|  |  |  |  |  |  | 7H | 165 | 4.48 | 12.31 | 0.52 | 163.5-165.5 | Y2.ΔT3 |
| GP | cqGP7-1 | 57 | 1_4088556 | 56.5-57.5 |  | 7H | 57 | 3.50 | 14.30 | 0.52 | 56.5-57.5 | Y2.ΔT3 |
|  | cqGP7-2 | 68.99 | 7HL_3360534 | 68.64-69.35 |  | 7H | 68 | 5.01 | 17.05 | 0.20 | 67.5-68.5 | Y2.ΔT6 |
|  |  |  |  |  |  | 7H | 70 | 13.78 | 41.39 | 0.35 | 69.5-70.5 | Y2.ΔT7 |
|  | cqGP7-3 | 124 | 7HS_32890650 | 123.5-124.5 |  | 7H | 124 | 25.21 | 16.88 | 1.57 | 123.5-124.5 | Y1.ΔT3 |
|  | cqGP7-4 | 153 | M_90598_706 | 152.5-153.5 |  | 7H | 153 | 18.28 | 10.41 | -1.22 | 152.5-153.5 | Y1.ΔT3 |
| GL | cqGL1-1 | 19 | 1H_17392124 | 18.64-19.35 |  | 1H | 19 | 3.02 | 11.34 | 0.21 | 18.5-19.5 | Y1.ΔT3 |
|  |  |  |  |  |  | 1H | 19 | 8.28 | 14.00 | 0.32 | 18.5-19.5 | Y2.ΔT3 |
|  | cqGL1-2 | 114 | 1H_34522869 | 113.5-115.5 |  | 1H | 114 | 5.68 | 9.06 | -0.26 | 113.5-115.5 | Y2.ΔT3 |
|  | cqGL3 | 19 | 3HL_42780152 | 17.5-19.5 |  | 3H | 19 | 4.61 | 7.29 | 0.24 | 17.5-19.5 | Y2.ΔT3 |
|  | cqGL7-1 | 66 | 7HL_4313756 | 65.71-66.28 |  | 7H | 66 | 4.22 | 16.21 | 0.09 | 65.5-66.5 | Y1.ΔT6 |
|  |  |  |  |  |  | 7H | 65 | 5.72 | 9.08 | 0.26 | 64.5-65.5 | Y2.ΔT3 |
|  |  |  |  |  |  | 7H | 67 | 8.07 | 27.07 | 0.11 | 66.5-67.5 | Y2.ΔT5 |
|  | cqGL7-2 | 92.5 | 7HL_27996637 | 92.14-92.85 |  | 7H | 94 | 4.37 | 12.70 | 0.06 | 93.5-94.5 | Y1.ΔT7 |
|  |  |  |  |  |  | 7H | 91 | 12.36 | 38.03 | 0.11 | 90.5-91.5 | Y2.ΔT7 |
|  | cqGL7-3 | 109 | 7HS_35580690 | 108.5-110.5 |  | 7H | 109 | 3.22 | 12.19 | -0.11 | 108.5-110.5 | Y2.ΔT4 |
|  | cqGL7-4 | 124 | 7HS_32890650 | 123.5-124.5 |  | 7H | 124 | 3.20 | 12.31 | 0.22 | 123.5-124.5 | Y1.ΔT3 |
| GW | cqGW2-1 | 53 | 2HL_17013042 | 52.5-53.5 |  | 2H | 53 | 4.08 | 15.00 | -0.06 | 52.5-53.5 | Y1.ΔT6 |
|  | cqGW2-2 | 86 | M_207663_1931 | 85.5-86.5 |  | 2H | 86 | 3.82 | 12.43 | -0.05 | 85.5-86.5 | Y2.ΔT6 |
|  | cqGW2-3 | 116 | 2HL_13832944 | 115.5-116.5 |  | 2H | 116 | 5.00 | 18.33 | 0.06 | 115.5-116.5 | Y2.ΔT5 |
|  | cqGW2-4 | 126 | 2_527241334 | 125.75-126.25 |  | 2H | 127 | 4.29 | 13.33 | 0.07 | 126.5-127.5 | Y1.ΔT3 |
|  |  |  |  |  |  | 2H | 126 | 14.95 | 36.76 | 0.16 | 125.5-126.5 | Y1.ΔT4 |
|  |  |  |  |  |  | 2H | 127 | 5.72 | 18.68 | 0.05 | 126.5-127.5 | Y2.ΔT3 |
|  |  |  |  |  |  | 2H | 124 | 23.87 | 55.68 | 0.15 | 123.5-124.5 | Y2.ΔT4 |
|  | cqGW3-1 | 27.5 | 3HL_38740410 | 27.14-27.85 |  | 3H | 27 | 4.08 | 13.72 | 0.06 | 26.5-27.5 | Y1.ΔT5 |
|  |  |  |  |  |  | 3H | 28 | 3.09 | 9.85 | -0.04 | 27.5-28.5 | Y2.ΔT3 |
|  | cqGW3-2 | 32.19 | 3HL_32354397 | 31.75-32.64 |  | 3H | 32 | 6.31 | 20.59 | -0.09 | 31.5-32.5 | Y1.ΔT3 |
|  |  |  |  |  |  | 3H | 33 | 4.74 | 16.11 | 0.06 | 32.5-34.5 | Y2.ΔT6 |
|  | cqGW3-3 | 83 | 3HL_3720522 | 82.5-83.5 |  | 3H | 83 | 3.07 | 10.93 | -0.05 | 82.5-83.5 | Y1.ΔT6 |
|  | cqGW3-4 | 98 | 3_219109045 | 96.5-98.5 |  | 3H | 98 | 4.17 | 15.39 | -0.05 | 96.5-98.5 | Y2.ΔT5 |
|  | cqGW5 | 4 | 5_3871196 | 1.5-5.5 |  | 5H | 4 | 3.71 | 11.83 | -0.05 | 1.5-5.5 | Y1.ΔT5 |
|  | cqGW6 | 32 | 6_518728726 | 31.5-33.5 |  | 6H | 32 | 3.22 | 11.98 | -0.04 | 31.5-33.5 | Y2.ΔT7 |
|  | cqGW7 | 130 | 7HS_25905506 | 129.5-130.5 |  | 7H | 130 | 3.93 | 7.76 | 0.07 | 129.5-130.5 | Y1.ΔT4 |
| GD | cqGD2-1 | 96 | 2HL_43859802 | 95.5-96.5 |  | 2H | 96 | 4.24 | 13.74 | 0.11 | 95.5-96.5 | Y1.ΔT3 |
|  | cqGD2-2 | 129 | 2HL_34260490 | 128.64-129.35 |  | 2H | 129 | 6.11 | 18.55 | 0.10 | 128.5-129.5 | Y1.ΔT4 |
|  |  |  |  |  |  | 2H | 129 | 6.05 | 20.41 | 0.11 | 128.5-129.5 | Y2.ΔT4 |
|  | cqGD3 | 27 | 3_529115904 | 26.5-27.5 |  | 3H | 27 | 3.71 | 10.91 | 0.05 | 26.5-27.5 | Y1.ΔT5 |
|  | cqGD4 | 152 | 4_16819133 | 151.5-154.5 |  | 4H | 152 | 3.31 | 9.05 | -0.04 | 151.5-154.5 | Y1.ΔT7 |
|  | cqGD5-1 | 9 | 5_51657943 | 8.5-10.5 |  | 5H | 9 | 3.04 | 9.30 | 0.04 | 8.5-10.5 | Y1.ΔT7 |
|  | cqGD5-2 | 17 | GBM1176 | 11.5-24.5 |  | 5H | 17 | 3.08 | 9.62 | -0.04 | 11.5-24.5 | Y1.ΔT5 |
|  | cqGD7-1 | 57.6 | 7_523855164 | 57.15-58.04 |  | 7H | 60 | 3.36 | 10.74 | 0.09 | 58.5-60.5 | Y1.ΔT3 |
|  |  |  |  |  |  | 7H | 57 | 3.14 | 12.89 | 0.09 | 56.5-57.5 | Y2.ΔT3 |
|  | cqGD7-2 | 70 | M_363857_407 | 69.5-70.5 |  | 7H | 70 | 10.99 | 34.69 | 0.09 | 69.5-70.5 | Y2.ΔT7 |
|  | cqGD7-3 | 80 | GMS46 | 78.5-80.5 |  | 7H | 80 | 6.18 | 17.99 | 0.06 | 78.5-80.5 | Y1.ΔT7 |

**Table S4. Covariate consensus QTLs for grain filling rate and five grain size traits identified by genome-wide composite interval mapping (GCIM).**

| **Trait** | **Consensus QTLs** | **Chr.** | **Covariate** | **Peak** | **The closest**  **marker** | **LOD** | **R^2^** | **Add** | **Stage** | **Unconditional**  **consensus QTLs** | **Conditional**  **consensus QTLs** |
| --- | --- | --- | --- | --- | --- | --- | --- | --- | --- | --- | --- |
| GA | qcGA1-1 | 1H | Rt | 18.14 | 1H_37679977 | 4.31-4.80 | 2.32-2.43 | -0.43 to -0.39 | Y1.IV/Y2.IV |  |  |
|  | qcGA1-2 | 1H | Rt | 132.59 | 1H_55583778 | 5.27 | 1.80 | -0.51 | Y2.VII |  |  |
|  | qcGA1-1 | 1H | Ct | 17.50 | 1_304812961 | 3.21 | 2.15 | -0.41 | Y2.IV |  |  |
|  | qcGA1-2 | 1H | Ct | 20.63 | 1_306385557 | 3.88-4.37 | 1.86-9.03 | -0.43 to -0.38 | Y1.II,IV,V | ucqGA1-1 |  |
|  | qcGA1-1 | 1H | Rt+Ct | 17.50 | 1_304812961 | 4.56 | 2.39 | -0.43 | Y2.IV |  |  |
|  | qcGA1-2 | 1H | Rt+Ct | 20.63 | 1_306385557 | 3.93-4.38 | 2.25-9.47 | -0.43 to -0.39 | Y1.II,IV | ucqGA1-1 |  |
|  | qcGA1-3 | 1H | Rt+Ct | 132.59 | 1H_55583778 | 3.28 | 1.10 | -0.40 | Y2.VII | ucqGA1-5 |  |
|  | qcGA2-1 | 2H | Rt | 124.44 | 2HL_7395625 | 4.90 | 41.56 | 2.26 | Y2.VI | ucqGA2 | cqGA2-2 |
|  | qcGA2-2 | 2H | Rt | 146.44 | 2HL_20046952 | 4.71 | 16.64 | 0.48 | Y1.ΔT5 |  |  |
|  | qcGA2-3 | 2H | Rt | 184.64 | 2_598551866 | 4.71 | 6.10 | -0.29 | Y1.ΔT5 |  |  |
|  | qcGA2-1 | 2H | Ct | 46.63 | Bmag518 | 3.89 | 1.86 | 0.45 | Y1.VII |  |  |
|  | qcGA2-2 | 2H | Ct | 95.12 | M_54203_5996 | 3.04 | 2.12 | 0.42 | Y1.V |  |  |
|  | qcGA2-3 | 2H | Ct | 126.73 | 2HL_22930005 | 3.17-49.18 | 12.39-68.98 | 0.30-2.89 | Y1.II,III,IV,V,VI,VII,ΔT3,ΔT4,ΔT6 | ucqGA2 | cqGA2-2 |
|  |  |  |  |  |  |  |  |  | Y2.III,IV,V,VI,VII,ΔT3,ΔT4 |  |  |
|  | qcGA2-4 | 2H | Ct | 133.61 | M_149956_1482 | 6.99 | 24.93 | 0.67 | Y2.II |  |  |
|  | qcGA2-5 | 2H | Ct | 137.73 | 1H_83810983 | 3.25 | 3.98 | 0.66 | Y1.VII |  |  |
|  | qcGA2-6 | 2H | Ct | 146.44 | 2HL_20046952 | 4.49 | 9.47 | 0.34 | Y1.ΔT5 |  |  |
|  | qcGA2-7 | 2H | Ct | 184.64 | 2_598551866 | 3.23 | 4.11 | -0.22 | Y1.ΔT5 |  |  |
|  | qcGA2-1 | 2H | Rt+Ct | 124.44 | 2HL_7395625 | 3.84 | 43.91 | 2.32 | Y2.VI | ucqGA2 | cqGA2-2 |
|  | qcGA2-2 | 2H | Rt+Ct | 146.44 | 2HL_20046952 | 4.57 | 16.14 | 0.46 | Y1.ΔT5 |  |  |
|  | qcGA2-3 | 2H | Rt+Ct | 184.64 | 2_598551866 | 4.38 | 5.66 | -0.27 | Y1.ΔT5 |  |  |
|  | qcGA3-1 | 3H | Rt | 35.11 | 3_511749149 | 5.76 | 2.50 | 0.61 | Y2.VII |  | cqGA3-1 |
|  | qcGA3-2 | 3H | Rt | 41.41 | 3_499436820 | 4.31-5.50 | 10.44-12.03 | 0.37-0.38 | Y1.ΔT5/Y2.ΔT5 |  | cqGA3-2 |
|  | qcGA3-3 | 3H | Rt | 57.33 | 3HL_24669457 | 3.25-3.35 | 1.79-2.10 | 0.43-0.48 | Y1.VI,VII | ucqGA3-2 |  |
|  | qcGA3-4 | 3H | Rt | 65.59 | 3_452489880 | 4.02 | 6.86 | -0.31 | Y1.ΔT5 |  |  |
|  | qcGA3-5 | 3H | Rt | 83.78 | 3_403275941 | 7.91 | 5.89 | -0.80 | Y1.VII |  |  |
|  | qcGA3-6 | 3H | Rt | 91.07 | 3_267212934 | 5.03 | 3.49 | -0.61 | Y1.VI | ucqGA3-3 |  |
|  | qcGA3-7 | 3H | Rt | 149.04 | 3HL_46409485 | 4.79 | 2.53 | -0.46 | Y1.V |  |  |
|  | qcGA3-8 | 3H | Rt | 157.23 | 3_327391418 | 7.03 | 2.41 | -0.60 | Y2.VII |  |  |
|  | qcGA3-1 | 3H | Ct | 32.64 | 3_504148850 | 3.44-4.67 | 2.39-11.39 | -0.49 to 0.59 | Y1.III,ΔT6/Y2.VII,ΔT6 | ucqGA3-1 | cqGA3-1 |
|  | qcGA3-2 | 3H | Ct | 41.41 | 3_499436820 | 3.61-3.68 | 6.86-10.76 | 0.29-0.34 | Y1.ΔT5/Y2.ΔT5 |  | cqGA3-2 |
|  | qcGA3-3 | 3H | Ct | 56.14 | 3_474974240 | 6.29-7.55 | 3.24-3.72 | 0.56-0.59 | Y1.V,VII | ucqGA3-2 |  |
|  | qcGA3-4 | 3H | Ct | 65.59 | 3_452489880 | 4.88 | 6.62 | -0.28 | Y2.ΔT5 |  |  |
|  | qcGA3-5 | 3H | Ct | 71.40 | 3_434559804 | 7.53 | 4.35 | -0.60 | Y1.V |  |  |
|  | qcGA3-6 | 3H | Ct | 84.34 | 3HL_26328866 | 9.20 | 5.78 | -0.79 | Y1.VII |  |  |
|  | qcGA3-7 | 3H | Ct | 147.31 | 3HL_17901650 | 3.52 | 1.51 | -0.47 | Y2.VII |  |  |
|  | qcGA3-8 | 3H | Ct | 183.63 | 3_426707684 | 3.17 | 1.23 | -0.48 | Y1.VI |  |  |
|  | qcGA3-1 | 3H | Rt+Ct | 33.13 | 3_511749149 | 3.68-5.48 | 2.47-11.72 | 0.26-0.60 | Y1.ΔT6/Y2.VII,ΔT6 | ucqGA3-1 | cqGA3-1 |
|  | qcGA3-2 | 3H | Rt+Ct | 41.41 | 3_499436820 | 4.95-5.86 | 11.21-11.54 | 0.36-0.38 | Y1.ΔT5/Y2.ΔT5 |  | cqGA3-2 |
|  | qcGA3-3 | 3H | Rt+Ct | 57.23 | 3HL_34537138 | 3.23-3.89 | 1.41-2.06 | 0.39-0.47 | Y1.VI,VII | ucqGA3-2 |  |
|  | qcGA3-4 | 3H | Rt+Ct | 65.59 | 3_452489880 | 3.53 | 6.11 | -0.28 | Y1.ΔT5 |  |  |
|  | qcGA3-5 | 3H | Rt+Ct | 91.07 | 3_267212934 | 4.33-7.83 | 3.03-5.62 | -0.77 to -0.57 | Y1.VI,VII | ucqGA3-3 |  |
|  | qcGA3-6 | 3H | Rt+Ct | 147.31 | 3HL_17901650 | 4.67 | 1.60 | -0.49 | Y2.VII |  |  |
|  | qcGA3-7 | 3H | Rt+Ct | 157.23 | 3_327391418 | 4.80 | 2.53 | -0.46 | Y1.V |  |  |
|  | qcGA4 | 4H | Rt | 20.90 | 4HL_42250692 | 3.46 | 0.75 | -0.33 | Y2.VII |  |  |
|  | qcGA4-1 | 4H | Ct | 66.16 | 4HL_44314448 | 3.20 | 1.08 | -0.45 | Y1.VI |  |  |
|  | qcGA4-2 | 4H | Ct | 72.19 | Bmac30 | 4.72 | 2.27 | -0.50 | Y1.VII |  |  |
|  | qcGA5-1 | 5H | Rt | 10.82 | 5HS_10560611 | 4.97 | 8.05 | -0.33 | Y1.ΔT5 |  | cqGA5-1 |
|  | qcGA5-2 | 5H | Rt | 145.85 | M_322077_4294967285 | 4.25 | 1.77 | -0.43 | Y1.VI |  |  |
|  | qcGA5-3 | 5H | Rt | 218.60 | 5_464856478 | 4.16 | 4.69 | -0.25 | Y1.ΔT5 |  |  |
|  | qcGA5-1 | 5H | Ct | 10.82 | 5HS_10560611 | 4.33 | 7.88 | -0.31 | Y1.ΔT5 |  | cqGA5-1 |
|  | qcGA5-2 | 5H | Ct | 139.72 | 1H_81979810 | 3.89 | 0.83 | -0.40 | Y1.VI |  |  |
|  | qcGA5-3 | 5H | Ct | 203.25 | 5_436205970 | 3.19-3.47 | 1.34-7.55 | -0.38 to -0.29 | Y1.VII/Y2.ΔT5 |  | cqGA5-2 |
|  | qcGA5-4 | 5H | Ct | 218.60 | 5_464856478 | 4.13 | 5.27 | -0.25 | Y1.ΔT5 |  |  |
|  | qcGA5-1 | 5H | Rt+Ct | 10.82 | 5HS_10560611 | 5.09 | 8.26 | -0.33 | Y1.ΔT5 |  | cqGA5-1 |
|  | qcGA5-2 | 5H | Rt+Ct | 203.25 | 5_436205970 | 3.59 | 1.70 | -0.42 | Y1.VI |  | cqGA5-2 |
|  | qcGA5-3 | 5H | Rt+Ct | 218.60 | 5_464856478 | 4.67 | 5.32 | -0.26 | Y1.ΔT5 |  |  |
|  | qcGA6 | 6H | Rt | 2.74 | 1H_62545792 | 3.25-3.56 | 1.68-1.74 | -0.42 | Y1.VI,VII |  |  |
|  | qcGA7-1 | 7H | Rt | 59.41 | 7HL_33935949 | 3.92-5.86 | 3.22-10.14 | 0.44-0.71 | Y1.IV/Y2.IV,ΔT4 |  | cqGA7-1 |
|  | qcGA7-2 | 7H | Rt | 66.14 | 7HL_4313756 | 8.19-15.11 | 14.29-25.72 | 0.54-1.53 | Y2.VI,VII,ΔT5 | ucqGA7-1 | cqGA7-2 |
|  | qcGA7-3 | 7H | Rt | 72.57 | 7_486000975 | 3.81-15.99 | 7.75-25.13 | 0.22-1.09 | Y1.VI,VII/Y2.III,V,ΔT7 |  |  |
|  | qcGA7-4 | 7H | Rt | 92.90 | 7HL_39750192 | 12.61 | 36.31 | 0.51 | Y2.ΔT6 | ucqGA7-2 |  |
|  | qcGA7-5 | 7H | Rt | 115.22 | 7HL_31175469 | 11.68 | 10.45 | 0.94 | Y1.V |  |  |
|  | qcGA7-6 | 7H | Rt | 118.25 | 7_262149691 | 6.33 | 6.88 | 0.73 | Y2.IV |  |  |
|  | qcGA7-7 | 7H | Rt | 151.09 | Bmac31 | 4.66-6.71 | 6.06-8.98 | 0.91-0.94 | Y2.V,VI,VII | ucqGA7-3 |  |
|  | qcGA7-8 | 7H | Rt | 172.81 | 7_180156097 | 5.51 | 4.53 | 0.55 | Y1.IV |  |  |
|  | qcGA7-1 | 7H | Ct | 40.40 | 7_549668049 | 4.03 | 2.40 | 0.45 | Y1.V |  |  |
|  | qcGA7-2 | 7H | Ct | 56.59 | 1_4088556 | 3.53 | 5.05 | 0.62 | Y2.IV |  | cqGA7-1 |
|  | qcGA7-3 | 7H | Ct | 179.63 | 7HS_24633706 | 3.26-6.02 | 3.22-7.19 | 0.49-0.69 | Y1.III,IV/Y2.III,V,VII |  |  |
|  | qcGA7-1 | 7H | Rt+Ct | 40.40 | 7_549668049 | 3.10 | 1.73 | 0.38 | Y1.V |  |  |
|  | qcGA7-2 | 7H | Rt+Ct | 115.53 | 7HL_31175469 | 3.90-4.10 | 4.68-7.94 | 0.63-0.78 | Y1.V/Y2.IV |  |  |
|  | qcGA7-3 | 7H | Rt+Ct | 163.92 | M_262022_735 | 7.84 | 5.08 | 0.86 | Y1.IV |  | cqGA7-4 |
|  | qcGA7-4 | 7H | Rt+Ct | 172.81 | 7_180156097 | 3.68 | 3.71 | 0.50 | Y2.VII |  |  |
| GP | qcGP1 | 1H | Ct | 20.63 | 1_306385557 | 3.47 | 9.42 | -0.52 | Y1.II | ucqGP1-1 |  |
|  | qcGP2-1 | 2H | Ct | 125.53 | 2HL_22930294 | 3.46-35.74 | 21.89-68.01 | 0.85-1.14 | Y1.III,IV,V,VI,VII | ucqGP2-1 |  |
|  |  |  |  |  |  |  |  |  | Y2.IV,V,VI,VII |  |  |
|  | qcGP2-2 | 2H | Ct | 133.61 | M_149956_1482 | 5.93-7.56 | 20.26-24.57 | 0.79-0.84 | Y1.II/Y2.II | ucqGP2-2 |  |
|  | qcGP3-1 | 3H | Rt | 57.14 | 3HL_34537138 | 5.22-5.33 | 6.72-7.09 | 0.35-0.39 | Y1.VI,VII | ucqGP3-1 |  |
|  | qcGP3-2 | 3H | Rt | 93.76 | 3HL_6015573 | 3.01 | 2.12 | -0.24 | Y2.IV | ucqGP3-2 |  |
|  | qcGP3-3 | 3H | Rt | 175.76 | 3HL_25942927 | 6.15-6.54 | 9.02-9.49 | -0.45 to -0.41 | Y1.VI,VII |  |  |
|  | qcGP3-1 | 3H | Ct | 55.41 | 3HL_48064911 | 3.83-5.85 | 1.42-8.54 | 0.27-0.43 | Y1.VI,VII/Y2.V,VII | ucqGP3-1 |  |
|  | qcGP3-2 | 3H | Ct | 87.87 | 6HL_25349295 | 3.35-5.84 | 1.05-3.85 | -0.34 to -0.23 | Y2.V,VII |  |  |
|  | qcGP3-3 | 3H | Ct | 158.56 | M_1876186_1745 | 5.15 | 6.59 | -0.35 | Y1.VI |  |  |
|  | qcGP3-4 | 3H | Ct | 175.76 | 3HL_25942927 | 4.94 | 7.45 | -0.40 | Y1.VII |  |  |
|  | qcGP3-1 | 3H | Rt+Ct | 48.92 | 3_482018807 | 3.35-5.23 | 1.24-6.29 | 0.25-0.37 | Y1.VII/Y2.VII |  |  |
|  | qcGP3-2 | 3H | Rt+Ct | 55.41 | 3HL_48064911 | 6.03 | 7.88 | 0.38 | Y1.VI | ucqGP3-1 |  |
|  | qcGP3-3 | 3H | Rt+Ct | 87.99 | 3HL_19389223 | 3.39-3.42 | 1.02-1.86 | -0.23 | Y2.V,VII |  |  |
|  | qcGP3-4 | 3H | Rt+Ct | 175.76 | 3HL_25942927 | 3.86-5.89 | 5.19-8.28 | -0.39 to 0.33 | Y1.VI,VII |  |  |
|  | qcGP5 | 5H | Rt | 1.75 | 5_54803 | 3.06 | 8.13 | 0.50 | Y2.II |  |  |
|  | qcGP5 | 5H | Ct | 203.25 | 5_436205970 | 3.34 | 1.67 | -0.22 | Y2.V | ucqGP5 |  |
|  | qcGP5-1 | 5H | Rt+Ct | 1.75 | 5_54803 | 3.01 | 8.09 | 0.50 | Y2.II |  |  |
|  | qcGP5-2 | 5H | Rt+Ct | 203.25 | 5_436205970 | 3.07-3.68 | 1.12-1.87 | -0.24 | Y2.V,VII | ucqGP5 |  |
|  | qcGP6 | 6H | Rt | 102.03 | M_1661027_233 | 3.43 | 2.16 | 0.29 | Y2.VI | ucqGP6 |  |
|  | qcGP6 | 6H | Ct | 84.71 | 2HS_26945700 | 3.10 | 2.45 | 0.31 | Y2.VI |  |  |
|  | qcGP7-1 | 7H | Rt | 49.95 | GBM1102 | 3.18 | 4.46 | 0.34 | Y2.III | ucqGP7-1 |  |
|  | qcGP7-2 | 7H | Rt | 66.67 | 7_501748124 | 4.85-17.00 | 9.30-32.03 | 0.20-1.27 | Y2.IV,V,VI,VII,ΔT6 | ucqGP7-2 | cqGP7-2 |
|  | qcGP7-3 | 7H | Rt | 70.04 | M_363857_407 | 13.78 | 41.38 | 0.35 | Y2.ΔT7 |  |  |
|  | qcGP7-4 | 7H | Rt | 73.63 | 7_489795725 | 3.70 | 5.01 | 0.30 | Y1.V |  |  |
|  | qcGP7-5 | 7H | Rt | 118.25 | 7_262149691 | 4.78-16.43 | 12.52-18.01 | 0.56-0.62 | Y1.IV/Y2.II | ucqGP7-4 |  |
|  | qcGP7-6 | 7H | Rt | 136.21 | 7HS_29196961 | 3.74 | 14.15 | -0.25 | Y1.ΔT4 | ucqGP7-5 |  |
|  | qcGP7-7 | 7H | Rt | 142.42 | 7HS_4123979 | 11.22 | 13.03 | 0.49 | Y1.VI | ucqGP7-6 |  |
|  | qcGP7-8 | 7H | Rt | 151.09 | Bmac31 | 5.48-22.07 | 8.48-30.50 | 0.65-0.79 | Y1.III/Y2.IV,V,VI,VII | ucqGP7-7 | cqGP7-4 |
|  | qcGP7-9 | 7H | Rt | 165.59 | 7HS_14000381 | 17.39 | 30.04 | 0.87 | Y2.III |  |  |
|  | qcGP7-10 | 7H | Rt | 181.04 | 7HS_7499702 | 4.12-6.95 | 4.58-7.08 | 0.29-0.39 | Y1.V,VII |  |  |
|  | qcGP7-11 | 7H | Rt | 226.61 | 7_5539511 | 3.26 | 2.84 | -0.23 | Y1.V |  |  |
|  | qcGP7-1 | 7H | Ct | 123.44 | 7_311871132 | 5.74-10.29 | 6.51-25.35 | 0.54-0.74 | Y1.III/Y2.IV,V,VII |  | cqGP7-3 |
|  | qcGP7-2 | 7H | Ct | 130.18 | 7HS_35889676 | 6.75 | 12.56 | 0.49 | Y1.IV |  |  |
|  | qcGP7-3 | 7H | Ct | 139.84 | 7_319506952 | 10.79 | 21.72 | 0.74 | Y2.III | ucqGP7-6 |  |
|  | qcGP7-4 | 7H | Ct | 181.04 | 7HS_7499702 | 3.94-4.09 | 5.65-5.80 | 0.32 | Y1.V,VI |  |  |
|  | qcGP7-1 | 7H | Rt+Ct | 115.22 | 7HL_31175469 | 6.93 | 11.32 | 0.58 | Y2.V | ucqGP7-4 |  |
|  | qcGP7-2 | 7H | Rt+Ct | 123.44 | 7_311871132 | 6.08-11.18 | 6.63-28.38 | 0.58-0.76 | Y1.III/Y2.IV,VII |  | cqGP7-3 |
|  | qcGP7-3 | 7H | Rt+Ct | 139.84 | 7_319506952 | 11.33 | 22.10 | 0.75 | Y2.III | ucqGP7-6 |  |
|  | qcGP7-4 | 7H | Rt+Ct | 167.94 | 7_146904388 | 5.56 | 9.60 | 0.41 | Y1.IV |  |  |
|  | qcGP7-5 | 7H | Rt+Ct | 181.04 | 7HS_7499702 | 4.20-4.54 | 5.62-5.87 | 0.32 | Y1.V,VI |  |  |
| GL | qcGL1 | 1H | Rt | 18.81 | 1H_17392124 | 3.04-3.40 | 11.05-12.35 | 0.23-0.25 | Y1.ΔT3/Y2.ΔT3 |  | cqGL1-1 |
|  | qcGL1 | 1H | Ct | 18.81 | 1H_17392124 | 3.13 | 10.44 | 0.23 | Y2.ΔT3 |  | cqGL1-1 |
|  | qcGL1 | 1H | Rt+Ct | 18.81 | 1H_17392124 | 3.13 | 10.36 | 0.23 | Y2.ΔT3 |  | cqGL1-1 |
|  | qcGL2 | 2H | Rt | 142.99 | 2HL_23067013 | 3.71-4.07 | 2.69-5.11 | 0.15-0.19 | Y2.VI,VII |  |  |
|  | qcGL2-1 | 2H | Ct | 126.13 | 2_527241334 | 6.59-26.00 | 15.47-46.05 | 0.35-0.44 | Y1.III,IV,V,VII | ucqGL2-1 |  |
|  |  |  |  |  |  |  |  |  | Y2.III,IV,V,VI,VII |  |  |
|  | qcGL2-2 | 2H | Ct | 130.36 | 2_524782265 | 11.20 | 25.79 | 0.36 | Y1.VI |  |  |
|  | qcGL2-3 | 2H | Ct | 133.61 | M_149956_1482 | 4.52-5.19 | 16.93-17.26 | 0.33-0.36 | Y1.II/Y2.II | ucqGL2-2 |  |
|  | qcGL2 | 2H | Rt+Ct | 172.55 | GBM1149 | 3.05 | 1.24 | 0.10 | Y2.VI |  |  |
|  | qcGL3-1 | 3H | Rt | 18.89 | 3HL_42780152 | 3.23-3.50 | 9.42-10.75 | -0.26 | Y1.II/Y2.II |  | cqGL3 |
|  | qcGL3-2 | 3H | Rt | 48.92 | 3_482018807 | 4.67-6.53 | 0.79-5.24 | 0.08-0.15 | Y2.IV,VI,VII | ucqGL3-1 |  |
|  | qcGL3-3 | 3H | Rt | 66.06 | 3HL_29437708 | 4.48 | 11.47 | -0.08 | Y2.ΔT5 |  |  |
|  | qcGL3-4 | 3H | Rt | 111.92 | 3_252652374 | 5.30 | 11.73 | 0.32 | Y2.VII |  |  |
|  | qcGL3-5 | 3H | Rt | 157.23 | 3_327391418 | 7.07 | 16.62 | -0.38 | Y2.VII |  |  |
|  | qcGL3-1 | 3H | Ct | 48.92 | 3_482018807 | 4.08-7.75 | 1.52-7.93 | 0.10-0.18 | Y1.V/Y2.IV,V,VI,VII | ucqGL3-1 |  |
|  | qcGL3-2 | 3H | Ct | 66.06 | 3HL_29437708 | 3.48 | 4.95 | -0.15 | Y1.V |  |  |
|  | qcGL3-1 | 3H | Rt+Ct | 18.89 | 3HL_42780152 | 3.22 | 10.30 | -0.26 | Y1.II |  | cqGL3 |
|  | qcGL3-2 | 3H | Rt+Ct | 48.92 | 3_482018807 | 3.97-8.65 | 1.56-5.72 | 0.10-0.16 | Y2.IV,V,VI,VII | ucqGL3-1 |  |
|  | qcGL3-3 | 3H | Rt+Ct | 147.31 | 3HL_17901650 | 3.60 | 0.94 | -0.09 | Y2.VII |  |  |
|  | qcGL5 | 5H | Ct | 102.19 | 5HS_16446198 | 3.58 | 0.97 | -0.09 | Y2.VII |  |  |
|  | qcGL5 | 5H | Rt+Ct | 102.19 | 5HS_16446198 | 3.73 | 1.03 | -0.10 | Y2.VII |  |  |
|  | qcGL7-1 | 7H | Rt | 57.85 | 7_524132075 | 7.68 | 7.18 | 0.20 | Y2.V |  |  |
|  | qcGL7-2 | 7H | Rt | 66.67 | 7_501748124 | 4.36-24.68 | 15.43-39.65 | 0.09-0.54 | Y1.ΔT6/Y2.VI,VII,ΔT5,ΔT6 | ucqGL7-1 | cqGL7-1 |
|  | qcGL7-3 | 7H | Rt | 93.97 | Bmag746 | 4.19-13.23 | 3.74-37.88 | 0.11-0.33 | Y2.II,IV,V,VI,VII,ΔT7 |  | cqGL7-2 |
|  | qcGL7-4 | 7H | Rt | 110.15 | M_310867_507 | 3.88 | 9.78 | 0.21 | Y2.IV |  | cqGL7-3 |
|  | qcGL7-5 | 7H | Rt | 116.44 | M_254133_378 | 22.32 | 37.53 | 0.40 | Y1.III | ucqGL7-2 |  |
|  | qcGL7-6 | 7H | Rt | 123.33 | 7_394187342 | 5.66 | 35.23 | 0.34 | Y1.IV |  | cqGL7-4 |
|  | qcGL7-7 | 7H | Rt | 135.24 | 7_382544975 | 19.27-26.67 | 36.57-47.29 | 0.37-0.55 | Y1.V/Y2.III | ucqGL7-3 |  |
|  | qcGL7-8 | 7H | Rt | 151.09 | Bmac31 | 19.48-20.15 | 42.39-43.09 | 0.46-0.52 | Y1.VI,VII | ucqGL7-5 |  |
|  | qcGL7-9 | 7H | Rt | 166.29 | Bmag571 | 5.20 | 7.87 | 0.21 | Y2.V |  |  |
|  | qcGL7-10 | 7H | Rt | 179.63 | 7HS_24633706 | 5.41-6.55 | 3.12-3.86 | 0.17 | Y2.VI,VII |  |  |
|  | qcGL7-1 | 7H | Ct | 123.44 | 7_311871132 | 5.74-10.51 | 6.51-31.13 | 0.37-0.57 | Y1.III/Y2.VII |  | cqGL7-4 |
|  | qcGL7-2 | 7H | Ct | 130.18 | 7HS_35889676 | 10.69 | 26.38 | 0.29 | Y1.IV |  |  |
|  | qcGL7-3 | 7H | Ct | 143.01 | 7HL_19514020 | 3.51-8.48 | 8.65-18.66 | 0.22-0.34 | Y2.III,IV,VI |  |  |
|  | qcGL7-4 | 7H | Ct | 179.13 | M_221260_990 | 8.72-8.85 | 4.11-5.12 | 0.17-0.19 | Y2.V,VII |  |  |
|  | qcGL7-5 | 7H | Ct | 209.43 | 7_28180957 | 3.78 | 1.86 | -0.10 | Y2.V |  |  |
|  | qcGL7-1 | 7H | Rt+Ct | 91.38 | 7HL_29401655 | 3.36 | 27.89 | 0.09 | Y2.ΔT7 |  | cqGL7-2 |
|  | qcGL7-2 | 7H | Rt+Ct | 130.18 | 7HS_35889676 | 4.22-10.15 | 12.77-26.84 | 0.25-0.30 | Y1.IV,VI/Y2.IV |  |  |
|  | qcGL7-3 | 7H | Rt+Ct | 136.75 | 7HS_29196961 | 9.15 | 19.67 | 0.35 | Y2.III | ucqGL7-3 |  |
|  | qcGL7-4 | 7H | Rt+Ct | 141.93 | 7HL_38122468 | 4.02-9.58 | 6.92-8.99 | 0.25-0.26 | Y2.VI,VII | ucqGL7-4 |  |
|  | qcGL7-5 | 7H | Rt+Ct | 149.42 | M_96111_1679 | 11.31 | 33.35 | 0.38 | Y1.III | ucqGL7-5 |  |
|  | qcGL7-6 | 7H | Rt+Ct | 179.43 | 7_135299038 | 7.55 | 5.61 | 0.18 | Y2.V |  |  |
|  | qcGL7-7 | 7H | Rt+Ct | 209.43 | 7_28180957 | 3.96 | 1.72 | -0.10 | Y2.V |  |  |
| GW | qcGW1-1 | 1H | Rt | 18.14 | 1H_37679977 | 5.89 | 4.38 | -0.09 | Y1.V | ucqGW1-1 |  |
|  | qcGW1-2 | 1H | Rt | 23.17 | M_219981_96 | 5.55 | 4.15 | -0.08 | Y2.V |  |  |
|  | qcGW1-3 | 1H | Rt | 49.58 | 1H_65762157 | 4.68-5.30 | 2.79-4.29 | -0.08 to -0.06 | Y2.VI,VII | ucqGW1-2 |  |
|  | qcGW1-4 | 1H | Rt | 78.68 | 1_186628692 | 6.12-6.59 | 3.74-4.12 | -0.08 | Y1.IV,VII |  |  |
|  | qcGW1-5 | 1H | Rt | 140.83 | 1_11525078 | 4.35 | 3.30 | -0.06 | Y2.VI |  |  |
|  | qcGW1-1 | 1H | Ct | 18.14 | 1H_37679977 | 4.76 | 3.46 | -0.08 | Y1.V | ucqGW1-1 |  |
|  | qcGW1-2 | 1H | Ct | 22.53 | 1H_22440310 | 4.15-6.22 | 1.30-4.96 | -0.09 to -0.06 | Y1.IV/Y2.V,VII |  |  |
|  | qcGW1-3 | 1H | Ct | 45.26 | 1_174217948 | 5.85 | 3.09 | -0.07 | Y1.VI |  |  |
|  | qcGW1-4 | 1H | Ct | 78.68 | 1_186628692 | 5.72 | 1.62 | -0.07 | Y1.VII |  |  |
|  | qcGW1-5 | 1H | Ct | 127.55 | 1_12786191 | 5.34 | 3.58 | -0.07 | Y2.VI |  |  |
|  | qcGW1-6 | 1H | Ct | 130.83 | 1_21963834 | 3.30 | 2.31 | -0.05 | Y2.IV |  |  |
|  | qcGW1-7 | 1H | Ct | 141.53 | 1H_11559496 | 3.87 | 0.80 | -0.05 | Y2.V |  |  |
|  | qcGW1-1 | 1H | Rt+Ct | 18.14 | 1H_37679977 | 4.81 | 3.76 | -0.08 | Y1.V |  |  |
|  | qcGW1-2 | 1H | Rt+Ct | 22.74 | 1H_22440310 | 5.39 | 4.17 | -0.08 | Y2.V |  |  |
|  | qcGW1-3 | 1H | Rt+Ct | 45.26 | 1_174217948 | 7.23 | 3.53 | -0.07 | Y1.VI |  |  |
|  | qcGW1-4 | 1H | Rt+Ct | 78.68 | 1_186628692 | 5.62 | 3.32 | -0.07 | Y1.VII |  |  |
|  | qcGW1-5 | 1H | Rt+Ct | 132.59 | 1H_55583778 | 3.28 | 1.10 | -0.40 | Y2.VII |  |  |
|  | qcGW2-1 | 2H | Ct | 52.61 | 2HL_17013042 | 4.26 | 15.51 | -0.06 | Y1.ΔT6 |  | cqGW2-1 |
|  | qcGW2-2 | 2H | Ct | 74.33 | 2HL_47888627 | 3.08 | 5.59 | -0.13 | Y2.ΔT5 |  |  |
|  | qcGW2-3 | 2H | Ct | 83.99 | 2_596664618 | 4.66 | 8.29 | 0.16 | Y2.ΔT5 |  |  |
|  | qcGW2-4 | 2H | Ct | 125.19 | 2HL_22930294 | 4.10-45.17 | 11.06-73.75 | 0.07-0.39 | Y1.II,III,IV,V,VI,VII,ΔT3,ΔT4 | ucqGW2 | cqGW2-4 |
|  |  |  |  |  |  |  |  |  | Y2.III,IV,V,VI,VII |  |  |
|  | qcGW3-1 | 3H | Rt | 10.10 | HvM70 | 3.74 | 1.95 | 0.05 | Y1.VII |  |  |
|  | qcGW3-2 | 3H | Rt | 22.55 | 3HL_38531260 | 3.97 | 3.26 | 0.06 | Y2.VI |  |  |
|  | qcGW3-3 | 3H | Rt | 26.31 | 3_528413521 | 3.45-4.14 | 1.45-14.59 | 0.05-0.06 | Y1.VI,ΔT5 | ucqGW3-1 | cqGW3-1 |
|  | qcGW3-4 | 3H | Rt | 31.47 | 3HL_15958290 | 3.26-5.98 | 3.24-13.26 | -0.09 to -0.07 | Y1.III,IV,ΔT3 | ucqGW3-2 | cqGW3-2 |
|  | qcGW3-5 | 3H | Rt | 157.14 | M_363498_76 | 5.46 | 4.06 | -0.07 | Y2.VI |  |  |
|  | qcGW3-6 | 3H | Rt | 193.44 | 3HS_5478941 | 3.49 | 1.88 | -0.05 | Y1.VII |  |  |
|  | qcGW3-1 | 3H | Ct | 26.31 | 3_528413521 | 3.76 | 13.50 | 0.06 | Y1.ΔT5 | ucqGW3-1 | cqGW3-1 |
|  | qcGW3-2 | 3H | Ct | 31.13 | 3HL_6983780 | 3.95-5.68 | 4.37-18.75 | -0.09 to 0.08 | Y1.III,ΔT3/Y2.VI,ΔT6 | ucqGW3-2 | cqGW3-2 |
|  | qcGW3-3 | 3H | Ct | 36.39 | 3HL_628887 | 3.40 | 1.68 | 0.05 | Y2.VII |  |  |
|  | qcGW3-4 | 3H | Ct | 147.31 | 3HL_17901650 | 4.50 | 2.87 | -0.06 | Y2.VI |  |  |
|  | qcGW3-5 | 3H | Ct | 190.30 | M_168364_1478 | 3.08 | 1.54 | -0.05 | Y1.VI |  |  |
|  | qcGW3-6 | 3H | Ct | 193.44 | 3HS_5478941 | 3.27 | 1.06 | -0.05 | Y1.VII |  |  |
|  | qcGW3-1 | 3H | Rt+Ct | 26.31 | 3_528413521 | 3.31-4.31 | 1.75-15.11 | 0.05-0.06 | Y1.VI,ΔT5 | ucqGW3-1 | cqGW3-1 |
|  | qcGW3-2 | 3H | Rt+Ct | 30.33 | 3_511738805 | 3.53-6.05 | 7.38-23.36 | -0.09 to -0.06 | Y1.III,ΔT3 | ucqGW3-2 | cqGW3-2 |
|  | qcGW3-3 | 3H | Rt+Ct | 157.14 | M_363498_76 | 3.31 | 3.06 | -0.06 | Y2.VI |  |  |
|  | qcGW3-4 | 3H | Rt+Ct | 190.30 | M_168364_1478 | 3.84 | 1.74 | -0.05 | Y1.VI |  |  |
|  | qcGW3-5 | 3H | Rt+Ct | 193.44 | 3HS_5478941 | 4.03 | 2.10 | -0.05 | Y1.VII |  |  |
|  | qcGW5 | 5H | Rt | 3.58 | 5_54803 | 3.43 | 10.81 | -0.05 | Y1.ΔT5 |  | cqGW5 |
|  | qcGW5 | 5H | Ct | 3.58 | 5_54803 | 3.55 | 11.49 | -0.05 | Y1.ΔT5 |  | cqGW5 |
|  | qcGW5 | 5H | Rt+Ct | 4.49 | 5_3871196 | 3.60 | 10.66 | -0.05 | Y1.ΔT5 |  | cqGW5 |
|  | qcGW7-1 | 7H | Rt | 57.85 | 7_524132075 | 9.86 | 7.47 | 0.10 | Y2.V | ucqGW7-1 |  |
|  | qcGW7-2 | 7H | Rt | 66.53 | 7_501748124 | 5.62-9.65 | 5.20-10.34 | 0.07-0.12 | Y1.V,VII/Y2.IV,VII | ucqGW7-2 |  |
|  | qcGW7-3 | 7H | Rt | 72.18 | 7_500618431 | 9.23 | 7.35 | 0.09 | Y2.VI |  |  |
|  | qcGW7-4 | 7H | Rt | 172.81 | 7_180156097 | 5.25 | 3.65 | 0.07 | Y1.IV |  |  |
| GD | qcGD1-1 | 1H | Rt | 18.14 | 1H_37679977 | 4.90 | 2.02 | -0.06 | Y1.V |  |  |
|  | qcGD1-2 | 1H | Rt | 87.43 | 1H_51860442 | 3.37-6.23 | 2.02-4.70 | -0.09 to -0.06 | Y1.IV/Y2.III,IV | ucqGD1-4 |  |
|  | qcGD1-1 | 1H | Ct | 6.49 | 7_344586347 | 3.26 | 0.67 | -0.05 | Y2.VII |  |  |
|  | qcGD1-2 | 1H | Ct | 18.14 | 1H_37679977 | 4.27 | 1.53 | -0.05 | Y1.V |  |  |
|  | qcGD1-3 | 1H | Ct | 20.63 | 1_306385557 | 3.91-3.93 | 2.15-9.60 | -0.10 to -0.05 | Y1.II,IV | ucqGD1-1 |  |
|  | qcGD1-4 | 1H | Ct | 87.19 | 1H_51860442 | 3.82-4.63 | 1.99-4.89 | -0.10 to -0.06 | Y2.III,IV | ucqGD1-4 |  |
|  | qcGD1-5 | 1H | Ct | 132.59 | 1H_55583778 | 4.48 | 0.95 | -0.05 | Y2.VII |  |  |
|  | qcGD1-1 | 1H | Rt+Ct | 18.14 | 1H_37679977 | 3.29 | 1.63 | -0.05 | Y1.V |  |  |
|  | qcGD1-2 | 1H | Rt+Ct | 20.63 | 1_306385557 | 3.97 | 10.00 | -0.10 | Y1.II | ucqGD1-1 |  |
|  | qcGD1-3 | 1H | Rt+Ct | 132.59 | 1H_55583778 | 4.45 | 1.24 | -0.06 | Y2.VII |  |  |
|  | qcGD2-1 | 2H | Ct | 55.78 | 2HL_33276866 | 3.74 | 13.60 | 0.11 | Y1.ΔT3 |  |  |
|  | qcGD2-2 | 2H | Ct | 125.73 | 2_527241334 | 5.48-44.61 | 21.40-71.29 | 0.10-0.38 | Y1.II,III,IV,V,VI,ΔT4 | ucqGD2 |  |
|  |  |  |  |  |  |  |  |  | Y2.III,V,VI,VII,ΔT4 |  |  |
|  | qcGD2-3 | 2H | Ct | 133.61 | M_149956_1482 | 5.10 | 17.45 | 0.15 | Y2.II |  |  |
|  | qcGD3-1 | 3H | Rt | 113.38 | 3HS_26700700 | 5.17 | 2.88 | -0.09 | Y2.VII |  |  |
|  | qcGD3-2 | 3H | Rt | 120.85 | M_123041_952 | 3.34 | 1.66 | -0.06 | Y2.V |  |  |
|  | qcGD3-3 | 3H | Rt | 134.78 | 3HS_15347221 | 3.67 | 1.87 | -0.06 | Y1.VII |  |  |
|  | qcGD3-4 | 3H | Rt | 158.56 | M_1876186_1745 | 3.33 | 2.07 | -0.07 | Y2.VI |  |  |
|  | qcGD3-1 | 3H | Ct | 26.68 | 3_529115904 | 3.49 | 2.81 | 0.07 | Y1.VI | ucqGD3-1 | cqGD3 |
|  | qcGD3-2 | 3H | Ct | 31.13 | 3HL_6983780 | 3.29-7.64 | 2.12-4.86 | -0.08 to 0.08 | Y1.III/Y2.VII | ucqGD3-2 |  |
|  | qcGD3-3 | 3H | Ct | 69.05 | 3_445732233 | 6.07 | 10.12 | 0.14 | Y2.III |  |  |
|  | qcGD3-4 | 3H | Ct | 118.92 | 3_122822375 | 3.71-5.41 | 1.68-9.00 | -0.13 to -0.07 | Y2.III,V,VII |  |  |
|  | qcGD3-5 | 3H | Ct | 147.31 | 3HL_17901650 | 3.59 | 1.36 | -0.07 | Y2.VII |  |  |
|  | qcGD3-1 | 3H | Rt+Ct | 21.97 | 7HS_22292244 | 5.35 | 1.65 | 0.07 | Y2.VII |  |  |
|  | qcGD3-2 | 3H | Rt+Ct | 118.92 | 3_122822375 | 3.84 | 1.97 | -0.06 | Y2.V |  |  |
|  | qcGD3-3 | 3H | Rt+Ct | 154.73 | 3HL_22346532 | 6.09 | 1.79 | -0.07 | Y2.VII |  |  |
|  | qcGD4-1 | 4H | Rt | 59.54 | 4HL_5092076 | 3.24 | 2.05 | -0.07 | Y2.VI |  |  |
|  | qcGD4-2 | 4H | Rt | 71.23 | 2_508501818 | 3.92 | 12.98 | -0.05 | Y2.ΔT6 |  |  |
|  | qcGD4-3 | 4H | Rt | 139.62 | 4_42378533 | 4.70 | 2.89 | -0.08 | Y1.VII |  |  |
|  | qcGD4-4 | 4H | Rt | 161.19 | M_1611184_441 | 3.58 | 2.11 | 0.07 | Y1.VII |  |  |
|  | qcGD4-1 | 4H | Ct | 57.07 | 4HL_38170681 | 3.08 | 1.68 | -0.06 | Y1.V |  |  |
|  | qcGD4-2 | 4H | Ct | 72.19 | Bmac30 | 5.28 | 1.17 | -0.06 | Y2.VII |  |  |
|  | qcGD4-1 | 4H | Rt+Ct | 71.57 | 4HL_20849278 | 3.11-3.34 | 1.08-11.72 | -0.05 to 0.06 | Y2.VII,ΔT6 |  |  |
|  | qcGD4-2 | 4H | Rt+Ct | 161.19 | M_1611184_441 | 3.62 | 2.32 | 0.06 | Y1.VI |  |  |
|  | qcGD5 | 5H | Ct | 139.72 | 1H_81979810 | 3.64 | 2.12 | -0.06 | Y1.VI |  |  |
|  | qcGD6 | 6H | Rt+Ct | 104.69 | 6HL_13081351 | 3.12 | 1.22 | 0.05 | Y2.V |  |  |
|  | qcGD7-1 | 7H | Rt | 65.75 | 7HL_37199773 | 4.12-12.79 | 9.80-11.33 | 0.12-0.15 | Y1.V,VI/Y2.IV,V | ucqGD7-1 |  |
|  | qcGD7-2 | 7H | Rt | 71.08 | 7HL_18681541 | 11.00-19.79 | 14.11-34.37 | 0.09-0.18 | Y1.VII/Y2.ΔT7 |  | cqGD7-2 |
|  | qcGD7-3 | 7H | Rt | 76.14 | 7_500661278 | 3.18-14.75 | 11.11-28.84 | 0.09-0.20 | Y2.III,ΔT3 | ucqGD7-2 |  |
|  | qcGD7-4 | 7H | Rt | 79.71 | GMS46 | 6.47 | 17.70 | 0.06 | Y1.ΔT7 |  |  |
|  | qcGD7-5 | 7H | Rt | 95.89 | 4HL_39191572 | 7.04 | 10.85 | 0.12 | Y1.III | ucqGD7-3 |  |
|  | qcGD7-6 | 7H | Rt | 118.25 | 7_262149691 | 3.03-3.35 | 7.24-9.77 | 0.12-0.13 | Y1.IV/Y2.IV |  |  |
|  | qcGD7-7 | 7H | Rt | 151.09 | Bmac31 | 24.16-31.02 | 25.88-34.85 | 0.24-0.30 | Y2.VI,VII | ucqGD7-4 |  |
|  | qcGD7-8 | 7H | Rt | 165.67 | 7HS_5799924 | 4.56 | 5.63 | 0.11 | Y2.V | ucqGD7-5 |  |
|  | qcGD7-9 | 7H | Rt | 198.99 | Bmag914 | 3.62 | 2.09 | 0.06 | Y1.V |  |  |
|  | qcGD7-10 | 7H | Rt | 208.78 | 7_31360519 | 3.05 | 9.59 | 0.04 | Y2.ΔT6 |  |  |
|  | qcGD7-11 | 7H | Rt | 225.53 | 7HS_22537347 | 3.49 | 1.72 | -0.06 | Y1.V |  |  |
|  | qcGD7-1 | 7H | Ct | 118.25 | 7_262149691 | 4.10-6.56 | 3.43-7.22 | 0.10-0.13 | Y2.VI,VII |  |  |
|  | qcGD7-2 | 7H | Ct | 180.01 | 7HS_16458224 | 3.54-5.02 | 4.43-7.89 | 0.09-0.13 | Y1.III,IV,VII |  |  |
|  | qcGD7-3 | 7H | Ct | 198.99 | Bmag914 | 3.60 | 2.67 | 0.07 | Y1.V |  |  |
|  | qcGD7-4 | 7H | Ct | 225.53 | 7HS_22537347 | 4.02 | 2.19 | -0.06 | Y1.V |  |  |
|  | qcGD7-1 | 7H | Rt+Ct | 118.25 | 7_262149691 | 4.47-5.52 | 5.15-8.62 | 0.12-0.13 | Y2.IV,VI,VII |  |  |
|  | qcGD7-2 | 7H | Rt+Ct | 165.67 | 7HS_5799924 | 5.04 | 6.45 | 0.11 | Y2.V | ucqGD7-5 |  |
|  | qcGD7-3 | 7H | Rt+Ct | 198.99 | Bmag914 | 4.04 | 2.32 | 0.07 | Y1.V |  |  |
|  | qcGD7-4 | 7H | Rt+Ct | 225.53 | 7HS_22537347 | 3.67 | 2.11 | -0.06 | Y1.V |  |  |
| GFR | qcGFR1-1 | 1H | Rt | 17.50 | 1_304812961 | 5.87 | 3.43 | -0.10 | Y2.V |  |  |
|  | qcGFR1-2 | 1H | Rt | 78.40 | 1H_61373505 | 3.42-3.79 | 2.50-4.18 | -0.09 to -0.05 | Y1.IV,ΔT3/Y2.ΔT3 |  |  |
|  | qcGFR1-1 | 1H | Ct | 20.63 | 1_306385557 | 4.86 | 2.47 | -0.08 | Y1.III |  | cqGFR1-1 |
|  | qcGFR1-2 | 1H | Ct | 48.71 | 1H_81216340 | 6.20 | 1.82 | -0.09 | Y2.IV |  |  |
|  | qcGFR1-3 | 1H | Ct | 115.68 | 1_58149966 | 3.53 | 1.85 | -0.07 | Y2.III |  |  |
|  | qcGFR1-1 | 1H | Rt+Ct | 17.50 | 1_304812961 | 5.03 | 3.17 | -0.10 | Y2.IV |  |  |
|  | qcGFR1-2 | 1H | Rt+Ct | 50.36 | 1_113838203 | 3.19-3.37 | 3.08-3.78 | -0.07 | Y1.III/Y2.III |  |  |
|  | qcGFR2-1 | 2H | Ct | 46.93 | Bmac93 | 3.02-11.75 | 3.24-16.07 | -0.17 to 0.27 | Y1.IV/Y2.IV,ΔT6 |  |  |
|  | qcGFR2-2 | 2H | Ct | 88.11 | Bmag829 | 3.12-4.19 | 1.42-9.39 | -0.12 to 0.09 | Y1.III,ΔT3,ΔT5/Y2.III,ΔT5 | ucqGFR2-2 | cqGFR2-1 |
|  | qcGFR2-3 | 2H | Ct | 125.73 | 2_527241334 | 4.19-39.57 | 25.13-70.94 | -0.28 to 0.49 | Y1.III,IV,V,ΔT2,ΔT3,ΔT4,ΔT5,ΔT6 | ucqGFR2-3 | cqGFR2-2 |
|  |  |  |  |  |  |  |  |  | Y2.III,IV,V,ΔT2,ΔT3,ΔT4,ΔT5,ΔT6 |  |  |
|  | qcGFR2 | 2H | Rt+Ct | 46.81 | Bmac93 | 3.52 | 2.35 | 0.08 | Y2.IV |  |  |
|  | qcGFR3-1 | 3H | Rt | 32.63 | 3_504148850 | 3.06-10.61 | 3.97-26.65 | -0.09 to 0.18 | Y1.II,III,VI,ΔT3,ΔT5 | ucqGFR3-2 | cqGFR3-2 |
|  |  |  |  |  |  |  |  |  | Y2.III,V,VI,VII,ΔT3,ΔT4,ΔT5,ΔT7 |  |  |
|  | qcGFR3-2 | 3H | Rt | 37.89 | 5HL_48131831 | 4.71-5.86 | 11.37-12.80 | -0.05 to 0.14 | Y1.V,ΔT7 |  |  |
|  | qcGFR3-3 | 3H | Rt | 69.68 | 7HS_39715161 | 5.34 | 9.24 | -0.13 | Y1.V |  |  |
|  | qcGFR3-1 | 3H | Ct | 32.72 | 3_511749149 | 3.71-10.18 | 2.84-28.61 | -0.09 to 0.16 | Y1.III,VI,ΔT3,ΔT5 | ucqGFR3-2 | cqGFR3-2 |
|  |  |  |  |  |  |  |  |  | Y2.III,V,VI,VII,ΔT4,ΔT5 |  |  |
|  | qcGFR3-2 | 3H | Ct | 37.89 | 5HL_48131831 | 5.67-6.29 | 6.03-9.78 | 0.10-0.13 | Y1.V,ΔT4 |  |  |
|  | qcGFR3-3 | 3H | Ct | 155.32 | 3_230154353 | 3.95 | 2.94 | -0.09 | Y1.V |  |  |
|  | qcGFR3-1 | 3H | Rt+Ct | 32.63 | 3_504148850 | 3.65-9.77 | 3.78-28.50 | -0.09 to 0.16 | Y1.III,VI,ΔT4,ΔT5 | ucqGFR3-2 | cqGFR3-2 |
|  |  |  |  |  |  |  |  |  | Y2.III,V,VI,VII,ΔT4,ΔT5,ΔT7 |  |  |
|  | qcGFR3-2 | 3H | Rt+Ct | 37.89 | 5HL_48131831 | 6.10 | 9.31 | 0.13 | Y1.V |  |  |
|  | qcGFR4-1 | 4H | Rt | 57.23 | 4_261703040 | 3.19 | 3.25 | -0.07 | Y1.III |  |  |
|  | qcGFR4-2 | 4H | Rt | 143.67 | 4_28741355 | 3.88-4.82 | 2.14-4.20 | 0.04-0.08 | Y1.III,ΔT3 |  |  |
|  | qcGFR4-1 | 4H | Rt+Ct | 58.02 | 4HL_23617323 | 3.25-3.85 | 1.52-3.95 | -0.07 to -0.05 | Y1.III/Y2.III,ΔT3 |  |  |
|  | qcGFR4-2 | 4H | Rt+Ct | 143.67 | 4_28741355 | 3.04-4.21 | 3.39-6.32 | 0.07 | Y2.III,ΔT3 |  |  |
|  | qcGFR5-1 | 5H | Rt | 0.00 | 5HS_7374618 | 3.23 | 4.31 | -0.08 | Y2.ΔT5 | ucqGFR5 | cqGFR5 |
|  | qcGFR5-2 | 5H | Rt | 207.85 | 5HL_25802367 | 4.27 | 3.30 | 0.05 | Y1.ΔT3 |  |  |
|  | qcGFR5-1 | 5H | Ct | 0.00 | 5HS_7374618 | 3.31-5.33 | 4.28-7.51 | -0.11 to -0.08 | Y1.V,ΔT4/Y2.V | ucqGFR5 | cqGFR5 |
|  | qcGFR5-2 | 5H | Ct | 6.69 | 5_3910746 | 3.16 | 3.29 | -0.06 | Y2.ΔT4 |  |  |
|  | qcGFR5-1 | 5H | Rt+Ct | 0.00 | 5HS_7374618 | 3.80-5.23 | 4.95-7.01 | -0.11 to -0.08 | Y1.V,ΔT4/Y2.V | ucqGFR5 | cqGFR5 |
|  | qcGFR5-2 | 5H | Rt+Ct | 6.69 | 5_3910746 | 3.30 | 5.28 | -0.06 | Y2.ΔT4 |  |  |
|  | qcGFR7-1 | 7H | Rt | 58.01 | 1_4089724 | 4.92-8.18 | 6.75-12.08 | 0.09-0.13 | Y1.V/Y2.V,ΔT4 |  | cqGFR7-1 |
|  | qcGFR7-2 | 7H | Rt | 65.18 | 7HL_13143105 | 6.79 | 9.07 | -0.10 | Y2.ΔT6 | ucqGFR7-1 | cqGFR7-2 |
|  | qcGFR7-3 | 7H | Rt | 85.89 | M_69439_721 | 3.34-7.84 | 5.84-9.08 | -0.08 to 0.13 | Y2.IV,ΔT6 |  |  |
|  | qcGFR7-4 | 7H | Rt | 141.93 | 7HL_38122468 | 6.41-6.74 | 7.83-7.92 | 0.08-0.10 | Y2.III,ΔT3 |  |  |
|  | qcGFR7-5 | 7H | Rt | 156.50 | 2HS_16886086 | 9.47 | 8.14 | 0.17 | Y1.IV |  |  |
|  | qcGFR7-6 | 7H | Rt | 172.81 | 7_180156097 | 6.16 | 5.77 | 0.09 | Y1.III |  |  |
|  | qcGFR7-7 | 7H | Rt | 180.69 | 7HS_39727659 | 5.97 | 6.50 | 0.07 | Y1.ΔT3 | ucqGFR7-2 |  |
|  | qcGFR7-8 | 7H | Rt | 183.45 | 7HS_33683527 | 4.70 | 3.28 | 0.10 | Y2.IV |  |  |
|  | qcGFR7-1 | 7H | Ct | 172.73 | 7_152087825 | 4.03 | 3.38 | 0.10 | Y1.III |  |  |
|  | qcGFR7-2 | 7H | Ct | 183.45 | 7HS_33683527 | 3.04-4.76 | 3.14-4.12 | -0.07 to 0.12 | Y1.ΔT6/Y2.Ⅳ |  |  |
|  | qcGFR7-1 | 7H | Rt+Ct | 172.81 | 7_180156097 | 3.42 | 5.51 | 0.09 | Y1.III |  |  |
|  | qcGFR7-2 | 7H | Rt+Ct | 183.45 | 7HS_33683527 | 4.43 | 3.52 | 0.10 | Y2.Ⅳ |  |  |
| GFR_max_ | qcGFR_max_1 | 1H | Rt | 17.50 | 1_304812961 | 5.67 | 3.22 | -0.11 | Y2 |  |  |
|  | qcGFR_max_1 | 1H | Ct | 48.71 | 1H_81216340 | 6.21 | 4.02 | -0.12 | Y2 |  |  |
|  | qcGFR_max_1 | 1H | Rt+Ct | 78.40 | 1H_61373505 | 5.22 | 2.77 | -0.10 | Y2 |  |  |
|  | qcGFR_max_2 | 2H | Rt | 69.66 | 2HL_18957514 | 3.11 | 2.24 | 0.09 | Y2 |  |  |
|  | qcGFR_max_2-1 | 2H | Ct | 46.81 | Bmac93 | 4.04 | 3.83 | 0.12 | Y1 |  |  |
|  | qcGFR_max_2-2 | 2H | Ct | 69.66 | 2HL_18957514 | 4.86 | 3.89 | 0.12 | Y2 |  |  |
|  | qcGFR_max_2-3 | 2H | Ct | 126.97 | 2HL_22930005 | 33.18-37.16 | 68.04-69.46 | 0.49-0.52 | Y1/Y2 | ucqGFRmax2-2 |  |
|  | qcGFR_max_2-1 | 2H | Rt+Ct | 69.66 | 2HL_18957514 | 4.69 | 3.26 | 0.11 | Y2 |  |  |
|  | qcGFR_max_6 | 6H | Rt+Ct | 68.26 | 6HL_24721940 | 3.07 | 1.58 | 0.07 | Y2 |  |  |
|  | qcGFR_max_7-1 | 7H | Rt | 85.89 | M_69439_721 | 5.36 | 5.20 | 0.13 | Y2 |  |  |
|  | qcGFR_max_7-2 | 7H | Rt | 183.45 | 7HS_33683527 | 3.45 | 3.19 | 0.11 | Y2 |  |  |
|  | qcGFR_max_7 | 7H | Ct | 183.45 | 7HS_33683527 | 4.65 | 3.66 | 0.11 | Y2 |  |  |
|  | qcGFR_max_7-1 | 7H | Rt+Ct | 183.45 | 7HS_33683527 | 5.03 | 3.40 | 0.11 | Y2 |  |  |
| GFR_mean_ | qcGFR_mean_2 | 2H | Ct | 125.19 | 2HL_22930294 | 35.14-45.74 | 66.66-72.18 | 0.16-0.17 | Y1/Y2 | ucqGFRmean2 |  |
|  | qcGFR_mean_3 | 3H | Ct | 78.69 | 3HL_21306461 | 4.09 | 2.97 | -0.03 | Y1 |  |  |
|  | qcGFR_mean_7 | 7H | Rt | 181.34 | 7HS_23607469 | 7.50 | 6.89 | 0.05 | Y1 |  |  |
|  | qcGFR_mean_7 | 7H | Rt+Ct | 157.12 | M_231959_191 | 3.39 | 3.51 | 0.04 | Y1 |  |  |

**Table S5. Detailed information on the unique QTLs that integrated from the** **unconditional and conditional consensus QTLs for GFR and five grain size traits.**

**^a^** U, unconditional QTL; C, conditional QTL; B, including both unconditional QTL and conditional QTL;

| Trait | Unique QTLs | | | | |  | Consensus QTLs | | | | | | | |
| --- | --- | --- | --- | --- | --- | --- | --- | --- | --- | --- | --- | --- | --- | --- |
|  | QTLs | Position | The closest marker | CI | Type ^a^ |  | QTLs | Chr. | Position | LOD | R^2^ | Add | CI | Stage |
| GFR | uqGFR1-1 | 22 | M_1601731_2584 | 21.5-22.5 | C |  | cqGFR1-1 | 1H | 22 | 3.22 | 3.99 | -0.02 | 21.5-22.5 | Y1.ΔT2 |
|  | uqGFR1-2 | 37.79 | 1H_82851228 | 37.35 - 38.24 | U |  | ucqGFR1-1 | 1H | 37.79 | 33.24-34.12 | 16.33-19.47 | 0.10-0.11 | 37.35-38.24 | Y1.II/Y2.II |
|  | uqGFR1-3 | 42 | 1H_10863328 | 41.71 - 42.28 | U |  | ucqGFR1-2 | 1H | 42 | 7.03-47.17 | 7.12-36.22 | -0.18 to -0.12 | 41.71-42.28 | Y2.II,III |
|  | uqGFR1-4 | 54 | 1H_58188215 | 52.5-54.5 | B |  | cqGFR1-2 | 1H | 54 | 5.23 | 5.80 | -0.06 | 52.5-54.5 | Y2.ΔT3 |
|  |  |  |  |  |  |  | ucqGFR1-3 | 1H | 54 | 6.81 | 4.4 | -0.11 | 52.4-54.5 | Y2.IV |
|  | uqGFR2-1 | 68.5 | M_1999039_479 | 68.28-68.87 | U |  | ucqGFR2-1 | 2H | 68.5 | 3.10-4.12 | 2.69-2.83 | -0.11 to 0.09 | 68.28-68.87 | Y1.IV/Y2.III |
|  | uqGFR2-2 | 88 | Bmag829 | 87.35-88.24 | B |  | cqGFR2-1 | 2H | 88 | 3.74-4.69 | 1.26-12.78 | -0.12 to 0.05 | 87.35-88.24 | Y1.ΔT3,ΔT5, Y2.ΔT3,ΔT5 |
|  |  |  |  |  |  |  | ucqGFR2-2 | 2H | 88 | 4.85 | 12.2 | -0.05 | 87.5-89.5 | Y2.VII |
|  | uqGFR2-3 | 126.5 | 2HL_22930005 | 126.25-126.75 | B |  | ucqGFR2-3 | 2H | 126.25 | 15.57-46.56 | 33.71-71.54 | 0.08-0.51 | 125.75-127.5 | Y1.II,III,IV,V/Y2.III,IV,V |
|  |  |  |  |  |  |  | cqGFR2-2 | 2H | 126.83 | 6.51-39.08 | 14.92-65.72 | -0.28 to 0.23 | 126.45-127.25 | Y1.ΔT2,ΔT3,ΔT4,ΔT6,ΔT7  Y2.ΔT2,ΔT3, ΔT4,ΔT5,ΔT6,ΔT7 |
|  |  |  |  |  |  |  |  |  |  |  |  |  |  |  |
|  | uqGFR2-4 | 132 | 2HL_43143355 | 131.5-132.5 | C |  | cqGFR2-3 | 2H | 132 | 8.69 | 26.63 | -0.19 | 131.5-132.5 | Y1.ΔT5 |
|  | uqGFR3-1 | 28 | 3_525094736 | 27.64-28.35 | B |  | ucqGFR3-1 | 3H | 28 | 6.04-8.81 | 9.66-14.26 | 0.13-0.14 | 27.64-28.35 | Y1.V/Y2.V |
|  |  |  |  |  |  |  | cqGFR3-1 | 3H | 28 | 4.88-6.94 | 12.25-14.46 | -0.06 to 0.10 | 27.64-28.35 | Y1.ΔT7/Y2.ΔT4 |
|  | uqGFR3-2 | 32.64 | 3_504148850 | 32.33-33.14 | B |  | ucqGFR3-2 | 3H | 32 | 4.39-7.32 | 4.76-24.12 | -0.10 to 0.11 | 31.71-32.28 | Y1.II.III.VI, Y2.III.VI.VII |
|  |  |  |  |  |  |  | cqGFR3-2 | 3H | 33 | 4.06-9.63 | 1.40-22.86 | -0.07 to 0.16 | 32.66-33.33 | Y1.ΔT3,ΔT5/Y2. ΔT2,ΔT3,ΔT5,ΔT7 |
|  | uqGFR5-1 | 0 | 5HS_7374618 | 0-0.5 | B |  | ucqGFR5 | 5H | 0 | 5.15 | 7.71 | -0.10 | 0-0.5 | Y1.V/Y2.V |
|  |  |  |  |  |  |  | cqGFR5 | 5H | 0.03 | 3.19-3.68 | 5.17-5.35 | -0.07 to -0.06 | 0.21-0.28 | Y1.ΔT4/Y2.ΔT4 |
|  | uqGFR7-1 | 58 | 1_4089724 | 57.5-58.5 | C |  | cqGFR7-1 | 7H | 58 | 5.06-5.52 | 7.49-9.44 | 0.08-0.09 | 57.5-58.5 | Y1.ΔT4/Y2.ΔT4 |
|  | uqGFR7-2 | 66.25 | 7HL_4313756 | 65.55-66.25 | B |  | ucqGFR7-1 | 7H | 66.5 | 5.35-12.69 | 7.66-9.96 | 0.10-0.17 | 66.25-66.75 | Y1.IV.V, Y2.V |
|  |  |  |  |  |  |  | cqGFR7-2 | 7H | 65.5 | 3.77-5.75 | 8.46-11.91 | -0.09 to -0.04 | 65.25-65.75 | Y1.ΔT6,ΔT7/Y2.ΔT7 |
|  | uqGFR7-3 | 122 | 7HS_21726812 | 120.5-122.5 | C |  | cqGFR7-3 | 7H | 122 | 6.25 | 8.52 | -0.08 | 120.5-122.5 | Y1.ΔT6 |
|  | uqGFR7-4 | 133 | 7HS_17906516 | 132.5-134.5 | C |  | cqGFR7-4 | 7H | 133 | 8.66 | 16.27 | -0.11 | 132.5-134.5 | Y1.ΔT7 |
|  | uqGFR7-5 | 180 | 7HS_16458224 | 179.5-180.5 | U |  | ucqGFR7-2 | 7H | 180 | 4.74-8.18 | 7.63-8.07 | 0.03-0.10 | 179.5-180.5 | Y1.II.III |
| GA | uqGA1-1 | 22 | 1_306394013 | -0.41 to -0.38 | U |  | ucqGA1-1 | 1H | 22 | 3.23-3.57 | 7.65-8.64 | -0.41 to -0.38 | 21.64-22.35 | Y1.II/Y2.II |
|  | uqGA1-2 | 64 | M_2579923_225 | 0.81 | U |  | ucqGA1-2 | 1H | 64 | 16.69 | 7.44 | 0.81 | 62.5-64.5 | Y1.IV |
|  | uqGA1-3 | 87 | 1H_2112459 | -1.15 | U |  | ucqGA1-3 | 1H | 87 | 26.95 | 15.06 | -1.15 | 86.5-87.5 | Y1.IV |
|  | uqGA1-4 | 117 | 1_41186142 | -0.48 | U |  | ucqGA1-4 | 1H | 117 | 5.49 | 3.32 | -0.48 | 116.5-117.5 | Y2.IV |
|  | uqGA1-5 | 133 | 1_19825452 | -0.50 to -0.39 | U |  | ucqGA1-5 | 1H | 133 | 3.92-4.60 | 1.29-4.31 | -0.50 to -0.39 | 130.5-133.5 | Y1.VI,VII |
|  | uqGA2-1 | 121 | 2_506545106 | 120.5-122.5 | C |  | cqGA2-1 | 2H | 121 | 4.00 | 8.35 | 0.30 | 120.5-122.5 | Y1.ΔT5 |
|  | uqGA2-2 | 127.34 | 1_12786191 | 126.65-127.74 | B |  | ucqGA2 | 2H | 126.13 | 9.51-55.19 | 25.91-75.68 | 0.76-2.85 | 125.55-126.64 | Y1.II,III,IV,V,VI,VII/Y2.II,III,IV,V,VII |
|  |  |  |  |  |  |  | cqGA2-2 | 2H | 127.54 | 9.25-15.63 | 27.71-40.97 | 0.77-0.88 | 127.13-127.84 | Y1.ΔT4/Y2.ΔT3,ΔT4 |
|  | uqGA3-1 | 30 | 3_510997641 | 29.5-30.5 | U |  | ucqGA3-1 | 3H | 30 | 4.39 | 1.544 | 0.4592 | 29.5-30.5 | Y2.VII |
|  | uqGA3-2 | 33.3 | 3_511749149 | 32.47-34.13 | C |  | cqGA3-1 | 3H | 33.3 | 3.12-4.22 | 6.79-9.36 | 0.23-0.35 | 32.47-34.13 | Y1.ΔT6/Y2.ΔT6 |
|  | uqGA3-3 | 41 | 3_499436820 | 39.93-42.06 | C |  | cqGA3-2 | 3H | 41 | 5.06-5.14 | 10.48-12.13 | 0.32-0.38 | 39.93-42.06 | Y1.ΔT5/Y2.ΔT5 |
|  | uqGA3-4 | 57 | 3HL_34537138 | 56.5-57.5 | U |  | ucqGA3-2 | 3H | 57 | 5.13 | 1.7014 | 0.45 | 56.5-57.5 | Y2.VII |
|  | uqGA3-5 | 91 | 3_267212934 | 90.5-91.5 | U |  | ucqGA3-3 | 3H | 91 | 5.03 | 4.8477 | -0.53 | 90.5-91.5 | Y2.VI |
|  | uqGA4-1 | 10 | 4HL_29463683 | 9.5-11.5 | U |  | ucqGA4 | 4H | 10 | 3.931 | 1.7429 | -0.37 | 9.5-11.5 | Y1.V |
|  | uqGA4-2 | 90 | 4HL_42790942 | 89.5-90.5 | C |  | cqGA4 | 4H | 90 | 3.48 | 7.42 | -0.23 | 89.5-90.5 | Y2.ΔT6 |
|  | uqGA5-1 | 11 | 5HS_10560611 | 9.5-11.5 | C |  | cqGA5-1 | 5H | 11 | 4.79 | 9.98 | -0.30 | 9.5-11.5 | Y1.ΔT5 |
|  | uqGA5-2 | 141 | M_1634918_588 | 140.5-141.5 | U |  | ucqGA5 | 5H | 141 | 3.19 | 1.43 | -0.35 | 140.5-141.5 | Y1.V |
|  | uqGA5-3 | 203 | 5_226253827 | 202.5-203.5 | C |  | cqGA5-2 | 5H | 203 | 3.74 | 8.59 | -0.31 | 202.5-203.5 | Y2.ΔT5 |
|  | uqGA7-1 | 58 | 1_4089724 | 57.5-58.5 | C |  | cqGA7-1 | 7H | 58 | 4.78 | 11.32 | 0.46 | 57.5-58.5 | Y2.ΔT4 |
|  | uqGA7-2 | 65 | 7HL_8312277 | 64.75-65.24 | B |  | cqGA7-2 | 7H | 65 | 7.85 | 27.58 | 0.22 | 64.5-65.5 | Y2.ΔT7 |
|  |  |  |  |  |  |  | ucqGA7-1 | 7H | 65 | 12.66-35.07 | 9.43-30.11 | 0.8-1.6 | 64.71-65.28 | Y2.IV,VI,VII |
|  | uqGA7-3 | 94 | Bmag746 | 93.64 - 94.35 | U |  | ucqGA7-2 | 7H | 94 | 13.42-13.47 | 10.99-11.34 | 1.07-1.10 | 93.64-94.35 | Y1.VI,VII |
|  | uqGA7-4 | 97 | 7_440111505 | 96.5-97.5 | C |  | cqGA7-3 | 7H | 97 | 7.54 | 18.97 | 0.46 | 96.5-97.5 | Y2.ΔT5 |
|  | uqGA7-5 | 151 | Bmac31 | 150.5 - 151.5 | U |  | ucqGA7-3 | 7H | 151 | 15.61 | 6.44 | 0.87 | 150.5-151.5 | Y2.VII |
|  | uqGA7-6 | 165 | 7_194302352 | 164.29-165.7 | C |  | cqGA7-4 | 7H | 165 | 3.22-4.48 | 11.45-12.31 | 0.42-0.52 | 164.29-165.7 | Y1.ΔT3/Y2.ΔT3 |
| GP | uqGP1-1 | 22 | 1_306394013 | 21.5-22.5 | U |  | ucqGP1-1 | 1H | 22 | 4.04 | 10.26 | -0.54 | 21.5-22.5 | Y1.II |
|  | uqGP1-2 | 42 | 1H_10863328 | 41.5-42.5 | U |  | ucqGP1-2 | 1H | 42 | 3.62 | 7.82 | -0.51 | 41.5-42.5 | Y2.II |
|  | uqGP2-1 | 126.25 | 2_527241334 | 125.93-126.54 | U |  | ucqGP2-1 | 2H | 126.25 | 8.21-37.66 | 15.96-62.17 | 0.52-1.21 | 125.93-126.54 | Y1.II,III,IV,V,VI,VII/Y2.II,III,IV,VI,VII |
|  | uqGP2-2 | 132 | 2HL_43143355 | 131.5 - 132.5 | U |  | ucqGP2-2 | 2H | 132 | 25.32 | 24.71 | 0.90 | 131.5-132.5 | Y2.V |
|  | uqGP3-1 | 55 | 3HL_48064911 | 54.13 - 55.86 | U |  | ucqGP3-1 | 3H | 55 | 3.39-9.21 | 3.25-10.44 | 0.24-0.39 | 54.13-55.86 | Y1.V,VI/Y2.IV |
|  | uqGP3-2 | 91 | 3_267212934 | 90.64 - 91.35 | U |  | ucqGP3-2 | 3H | 91 | 6.36-11.91 | 5.14-13.83 | -0.45 to -0.30 | 90.64-91.35 | Y1.V,VI |
|  | uqGP5-1 | 203 | 5_226253827 | 202.5-203.5 | U |  | ucqGP5 | 5H | 203 | 4.76 | 4.78 | -0.26 | 202.5-203.5 | Y1.VI |
|  | uqGP6-1 | 102 | M_1661027_233 | 101.5-102.5 | U |  | ucqGP6 | 6H | 102 | 30.3724 | 31.4551 | 0.938 | 101.5-102.5 | Y2.V |
|  | uqGP7-1 | 49 | GBM1102 | 47.5-50.5 | U |  | ucqGP7-1 | 7H | 49 | 5.26 | 5.45 | 0.31 | 47.5-50.5 | Y2.III |
|  | uqGP7-2 | 57 | 1_4088556 | 56.5-57.5 | C |  | cqGP7-1 | 7H | 57 | 3.50 | 14.30 | 0.52 | 56.5-57.5 | Y2.ΔT3 |
|  | uqGP7-3 | 65.5 | 7HL_37199773 | 65.25 - 65.75 | U |  | ucqGP7-2 | 7H | 65.5 | 11.84-31.98 | 13.73-44.71 | 0.49-1.23 | 65.25-65.75 | Y2.IV,V,VI,VII |
|  | uqGP7-4 | 68.99 | 7HL_3360534 | 68.64-69.35 | C |  | cqGP7-2 | 7H | 68.99 | 5.01-13.78 | 17.05-41.39 | 0.20-0.52 | 68.64-69.35 | Y2.ΔT3,ΔT6 |
|  | uqGP7-5 | 94 | Bmag746 | 93.64 - 94.35 | U |  | ucqGP7-3 | 7H | 94 | 9.36-14.66 | 9.99-13.89 | 0.42-0.69 | 93.64-94.35 | Y2.III,VII |
|  | uqGP7-6 | 116 | 5_496886371 | 115.5-116.5 | U |  | ucqGP7-4 | 7H | 116 | 17.12 | 21.04 | 0.61 | 115.5-116.5 | Y2.III |
|  | uqGP7-7 | 124 | 7HS_32890650 | 123.5-124.5 | C |  | cqGP7-3 | 7H | 124 | 25.21 | 16.88 | 1.57 | 123.5-124.5 | Y1.ΔT3 |
|  | uqGP7-8 | 136 | 7HS_29196961 | 135.5-136.5 | U |  | ucqGP7-5 | 7H | 136 | 27.36 | 26.39 | -1.04 | 135.5-136.5 | Y2.VI |
|  | uqGP7-9 | 140 | 7_319506952 | 138.5-140.5 | U |  | ucqGP7-6 | 7H | 140 | 21.31 | 30.24 | 0.77 | 138.5-140.5 | Y1.III |
|  | uqGP7-10 | 152.15 | 7HL_29535430 | 151.87-152.42 | B |  | ucqGP7-7 | 7H | 151.77 | 7.18-22.28 | 16.00-30.90 | 0.52-0.75 | 151.44-152.11 | Y1.VI/Y2.II,IV |
|  |  |  |  |  |  |  | cqGP7-4 | 7H | 153 | 18.28 | 10.41 | -1.22 | 152.5-153.5 | Y1.ΔT3 |
| GL | uqGL1-1 | 19 | 1H_17392124 | 18.64-19.35 | C |  | cqGL1-1 | 1H | 19 | 3.02-8.28 | 11.34-14.00 | 0.21-0.32 | 18.64-19.35 | Y1.ΔT3/Y2.ΔT3 |
|  | uqGL1-2 | 22 | 1_306394013 | 21.5-22.5 | U |  | ucqGL1 | 1H | 22 | 5.33 | 12.70 | -0.29 | 21.5-22.5 | Y1.II |
|  | uqGL1-3 | 114 | 1H_34522869 | 113.5-115.5 | C |  | cqGL1-2 | 1H | 114 | 5.68 | 9.06 | -0.26 | 113.5-115.5 | Y2.ΔT3 |
|  | uqGL2-1 | 125.94 | 2_527241334 | 125.76 - 126.11 | U |  | ucqGL2-1 | 2H | 125.94 | 12.44-29.66 | 15.52-51.03 | 0.35-0.45 | 125.76-126.11 | Y1.III,IV,V,VI,VII/Y2.III,IV,V,VI,VII |
|  | uqGL2-2 | 133.1 | 2_534686550 | 132.62 - 133.57 | U |  | ucqGL2-2 | 2H | 133.1 | 5.39-5.79 | 12.84-14.43 | 0.31-0.34 | 132.62-133.57 | Y1.II/Y2.II |
|  | uqGL3-1 | 19 | 3HL_42780152 | 17.5-19.5 | C |  | cqGL3 | 3H | 19 | 4.61 | 7.29 | 0.24 | 17.5-19.5 | Y2.ΔT3 |
|  | uqGL3-2 | 48 | 3HL_33828484 | 47.18 - 47.87 | U |  | ucqGL3-1 | 3H | 48 | 4.35-5.53 | 3.96-7.23 | 0.12-0.13 | 47.18-47.87 | Y1.V/Y2.IV |
|  | uqGL3-3 | 91 | 3_267212934 | 90.5-91.5 | U |  | ucqGL3-2 | 3H | 91 | 6.59 | 6.36 | -0.15 | 90.5-91.5 | Y1.V |
|  | uqGL5-1 | 157 | 5_306133226 | 156.5-157.5 | U |  | ucqGL5 | 5H | 157 | 3.25 | 2.94 | -0.11 | 156.5-157.5 | Y1.V |
|  | uqGL7-1 | 65.57 | 7HL_37199773 | 65.38-65.76 | B |  | ucqGL7-1 | 7H | 65.25 | 15.66-50.97 | 25.01-45.19 | 0.25-0.61 | 65.0-65.5 | Y2.IV,V,VI,VII |
|  |  |  |  |  |  |  | cqGL7-1 | 7H | 66 | 4.22-8.07 | 9.08-27.07 | 0.09-0.26 | 65.71-66.28 | Y1.ΔT6/Y2.ΔT3,ΔT5 |
|  | uqGL7-2 | 92.5 | 7HL_27996637 | 92.14-92.85 | C |  | cqGL7-2 | 7H | 92.5 | 4.37-12.36 | 12.70-38.03 | 0.06-0.11 | 92.14-92.85 | Y1.ΔT7/Y2.ΔT7 |
|  | uqGL7-3 | 109 | 7HS_35580690 | 108.5-110.5 | C |  | cqGL7-3 | 7H | 109 | 3.22 | 12.19 | -0.11 | 108.5-110.5 | Y2.ΔT4 |
|  | uqGL7-4 | 116 | 5_496886371 | 115.5-116.5 | U |  | ucqGL7-2 | 7H | 116 | 10.26 | 21.79 | 0.27 | 115.5-116.5 | Y2.III |
|  | uqGL7-5 | 124 | 7HS_32890650 | 123.5-124.5 | C |  | cqGL7-4 | 7H | 124 | 3.20 | 12.31 | 0.22 | 123.5-124.5 | Y1.ΔT3 |
|  | uqGL7-6 | 136 | 7HS_29196961 | 135.64 - 136.35 | U |  | ucqGL7-3 | 7H | 136 | 28.14-38.74 | 23.52-27.2 | -0.48 to -0.37 | 135.64-136.35 | Y2.VI,VII |
|  | uqGL7-7 | 140 | 7_319506952 | 138.5-140.5 | U |  | ucqGL7-4 | 7H | 140 | 22.26 | 37.83 | 0.40 | 138.5-140.5 | Y1.III |
|  | uqGL7-8 | 150.92 | Bmac31 | 150.64 - 151.2 | U |  | ucqGL7-5 | 7H | 150.92 | 6.67-24.79 | 17.10-43.21 | 0.35-0.45 | 150.64-151.2 | Y1.V,VI,VII/Y2.II |
|  | uqGL7-9 | 175 | 7_144173681 | 174.5-175.5 | U |  | ucqGL7-6 | 7H | 175 | 3.20 | 7.47 | 0.22 | 174.5-175.5 | Y1.II |
| GW | uqGW1-1 | 19.76 | 1H_45582581 | 19.42 - 20.09 | U |  | ucqGW1-1 | 1H | 19.76 | 5.08-7.31 | 2.71-5.45 | -0.09 to -0.06 | 19.42-20.09 | Y1.IV,V,/Y2.V,VII |
|  | uqGW1-2 | 48 | 1_167351152 | 47.5 - 48.5 | U |  | ucqGW1-2 | 1H | 48 | 6.47 | 3.25 | -0.06 | 47.5-48.5 | Y2.VI |
|  | uqGW2-1 | 53 | 2HL_17013042 | 52.5-53.5 | C |  | cqGW2-1 | 2H | 53 | 4.08 | 15.00 | -0.06 | 52.5-53.5 | Y1.ΔT6 |
|  | uqGW2-2 | 86 | M_207663_1931 | 85.5-86.5 | C |  | cqGW2-2 | 2H | 86 | 3.82 | 12.43 | -0.05 | 85.5-86.5 | Y2.ΔT6 |
|  | uqGW2-3 | 116 | 2HL_13832944 | 115.5-116.5 | C |  | cqGW2-3 | 2H | 116 | 5.00 | 18.33 | 0.06 | 115.5-116.5 | Y2.ΔT5 |
|  | uqGW2-4 | 125.27 | 2HL_22930294 | 125.13-125.4 | B |  | ucqGW2 | 2H | 124.69 | 8.21-53.91 | 32.27-75.51 | 0.09-0.39 | 124.44-125.25 | Y1.II,III,IV,V,VI,VII/Y2.II,III,IV,V,VI,VII |
|  |  |  |  |  |  |  | cqGW2-4 | 2H | 126 | 4.29-23.87 | 13.33-55.68 | 0.05-0.16 | 125.75-126.25 | Y1.ΔT3,ΔT4/Y2.ΔT3,ΔT4 |
|  | uqGW3-1 | 26.99 | 3_529115904 | 26.7-26.88 | B |  | ucqGW3-1 | 3H | 26 | 7.01 | 3.06 | 0.07 | 25.5-26.5 | Y1.VI |
|  |  |  |  |  |  |  | cqGW3-1 | 3H | 27.5 | 3.09-4.08 | 9.85-13.72 | -0.04 to 0.06 | 27.14-27.85 | Y1.ΔT5/Y2.ΔT3 |
|  | uqGW3-2 | 31.58 | 3HL_15958290 | 31.34-31.82 | B |  | ucqGW3-2 | 3H | 31.33 | 3.62-4.75 | 2.16-6.75 | -0.07 to 0.06 | 31.04-31.62 | Y1.III/Y2.VI,VII |
|  |  |  |  |  |  |  | cqGW3-2 | 3H | 32.19 | 4.74-6.31 | 16.11-20.59 | -0.09 to 0.06 | 31.75-32.64 | Y1.ΔT3/Y2.ΔT6 |
|  | uqGW3-3 | 52 | 3HL_45910009 | 51.5-52.5 | U |  | ucqGW3-3 | 3H | 52 | 3.53 | 1.43 | -0.05 | 51.5-52.5 | Y1.VI |
|  | uqGW3-4 | 83 | 3HL_3720522 | 82.5-83.5 | C |  | cqGW3-3 | 3H | 83 | 3.07 | 10.93 | -0.05 | 82.5-83.5 | Y1.ΔT6 |
|  | uqGW3-5 | 98 | 3_219109045 | 96.5-98.5 | C |  | cqGW3-4 | 3H | 98 | 4.17 | 15.39 | -0.05 | 96.5-98.5 | Y2.ΔT5 |
|  | uqGW4-1 | 52 | 4_474989327 | 51.64 - 52.35 | U |  | ucqGW4 | 4H | 52 | 4.09-4.48 | 1.18-2.32 | -0.05 | 51.64-52.35 | Y2.IV,VII |
|  | uqGW5-1 | 4 | 5_3871196 | 1.5-5.5 | C |  | cqGW5 | 5H | 4 | 3.71 | 11.83 | -0.05 | 1.5-5.5 | Y1.ΔT5 |
|  | uqGW6-1 | 25 | 6_518846666 | 23.5-25.5 | U |  | ucqGW6 | 6H | 25 | 3.79 | 1.63 | -0.05 | 23.5-25.5 | Y1.VII |
|  | uqGW6-2 | 32 | 6_518728726 | 31.5-33.5 | C |  | cqGW6 | 6H | 32 | 3.22 | 11.98 | -0.04 | 31.5-33.5 | Y2.ΔT7 |
|  | uqGW7-1 | 57 | 1_4088556 | 56.5-57.5 | U |  | ucqGW7-1 | 7H | 57 | 5.82 | 4.28 | 0.08 | 56.5-57.5 | Y1.IV |
|  | uqGW7-2 | 65.13 | 7HL_13143105 | 64.78-65.66 | U |  | ucqGW7-2 | 7H | 65.13 | 7.63-46.68 | 5.61-34.11 | -0.18 to 0.26 | 64.78-65.66 | Y1.IV,V,VII/Y2.IV,V,VI |
|  | uqGW7-3 | 130 | 7HS_25905506 | 129.5-130.5 | C |  | cqGW7 | 7H | 130 | 3.93 | 7.76 | 0.07 | 129.5-130.5 | Y1.ΔT4 |
| GD | uqGD1-1 | 22 | 1_306394013 | 21.64 - 22.35 | U |  | ucqGD1-1 | 1H | 22 | 3.94-4.05 | 8.34-9.90 | -0.11 to -0.10 | 21.64-22.35 | Y1.II/Y2,II |
|  | uqGD1-2 | 42 | 1H_10863328 | 41.5-42.5 | U |  | ucqGD1-2 | 1H | 42 | 4.14 | 5.92 | -0.09 | 41.5-42.5 | Y2.III |
|  | uqGD1-3 | 64 | M_2579923_225 | 62.5-64.5 | U |  | ucqGD1-3 | 1H | 64 | 16.54 | 7.09 | 0.12 | 62.5-64.5 | Y1,IV |
|  | uqGD1-4 | 89.17 | Bmag770 | 88.89 - 89.46 | U |  | ucqGD1-4 | 1H | 89.17 | 4.37-27.03 | 1.23-14.59 | -0.18 to 0.12 | 88.89-89.46 | Y1.IV,V/Y2.VII |
|  | uqGD2-1 | 96 | 2HL_43859802 | 95.5-96.5 | C |  | cqGD2-1 | 2H | 96 | 4.24 | 13.74 | 0.11 | 95.5-96.5 | Y1.ΔT3 |
|  | uqGD2-2 | 127.66 | 2_522610509 | 127.45-127.86 | B |  | ucqGD2 | 2H | 125.93 | 8.35-56.41 | 18.90-78.03 | 0.18-0.42 | 125.69-126.44 | Y1.II,III,IV,V,VI,VII/Y2.II,III,IV,V,VI,VII |
|  |  |  |  |  |  |  | cqGD2-2 | 2H | 129 | 6.05-6.11 | 18.55-20.41 | 0.10-0.11 | 128.64-129.35 | Y1.ΔT4/Y2.ΔT4 |
|  | uqGD3-1 | 27 | 3_529115904 | 26.64-27.35 | B |  | ucqGD3-1 | 3H | 27 | 4.19 | 1.96 | 0.06 | 26.5-27.5 | Y1.VI |
|  |  |  |  |  |  |  | cqGD3 | 3H | 27 | 3.71 | 10.91 | 0.05 | 26.5-27.5 | Y1.ΔT5 |
|  | uqGD3-2 | 32 | 3HL_15958290 | 31.5-32.5 | U |  | ucqGD3-2 | 3H | 32 | 9.02 | 2.93 | 0.09 | 31.5-32.5 | Y2.VII |
|  | uqGD3-3 | 90 | 3HL_14205585 | 89.64 - 90.35 | U |  | ucqGD3-3 | 3H | 90 | 4.49-4.78 | 2.29-2.46 | -0.07 to -0.06 | 89.64-90.35 | Y1.VI,VII |
|  | uqGD4-1 | 12 | 4_528451537 | 11.5-12.5 | U |  | ucqGD4-1 | 4H | 12 | 4.22 | 1.22 | -0.05 | 11.5-12.5 | Y2.V |
|  | uqGD4-2 | 52 | 4_474989327 | 51.5-52.5 | U |  | ucqGD4-2 | 4H | 52 | 4.59 | 1.39 | -0.05 | 51.5-52.5 | Y2.VII |
|  | uqGD4-3 | 152 | 4_16819133 | 151.5-154.5 | C |  | cqGD4 | 4H | 152 | 3.31 | 9.05 | -0.04 | 151.5-154.5 | Y1.ΔT7 |
|  | uqGD5-1 | 9 | 5_51657943 | 8.5-10.5 | C |  | cqGD5-1 | 5H | 9 | 3.04 | 9.30 | 0.04 | 8.5-10.5 | Y1.ΔT7 |
|  | uqGD5-2 | 17 | GBM1176 | 11.5-24.5 | C |  | cqGD5-2 | 5H | 17 | 3.08 | 9.62 | -0.04 | 11.5-24.5 | Y1.ΔT5 |
|  | uqGD5-3 | 102 | 5HS_16446198 | 100.5-102.5 | U |  | ucqGD5-1 | 5H | 102 | 11.71 | 3.87 | 0.10 | 100.5-102.5 | Y2.V |
|  | uqGD5-4 | 123.5 | 5HS_4157152 | 122.79 - 124.2 | U |  | ucqGD5-2 | 5H | 123.5 | 4.05-21.31 | 1.12-8.72 | -0.14 to -0.05 | 122.79-124.2 | Y2.V,VII |
|  | uqGD5-5 | 155 | M_81421_1318 | 154.5-156.5 | U |  | ucqGD5-3 | 5H | 155 | 5.41 | 2.71 | -0.07 | 154.5-156.5 | Y1.VI |
|  | uqGD6-1 | 78 | GBM1256 | 77.5-78.5 | U |  | ucqGD6 | 6H | 78 | 5.54 | 1.66 | 0.06 | 77.5-78.5 | Y2.V |
|  | uqGD7-1 | 57.6 | 7_523855164 | 57.15-58.04 | C |  | cqGD7-1 | 7H | 57.6 | 3.14-3.36 | 10.74-12.89 | 0.09 | 57.15-58.04 | Y1.ΔT3/Y2.ΔT3 |
|  | uqGD7-2 | 65.88 | 7HL_37199773 | 65.63 - 66.12 | U |  | ucqGD7-1 | 7H | 65.88 | 13.71-44.92 | 10.08-31.65 | 0.13-0.26 | 65.63-66.12 | Y1.VI/Y2.IV,V,VI,VII |
|  | uqGD7-3 | 70 | M_363857_407 | 69.5-70.5 | C |  | cqGD7-2 | 7H | 70 | 10.99 | 34.69 | 0.09 | 69.5-70.5 | Y2.ΔT7 |
|  | uqGD7-4 | 76.8 | 7HL_6800849 | 76.35-77.24 | B |  | ucqGD7-2 | 7H | 76 | 17.85 | 31.06 | 0.21 | 75.5-76.5 | Y2.III |
|  |  |  |  |  |  |  | cqGD7-3 | 7H | 80 | 6.18 | 17.99 | 0.06 | 78.5-80.5 | Y1.ΔT7 |
|  | uqGD7-5 | 94 | Bmag746 | 93.5-94.5 | U |  | ucqGD7-3 | 7H | 94 | 10.79 | 3.43 | 0.09 | 93.5-94.5 | Y2.VII |
|  | uqGD7-6 | 151 | Bmac31 | 150.5-151.5 | U |  | ucqGD7-4 | 7H | 151 | 9.59 | 6.33 | 0.11 | 150.5-151.5 | Y2.VI |
|  | uqGD7-7 | 167 | Bmag900 | 166.5-167.5 | U |  | ucqGD7-5 | 7H | 167 | 5.46 | 3.04 | 0.08 | 166.5-167.5 | Y1.VII |
